# Transgenerational chromosome repair in the asexual bdelloid rotifer *Adineta vaga*

**DOI:** 10.1126/sciadv.aee0891

**Published:** 2026-09-11

**Authors:** Antoine Houtain, Marc Llirós Dupré, Boris Hespeels, Émilien Nicolas, Paul Simion, Julie Virgo, Anne-Catherine Heuskin, Thomas Lenormand, Bernard Hallet, Karine Van Doninck

**Affiliations:** ^1^ Molecular Biology and Evolution (MBE), Université libre de Bruxelles (ULB), Brussels 1050, Belgium.; ^2^ Research Unit in Environmental and Evolutionary Biology (URBE), Institute of Life, Earth and Environment (ILEE), University of Namur, Namur 5000, Belgium.; ^3^ Biosciences Department, Universitat de Vic–Universitat Central de Catalunya, Vic 08500, Spain.; ^4^ LIBST, Université Catholique de Louvain, Louvain-la-Neuve 1348, Belgium.; ^5^ EcoBio-Ecosystems, Biodiversity, Evolution, Université de Rennes 1, Rennes 35042, France.; ^6^ NARILIS, Université de Namur, Namur 5000, Belgium.; ^7^ LARN, Université de Namur, Namur 5000, Belgium.; ^8^ CEFE, Univ Montpellier, CNRS, Univ Paul Valéry Montpellier 3, Montpellier 34293, France.

## Abstract

Homologous recombination is an essential DNA repair mechanism that also promotes chromosome pairing and ensures allele segregation during meiosis in sexual organisms. Here, we explore the dual function of homologous recombination in the bdelloid rotifer *Adineta vaga*, an asexual species known for its remarkable resilience to extreme genotoxic stresses. Genomic analyses reveal that *A. vaga* uses meiotic recombination to promote spontaneous crossovers and gene conversion during oogenesis and to repair the genome in response to DNA damage. The data also support a model of transgenerational DNA repair, termed break-induced homologous extension repair (BIHER), in which broken chromosomes are progressively restored over multiple generations. Our findings suggest that meiotic BIHER, coupled with the holocentric structure of chromosomes, may represent a key adaptation of life in extreme environments.

## INTRODUCTION

Homologous recombination (HR) is a universal mechanism that functions as a high-fidelity DNA repair process of double-strand breaks (DSBs). Defects in HR generally result in cell death or severe dysfunction, including the development of cancer (*1*). In sexual organisms, HR repair of programmed DSBs induced during meiosis is necessary for proper pairing and segregation of homologous chromosomes (*2*). Moreover, meiotic HR generates genetic variability by reassorting alleles along the chromosomes, limiting selective interference between loci and improving the response to selection (*3*–*5*).

In this study, we investigated the evolutionary and DNA repair functions of HR in the context of an asexual and extremo-tolerant organism, the bdelloid rotifer *Adineta vaga*. These microscopic animals are asexual and have diversified into hundreds of morphospecies (*6*) over millions of years without any evidence of males or hermaphrodites (*7*). They thrive in limno-terrestrial environments, such as lichens and mosses, which frequently dry out (*6*). In turn, they display a remarkable tolerance to extreme conditions, including complete desiccation and high doses of ionizing radiation (IR) that cause hundreds of DSBs along the chromosomes (*8*–*11*). This exceptional resistance likely results from a combination of adaptations, including high antioxidant levels (*11*), eutely (a fixed number of ∼1000 somatic cells in adults), and the restriction of mitosis to embryonic development (*12*). However, the capacity for rapid and efficient DNA repair appears central to their recovery from extensive genomic damage. Our recent work on *A. vaga* revealed that desiccation and IR trigger rapid somatic DNA repair immediately upon rehydration, proceeding over a 48-hour period (*13*, *14*). Transcriptomic and proteomic studies have shown that this early response is primarily driven by nonhomologous end-joining (NHEJ) and base excision DNA repair pathways (*11*, *15*).

In contrast, DNA repair in the germ line follows a distinct temporal pattern. In *A. vaga*, it has been shown that oogenesis proceeds through a modified meiosis I during which homologous chromosomes pair but do not segregate, producing unreduced diploid eggs (*14*). Germline DNA repair in response to IR is specifically activated during this meiotic-like stage, when chromosome pairing occurs, suggesting that the meiotic recombination machinery could be key to restoring genome integrity in oocytes (*14*). Moreover, population genomic studies have revealed indirect signatures of recombination, such as reduced linkage disequilibrium and homozygous regions, indicating that HR-like processes may shape bdelloid genomes despite their asexuality (*16*–*20*). From an evolutionary perspective, maintaining germline genome integrity is essential. Although recent studies hint that meiotic HR may contribute to bdelloid long-term evolution, the precise mechanisms of germline DNA repair remain unknown, and it is unclear whether genomic changes persist over generations (*20*).

Here, we surveyed the genome dynamics of *A. vaga* lines in the course of a microevolution (ME) experiment spanning ∼75 to ∼135 generations, with bottlenecks at each generation, following the design of a mutation accumulation experiment (*21*). Parallel lineages were maintained hydrated or periodically desiccated (fig. S1). The role of HR in genome repair (GR) was examined by inducing acute DNA damage through prolonged desiccation or proton IR treatment (fig. S2). In both experiments, the genomes of descendants were compared to the ancestor. Allele frequency (AF) at heterozygous sites was used to detect HR signatures as losses of heterozygosity (LOH) tracts, and combining AF with coverage depth allowed the identification of large deletions and duplications.

## RESULTS

### Meiotic recombination in the germ line

**Fig. 1.**
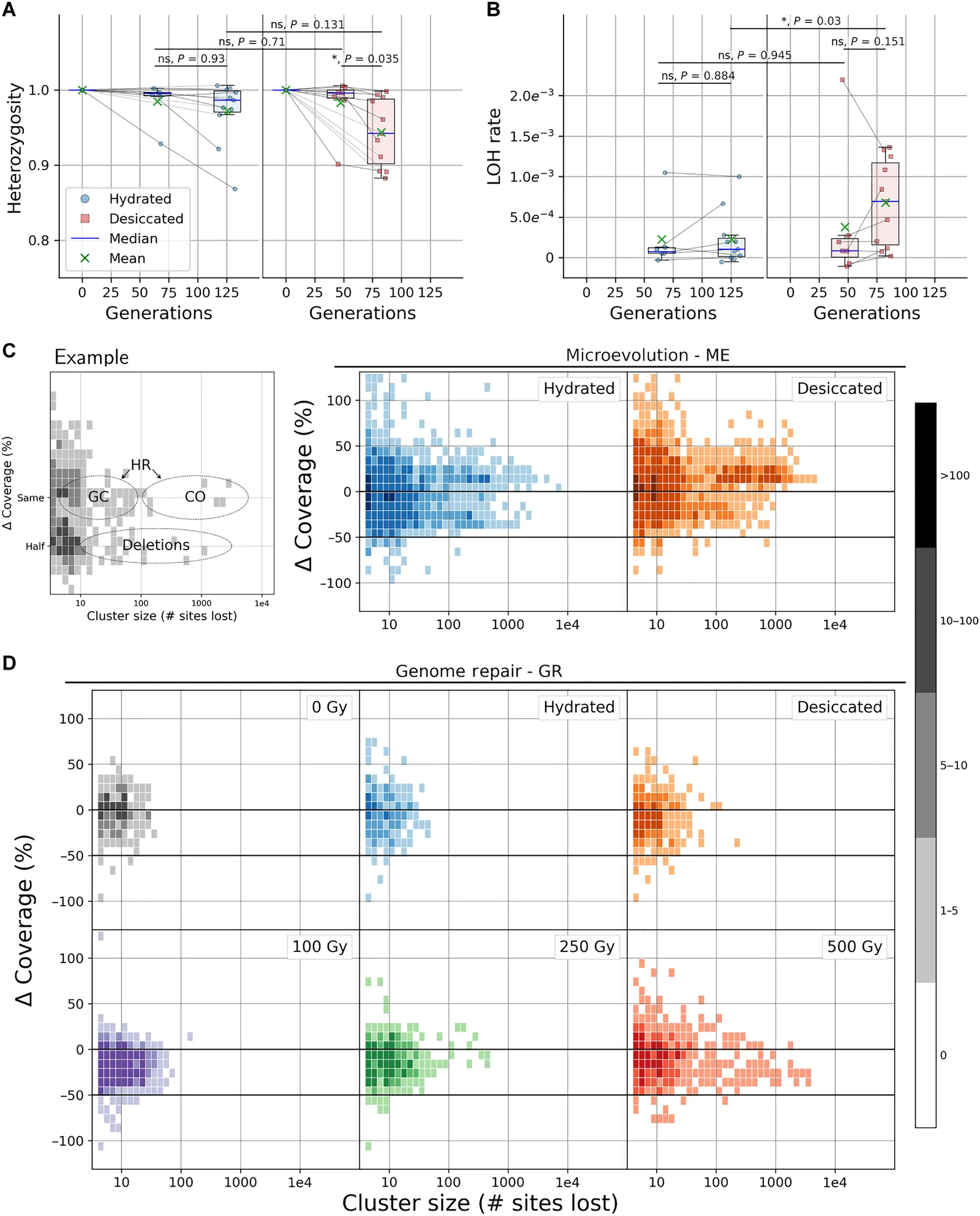
Accumulation of LOH in ME and GR samples. (**A**) Heterozygosity over generations in both hydrated and desiccated ME samples. Desiccated samples at endpoint have a significantly lower average than at midpoint [Mann-Whitney *U* (MWU) rank test; *P* = 0.0346; rank biserial *r* = −0.61]. (**B**) LOH rate over generations in these ME samples. LOH rate at endpoint is significantly higher in desiccated samples than in hydrated samples (MWU; *P* = 0.0302; rank biserial *r* = 0.55). (**C** and **D**) 2D histograms showing the size distribution of LOH clusters. The size of a cluster corresponds to the number of ancestrally heterozygous sites that were lost in the offspring (only clusters larger than five contiguous sites were considered). The *x* axis is plotted on a logarithmic scale. Each cluster change of coverage is indicated by the Δ coverage (%; *y* axis). Color intensity indicates frequency (*z* axis). Clusters from each condition were aggregated across samples. In (C), a randomly generated example histogram is shown to illustrate where LOH clusters resulting from deletions and HR would appear (dashed ellipses). ns, not significant.

The ancestral laboratory strain of *A. vaga* has a diploid genome structure containing six pairs of homologous chromosomes with an average sequence divergence of 1.7% (*19*). In the ME experiment, 11 lines remained hydrated throughout, while another 11 lines experienced 4 cycles of 10-day desiccation (fig. S1 and table S1). All lines were sequenced at the endpoint of the experiment (after ∼75 to ∼135 generations for desiccated and hydrated lines, respectively). In addition, 14 of these lines were sequenced at the experiment midpoint (after ∼40 to ∼70 generations, respectively). Selection was reduced by collecting a single descendant at each generation. For both hydrated and desiccated lines, average heterozygosity decreased with the number of generations, resulting in an average loss of 4.23% of ancestrally heterozygous sites at endpoint. The difference between midpoint and endpoint is statistically significant in desiccated lines (Fig. 1A). Moreover, desiccated samples accumulated LOH significantly faster than hydrated samples (Fig. 1B). While the variance of LOH rate appears to increase over time in both hydrated and desiccated lines, we found no significant differences in LOH rate between time points (Fig. 1, A and B).

In these 22 ME lines, LOH clusters were detected (Fig. 1C). The size of these clusters was determined by the number of contiguous heterozygous sites lost, and their coverage level was measured by the normalized sequencing depth within each cluster. Clusters with fewer than five contiguous sites were ignored, as they could arise from multiple independent LOH events rather than from a single genetic event (fig. S3). Most of the LOH clusters (85.2% of all clusters) in ME are smaller than 100 sites and correspond to LOH tracts that span, on average, less than 5 kb. These short clusters (<100 sites) account for only 14.2% total LOH, while larger clusters (>100 sites, 14.8% of the clusters) account for 85.8% of the total LOH. In addition, 19.4% of these large clusters (>100 sites) correspond to putative deletions (Δ coverage below −20) and make up 11.4% of total LOH (Fig. 1C).

**Fig. 2.**
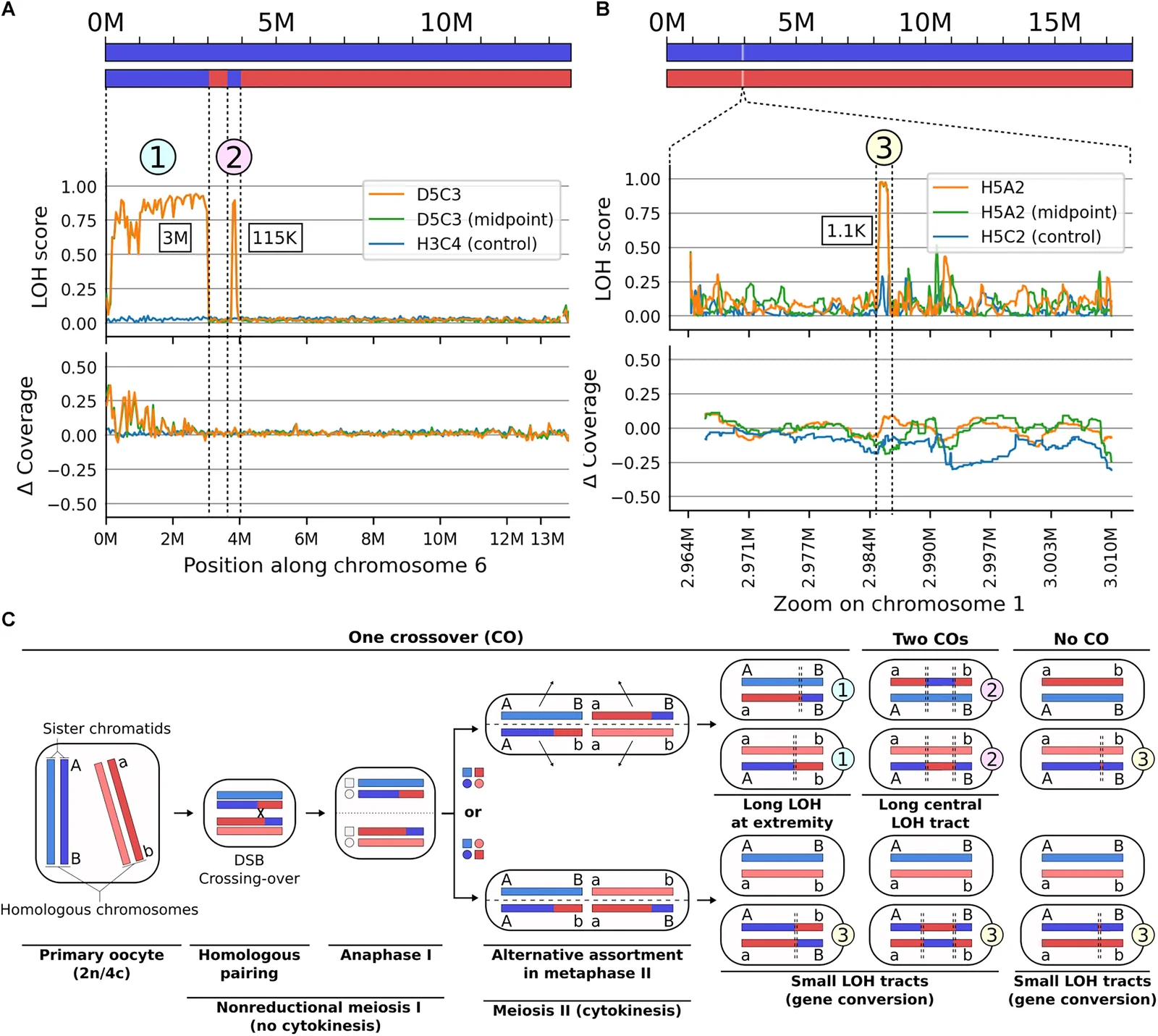
Examples of HR-induced LOH tracts observed in the genome of *A. vaga* after multiple generations in the ME experiment. (**A**) LOH score and Δ coverage along chromosome 6 of ME sample D5C3 at midpoint (green line) and endpoint (orange line). Examples of LOH tracts resulting from one CO in (1) or two COs in (2). The ME sample H3C4 is a negative control (blue line) without LOH on chromosome 6. (**B**) LOH score and Δ coverage along a small portion of chromosome 1 in ME sample H5A2 at midpoint (green line) and endpoint (orange line). Example of a LOH tract likely formed through GC in (3). The ME sample H5C2 is a negative control (blue line) without LOH in the considered region. (**C**) Illustration of oogenesis through a nonreductional meiosis I, showing expected LOH tracts following HR with reciprocal exchange or GC. Potential outcomes are given with one, two, or no COs. In some instances, depending on how recombinant chromatids segregate, the same initial CO can result in either a GC (3) or a long terminal LOH (1).

In all samples, AF (fig. S4) and coverage are more variable toward the ends of the chromosome due to a higher content of repetitive DNA (*19*) (as exemplified for chromosome 6 in fig. S5). Sequencing reads tend to misalign between paralogous copies of repetitive sequences, creating artifactual heterozygosity signal and deviation in coverage. To reduce this noise, we calculated a LOH score by measuring the AF difference between the ancestor and offspring over a 50- to 100-kb window, corresponding to 1/250 of the chromosome length. A LOH score near 1.0 indicates a LOH event, while a LOH score around 0.0 suggests no detectable change (fig. S4B). Long LOH tracts, ranging from 50 kb to over 1 Mb, were identified in 7 of 11 hydrated lines and 4 of 11 desiccated lines (fig. S6). Some of these tracts extend to the chromosome ends, while others are interrupted. For instance, Fig. 2A shows a LOH tract of ∼3 Mb (1) located at the extremity of chromosome 6 in sample D5C3 and another of ∼115 kb (2) within an internal region, both detected at the midpoint and endpoint of the experiment. In addition, smaller LOH tracts (<50 kb) were identified at both sequencing time points, as exemplified by tract (3) in Fig. 2B (other examples of short LOH tracts are given in fig. S7).

These LOH tracts show no decrease in coverage and are consistent with HR taking place during the modified meiosis of *A. vaga* when homologous chromosomes assemble into bivalents (*14*). Long LOH tracts extending to the chromosome tips are consistent with a single crossing-over (CO) (Fig. 2C, case 1), while long internal LOH tracts likely arose from a double CO (Fig. 2C, case 2). On the other hand, short LOH tracts (from ∼100 bp to a few kilobases) likely correspond to gene conversion (GC) events inherent to the mechanism of HR, even in the absence of CO (Fig. 2C, case 3).

All identified long LOH tracts (>50 kb) at midpoint are also retrieved in the endpoint sequencing, except for samples D2B3 and D2C1, where a LOH cluster is found at the midpoint but not at the endpoint (fig. S8). These rare cases are likely due to HR that occurred at the bottleneck, shortly after the separation of two individuals, with one being used to initiate culture for midpoint sequencing and the other to continue propagation of the ME line.

### Inheritance of structural variations in ME

**Fig. 3.**
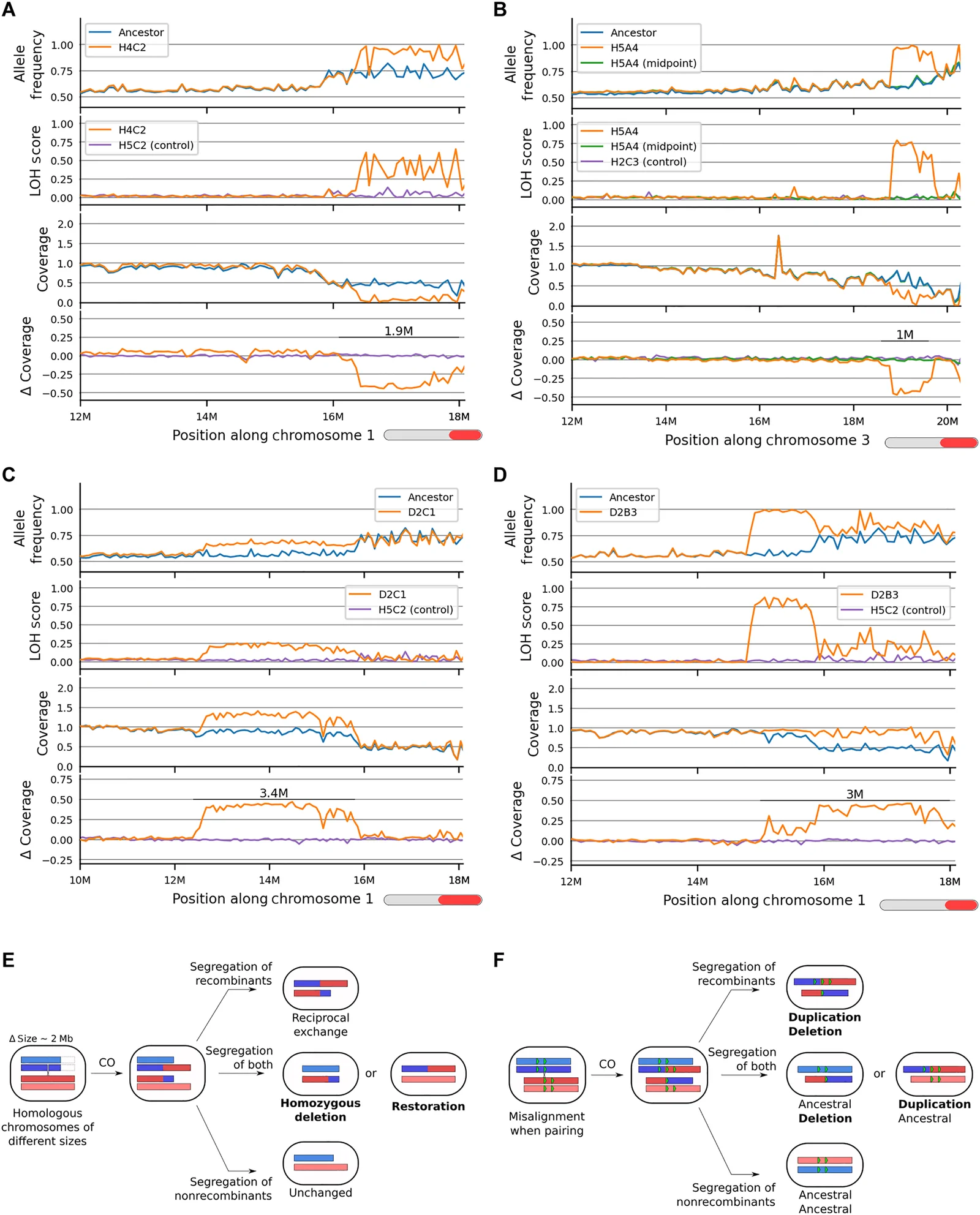
Large deletions and duplications in the genome of *A. vaga* after multiple generations in the ME experiment. (**A** to **D**) AF, LOH score, coverage, and Δ coverage across nonoverlapping windows at chromosome ends. Retention of sequence coverage confirmed hemizygous deletions, while homozygous deletions were characterized by complete loss of coverage. (A) Homozygous deletion on chromosome 1 in sample H4C2 (72.6-kb windows). (B) Hemizygous deletion on chromosome 3 in sample H5A4 (81.4-kb windows). (C) Duplication of a region on chromosome 1 in sample D2C1 (72.6-kb windows). (D) Restoration of diploidy in a region with an ancestral deletion on chromosome 1 in sample D2B3 (72.6-kb windows). (**E**) Theoretical model of HR between chromosomes of unequal sizes (e.g., chromosome 1 of *A. vaga*). Crossovers can generate homozygous deletions or restore coverage at chromosome ends, depending on how recombinant chromatids segregate. (**F**) Theoretical model of HR between homologous chromosomes misaligned within repetitive regions, potentially leading to segmental duplications or deletions.

Besides signatures of reciprocal exchanges between homologous chromosomes, the genomes of ME lines exhibit regions where changes in the LOH score are associated with variations in coverage compared to the ancestor (fig. S4B and table S2). LOH tracts with a decrease in coverage correspond to deletions, as exemplified on chromosomes 1 and 3 of ME lines H4C2 and H5A4, respectively (Fig. 3, A and B). Furthermore, deletions are categorized as homozygous when both parental alleles are absent (Fig. 3A) or hemizygous if one allele is absent (Fig. 3B and fig. S4B). Tracts where coverage increases correspond to duplications, as exemplified by chromosome 1 of lines D2C1 and D2B3 (Fig. 3, C and D). Duplications may represent either the addition of extra copies of specific chromosomal regions (Fig. 3C) or the restoration of diploidy in regions previously affected by ancestral deletions (Fig. 3D and fig. S4B).

These homozygous deletions and restoration of diploidy likely result from a single CO between homologous chromosomes of different lengths (Fig. 3E). The exact mechanisms behind hemizygous deletions and other duplications remain less clear, one possibility being non-allelic homologous recombination (NAHR), also known as “unequal” crossovers (Fig. 3F) (*22*).

**Fig. 4.**
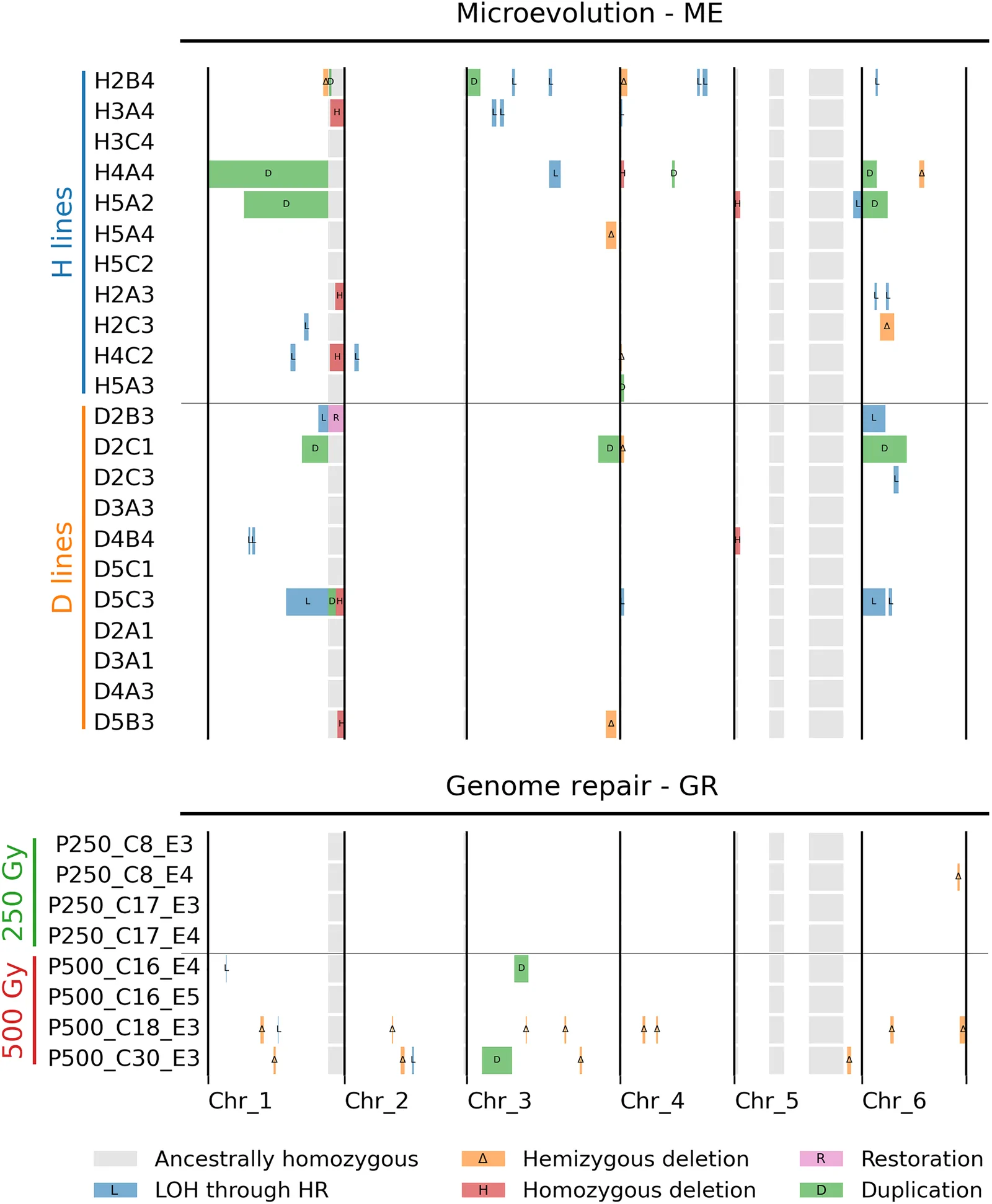
Summary of the positions of large (>100 kb) events observed in the genome of *A. vaga* in ME and GR samples. Events are shown per sample and chromosome (based on figs. S6 and S14). GR samples from the following treatments are not shown, as they do not display large events (>100 kb): 30 days hydrated (30H), 30 days desiccated (30D), 0 Gy IR (P0), and 100 Gy IR (P100).

In the 22 sequenced ME lines, we identified 11 large (>50 kb) duplications (including one case of diploidy restoration) and 16 large (>50 kb) deletions, both homozygous and hemizygous, across 13 different *A. vaga* lines (8 hydrated and 5 desiccated). These large genomic events in ME are not randomly distributed but tend to cluster near the chromosome ends (Fig. 4 and fig. S6), where the density of transposable elements is higher (fig. S9) (*19*). In addition, samples with recombination signatures accumulated more events than would be expected if they occurred independently ($\chi^{2}=112.8$; $P=5.02e^{-19}$) (Fig. 4), suggesting that these recombination signatures are not acquired independently.

Several deletions (3 of 11) and duplications (2 of 12) were detected at both the midpoint and endpoint of sequencing (fig. S6), indicating that these events occurred early in the ME experiment and were transmitted over generations. However, some regions showed further modifications over time, with changes in Δ coverage present at the endpoint but not at the midpoint. This suggests ongoing recombination within specific chromosomal regions as observed on chromosomes 1 and 6 of line H4A4. In this line, the changes in AF and coverage are even more complex. They point to a whole genome duplication (WGD) followed by an additional duplication of chromosome 1, resulting in aneuploidy, maintained through generations (fig. S10).

### Meiotic HR mediates GR

In the ME experiment, 10-day desiccation events slightly increased the rate of LOH in *A. vaga* lines, suggesting that desiccation-induced DSBs enhanced HR activity in the germ line. To investigate whether HR plays a role in maintaining genome integrity in the germ line under more severe DNA damaging conditions, *A. vaga* cultures were subjected to longer periods of desiccation [30 days, estimated at ∼15 to 20 DSBs per genome (*13*)] or irradiated with increasing doses of proton IR. After treatment, individual mothers were isolated, their first two eggs discarded, and only third-or-later eggs were collected and cultured, ensuring that they were undifferentiated at exposure. The resulting clonal population was sequenced, providing lineage-resolved information (fig. S2). The IR doses used were 100, 250, and 500 Gy of proton IR, causing theoretically ∼88, 220, and 440 DSBs per genome, respectively (*13*).

To assess the impact of IR and long-term desiccation on fertility, we isolated treated mothers and measured growth rates by estimating the population size increase between two time points (fig. S11). Although total adult counts differed significantly in most pairwise comparisons (fig. S12, A and B), these differences were largely due the age and maturity of founder individuals at the time of isolation. Once adjusted, most treatments showed only minor effects on fertility, and the most significant difference in growth rates was observed at the highest IR dose (0 Gy compared to 500 Gy, *t* tests, $P=4.59e^{-5}$, Glass’ δ = 0.613; fig. S12C). Together, these results confirm *A. vaga*’s remarkable resistance to genotoxic stresses, being able to produce a viable progeny after extreme genotoxic treatment.

The distributions of LOH clusters in nonirradiated (0 Gy) and nondesiccated (hydrated) samples mostly consist of short tracts with fewer than 100 sites, distributed around a Δ coverage of 0 (Fig. 1D). As discussed above, these tracts are likely the result of GC. Their presence in untreated samples confirms that recombination between homologous chromosomes occurs independently of external stressors, and the increase in GC signatures in ME lines compared to GR samples shows their accumulation over generations (Fig. 1, C and D). The 30-day desiccated cultures have slightly more LOH than the hydrated line, with two LOH clusters exceeding 100 contiguous sites, but the difference in LOH accumulation is not significant (table S3). By contrast, IR with 100, 250, and 500 Gy of protons resulted in a dose-dependent amplification of LOH [ordinary least-squares (OLS), $P=1.18e^{-3}$], with 39% of LOH attributable to HR repair. In pairwise comparisons, only the 500-Gy treatment significantly increased LOH compared to the 0-Gy controls (Tukey, *P* = 0.0042; Fig. 1D, fig. S13, and table S3). The large LOH clusters (>100 contiguous sites) observed in the 500-Gy samples are distributed among different chromosomes within a same sample (Fig. 4 and fig. S14), and their positions along the chromosomes statistically differ from those observed in the ME lines (fig. S15). In ME lines, the density of clusters increases toward the ends of the chromosomes, whereas in GR 500-Gy samples, clusters are evenly scattered, reflecting the stochasticity of IR-induced DNA damage (Fig. 4 and fig. S9).

### Transgenerational repair of large intrachromosomal deletions

A large proportion of IR-induced LOH clusters are distributed around low coverage (Δ coverage below −20), indicating that they correspond to deletions (Fig. 1D). The size of these IR-induced deletions increases with the IR dose (fig. S13), with the 500-Gy IR lines exhibiting multiple large hemizygous deletions, involving the loss of genomic regions up to 400 kb (Fig. 4 and fig. S14).

**Fig. 5.**
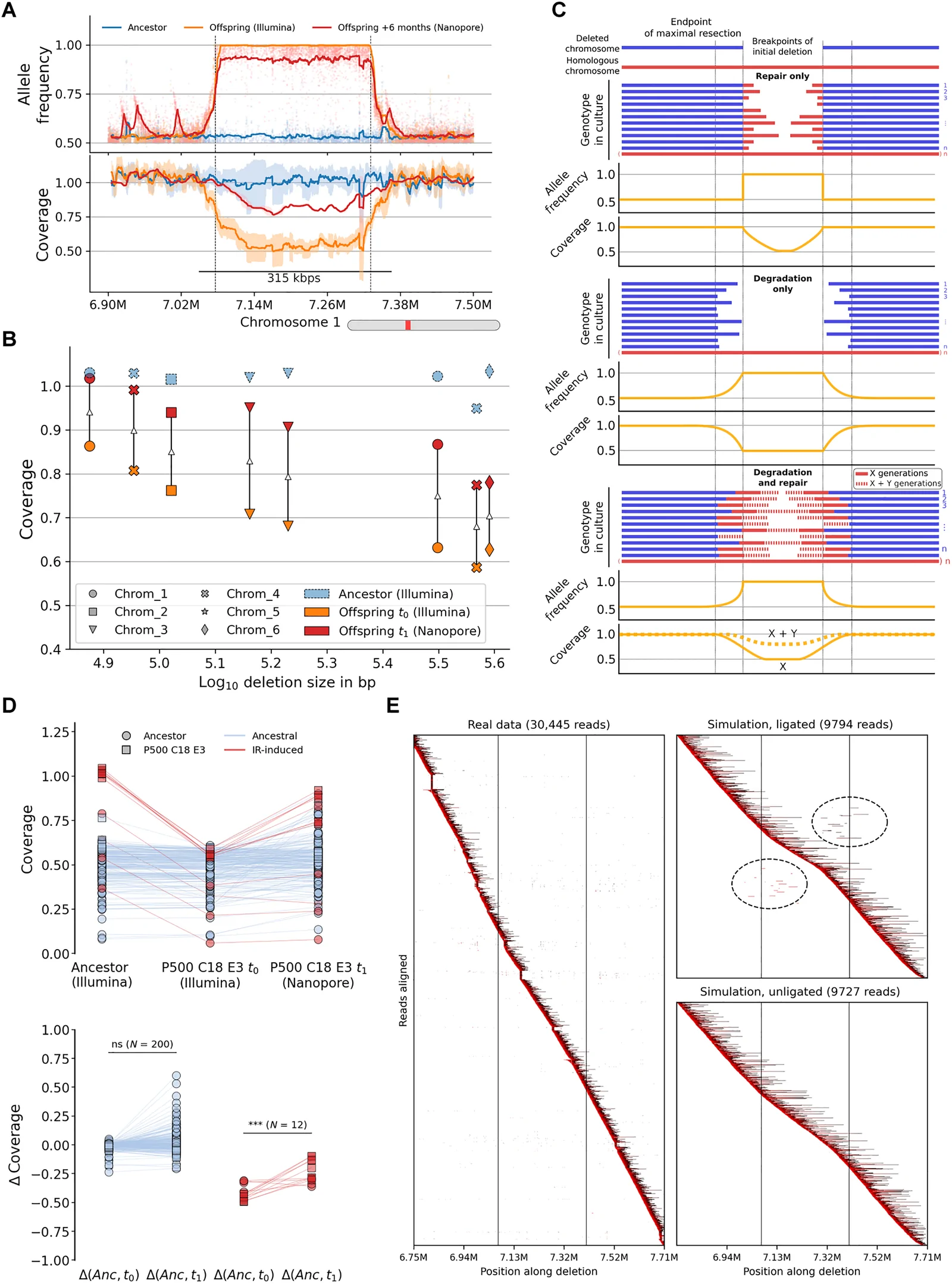
Progressive DNA repair of fragmented chromosomes in the GR sample P500 C18 E3. (**A**) AF and coverage across a deleted region of chromosome 1. (**B**) Coverage (*y* axis) by deletion size (log-scaled *x* axis) for ancestor (blue), P500 C18 E3 $t_{0}$ after 1 month (Illumina, orange), and $t_{1}$ after 6 months of culture (Nanopore, red). (**C**) Expected AF and coverage patterns under three scenarios: repair only (abrupt AF shift), degradation only (AF-coverage symmetry), and a combination of repair and degradation (AF variability from degradation, coverage increase from repair). The orange dashed line indicates coverage progression over culture time. (**D**) Top: normalized coverage across ancestral deletions (blue; no change between ancestor and $t_{0}$) and IR-induced deletions (red; decreased coverage at $t_{0}$) shown for ancestor, $t_{0}$ and $t_{1}$. Bottom: Δ coverage ($t_{0}\to t_{1}$) by status, showing a significant increase in IR-induced deletions (Student’s *t* test; $P=4.2e^{-8}$). (**E**) Long reads alignments over the deletion from (A), colored by orientation (red: start, black: end). The *x* axis shows genomic position; vertical lines mark LOH boundaries. Data include real reads (left) and simulated datasets (top and bottom right; see Materials and Methods).

Deletions may arise from NAHR, as described above for the ME experiment, or through alternative DSB repair mechanisms such as NHEJ or single-strand annealing. These pathways typically reseal DNA ends, leaving short deletions often associated with additional sequence alterations at the repair site (*23*, *24*). However, these mechanisms are expected to produce sharp boundaries with an abrupt drop in coverage (coverage change from ∼1.0 to ∼0.5) at the breakpoints of the deletion and a concomitant AF shift (from ∼0.5 to ∼1.0) due to the loss of a parental allele (fig. S4). This pattern is not observed in the deletions induced by IR. Instead, in all 500-Gy–induced deletions, AF progressively increases and stabilizes at ∼1.0, while coverage gradually decreases toward the center of the deletion, reaching a minimum of ∼0.5 to ∼0.85 depending on the size of the deletion (Fig. 5, A and B, and figs. S16 and S17). This pattern suggests interindividual variability in deletion size within each sample. The descendants, coming from a single oocyte of an irradiated mother, reduced the deletion to various extents during the month of culture used for Illumina sequencing. Partial repair explains the gradual decrease in coverage (Fig. 5C, repair only), and partial degradation explains the concomitant AF shifts (Fig. 5C, degradation only). Therefore, the observed pattern points to a process of differential degradation and repair in the culture, representing subclonal heterogeneity as different individuals may carry chromosomes with different extents of degradation and repair at the deletion sites (Fig. 5C, degradation and repair; see also simulations in fig. S18).

To test this idea, the 500-Gy IR clone P500 C18 E3, with the highest number of deletions, was grown for an additional 6 months, and its genome was resequenced using Nanopore Technology. After 6 months, the AF profile of the eight P500 C18 E3 deletions remained essentially the same as before (with a plateau at ∼0.9 instead of ∼1.0 due to the higher error rate of Nanopore sequencing compared to Illumina), while the coverage level continued to increase gradually from the edges of the LOH tracts (fig. S16). In the two smallest deletions, coverage reached a value of ∼1.0, showing a complete recovery of the deleted fragments (Fig. 5B and fig. S16, A and F).

To verify that DNA recovery was specific to IR-induced deletions, the evolution of coverage in the corresponding regions (*N* = 12) was compared to that measured in ancestral deletions (i.e., deletions already present before IR treatment, *N* = 200). While coverage of ancestral deletions remained stable, it increased significantly (Student’s *t* test; $P=4.2e^{-8}$) in IR-induced hemizygous deletions between the Illumina (*t*_0_) and Nanopore (*t*_1_) datasets, consistent with progressive DNA recovery over time (Fig. 5D). The only IR-induced deletions that did not show a temporal increase in coverage were those corresponding to homozygous deletions characterized by a complete loss of coverage at the time of Illumina sequencing. In these cases, DNA repair by HR is impossible because both haplotypes have been lost.

These results are consistent with a dynamic process of deletion lengthening and shortening occurring between breaks on a chromosome through generations. Variable DNA degradation from the initial breakpoints first extends the LOH tract, accounting for the AF shift observed at the deletion boundaries. Over time, transgenerational DNA repair outcompetes DNA degradation by gradually replacing the missing fragment using the homologous chromosome as a template. At the DNA sequence level, this is reflected by the presence of progressive haplotype switches observed in individual Nanopore reads corresponding to the AF shifting regions at the boundaries of deletions (fig. S19). After 6 months of culture of the clone P500 C18 E3, the gain in coverage was within the same range for the different deletions (Fig. 5B). To investigate how AF and coverage profiles evolve under different scenarios of repair and degradation processes, simulations were produced (Supplementary Text and fig. S18). The main result from these simulations is that the observed data are compatible with different scenarios that must involve degradation to generate AF variations and repair that outpaces degradation to have coverage recovery. However, the specific mechanism underlying this coverage recovery remains difficult to disentangle: A bias toward repair can arise from longer repair tracts relative to degradation tracts (fig. S18D), or from a higher probability of repair than degradation at each generation with the same tract length (fig. S18E), or from a more sporadic but complete repair process (fig. S18F). To fit the empirical data, a simplified two-parameter ($\theta_{1}$ and $\theta_{2}$) diffusion-reaction model was used. This yielded similar estimates for $\theta_{1}$ and $\theta_{2}$ across all deletions (fig. S20), indicating a similar rate of coverage recovery and supporting the hypothesis that repair was mediated by the same mechanism.

This mechanism also accounts for the presence of LOH tracts in other samples than P500 C18 E3. In some cases, coverage between the breakpoints of the deletion was only partially recovered, suggesting incomplete repair at the time of Illumina sequencing (figs. S17 and S21). In other cases, coverage was fully restored, indicating full repair before Illumina sequencing (fig. S22, A and C). Some deletions in ME lines also exhibit variability in coverage and AF profiles. Since these LOH events were observed in both hydrated and desiccated lines, the origin of the initial fragmentation remains uncertain, but their presence is consistent with a similar dynamic process of degradation and repair occurring at accidental DNA breaks during population growth. Interpreting the LOH profiles observed in ME lines is further complicated by the fact that bottlenecks introduced during the experiment may have fixed dynamic situations depending on the line and the time of sequencing (fig. S23).

### Transmission of broken chromosomes over generations

The alignments of the long Nanopore reads obtained for the partially repaired chromosomal deletions of the irradiated clone P500 C18 E3 show no intermediates in which distal parts of the deleted fragment are joined, suggesting that the boundaries of the deletion were not resealed during the repair process (Fig. 5E). Supporting this assumption, in silico simulation of 50 resealed intermediates (from Fig. 5A) revealed that reads containing religated DNA sequences should have been detected in the alignment if they were present in the actual dataset (Fig. 5E and fig. S24). This result suggests that IR-induced DNA breakage generated fragmented chromosomes and that the resulting fragments remained unsealed until fully repaired.

**Fig. 6.**
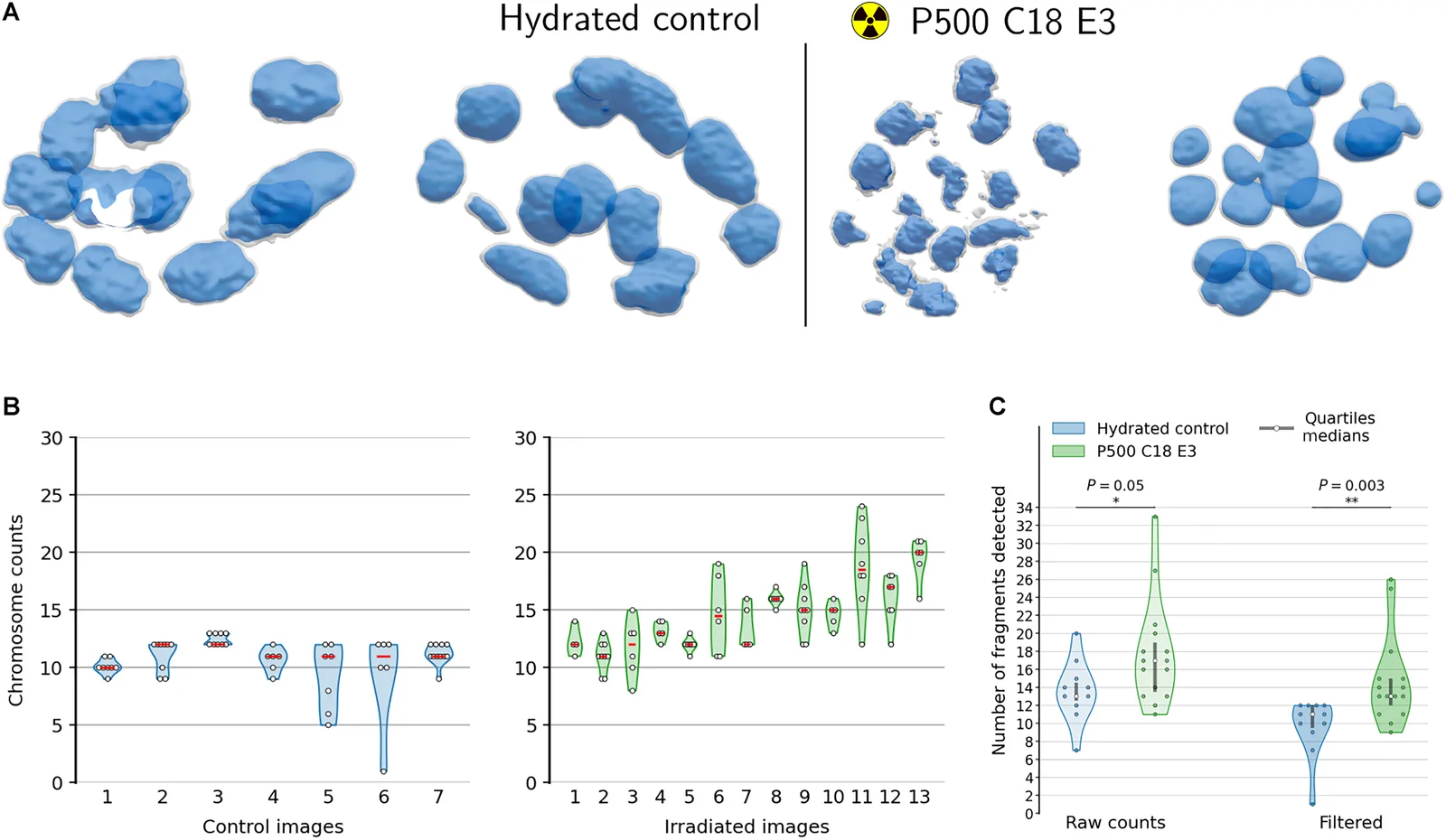
Increased chromosome fragment number in descendants of the *A. vaga* GR 500-Gy IR sample P500 C18 E3. (**A**) 3D z-stack microscopy of one-celled eggs with different DAPI thresholds (blue: stringent and gray: less stringent). Left: nonirradiated controls; karyotypes correspond to control images 2 and 7 in (B). Right: P500 C18 E3 (500-Gy IR); karyotypes correspond to images 10 and 12 in (B). (**B**) Independent chromosome counts from 3D karyotypes. Violin plots show DAPI-stained body counts per image (white dots = blinded observations by humans). Controls (blue, *N* = 7) versus P500 C18 E3 (green, *N* = 13). A linear mixed-effects model (Gaussian, restricted maximum likelihood; image as random intercept; repeated measures nested) shows higher counts in P500 C18 E3 (β = 3.86, *P* = 0.001). (**C**) Automated (computer vision) DAPI body counts. P500 C18 E3 (*N* = 15) shows more fragments than controls (*N* = 11), both before (MWU *P* = 0.05, rank-biserial *r* = 0.461) and after filtering small fragments (MWU *P* = 0.003, rank-biserial *r* = 0.691; see Materials and Methods). Mean fragment number after filtering: 9.73 (control) versus 14.67 (the P500 C18 E3).

To verify the inheritance of broken chromosomes, single-cell eggs from descendants of P500 C18 E3 were arrested in metaphase of the first division, and their karyotypes were compared with those of the laboratory strain (Fig. 6). Eggs collected from the control line displayed an average of 12 4′,6-diamidino-2-phenylindole (DAPI)–stained bodies, consistent with previous karyotyping and chromosome-level genome assemblies (*19*). In contrast, the number of DAPI-stained bodies identified in the P500 C18 E3 progeny is significantly higher (Fig. 6A and table S3) both in human-reported counts (OLS, $P=2.2e^{-12}$; Fig. 6B) and based on computer-vision fragment detection [Mann-Whitney *U* (MWU) = 44.5, *P* = 0.05; Fig. 6C]. These results provide additional evidence that broken chromosomes can be maintained and transmitted over multiple generations in *A. vaga*. The variable karyotype established for different eggs aligns with the genomic heterogeneity of the P500 C18 E3 line, where large chromosomal deletions were either partially or completely repaired over time (Fig. 5B and fig. S16).

## DISCUSSION

Our previous studies established the diploid nature of *A. vaga*, with evidence for meiotic chromosome pairing, recombination signatures, and delayed DNA repair activity in the germ line (*14*, *19*). This study highlights the critical role of meiotic HR in *A. vaga*, both in generating small-scale (GC) and large-scale (CO) LOH and in repairing the genome for faithful transmission to offspring. It reveals a transgenerational mechanism that repairs one-ended DNA breaks and fragmented chromosomes over successive generations.

LOH tracts in hydrated ME lines provide evidence that DSBs, programmed or not, occur frequently in *A. vaga* and induce HR even in the absence of an exogenous source of DNA damage. However, desiccation and IR treatment were found to increase the frequency of HR.

In ME lines, LOH accumulated relatively slowly (on average $3.24e^{-04}$ per bp per generation in hydrated lines) and remained constrained (4.23% of heterozygous sites were lost on average and at most 13.2%), despite experimental conditions that minimize selection. The results support the conclusion that HR takes place during nonreductional meiosis I, when homologous chromosomes pair, and that segregation of recombinants occurs at meiosis II, when half of the sister chromatids are eliminated through the formation of a polar body (*14*). Desiccated ME lines showed a slightly higher accumulation of LOH, likely due to increased DSBs induced by desiccation. However, because desiccation requires culture expansion to ensure survival (*13*), the additional untracked generations introduced during this process may slightly inflate the estimated LOH rate in desiccated lines.

Over multiple generations, intraindividual recombination is expected to increase the homozygosity of *A. vaga* lines. LOH accumulation may theoretically unveil deleterious mutations and reduce overall fitness (*25*). However, experimental evolution studies in highly heterozygous yeasts and theoretical models have shown that LOH can also be beneficial under certain conditions, as it can reveal beneficial mutations and reduce the mutational load (*25*, *26*). Moreover, intraindividual HR can dissociate linked alleles, reducing selective interference and improving the efficiency of selection, particularly in large populations (*27*, *28*).

In our ME lines, LOH accumulated as selection was reduced by bottlenecking each generation to a single individual. In contrast, natural *A. vaga* populations seem to maintain heterozygosity (*17*, *29*, *30*), likely due to stronger selection against LOH in the wild. Nonetheless, some populations exhibit extensive LOH tracts (*17*), and some desiccation-sensitive species like *Rotaria* sp. show very low heterozygosity levels (*29*).

Intraindividual recombination also allows for new allele combinations by swapping portions of homologous chromosomes. In the present study, HR was found to cause copy number and structural variations, including deletions and duplications. *A. vaga* lines exhibited high genomic plasticity, tolerating extensive alterations such as aneuploidy and WGD within the timeframe of a few generations (fig. S10). The inheritance of aneuploidy over generations in *A. vaga* is consistent with a nonreductional meiosis I, as all chromosomes are retained in the oocyte following the first division and transmitted, despite fragmentation.

**Fig. 7.**
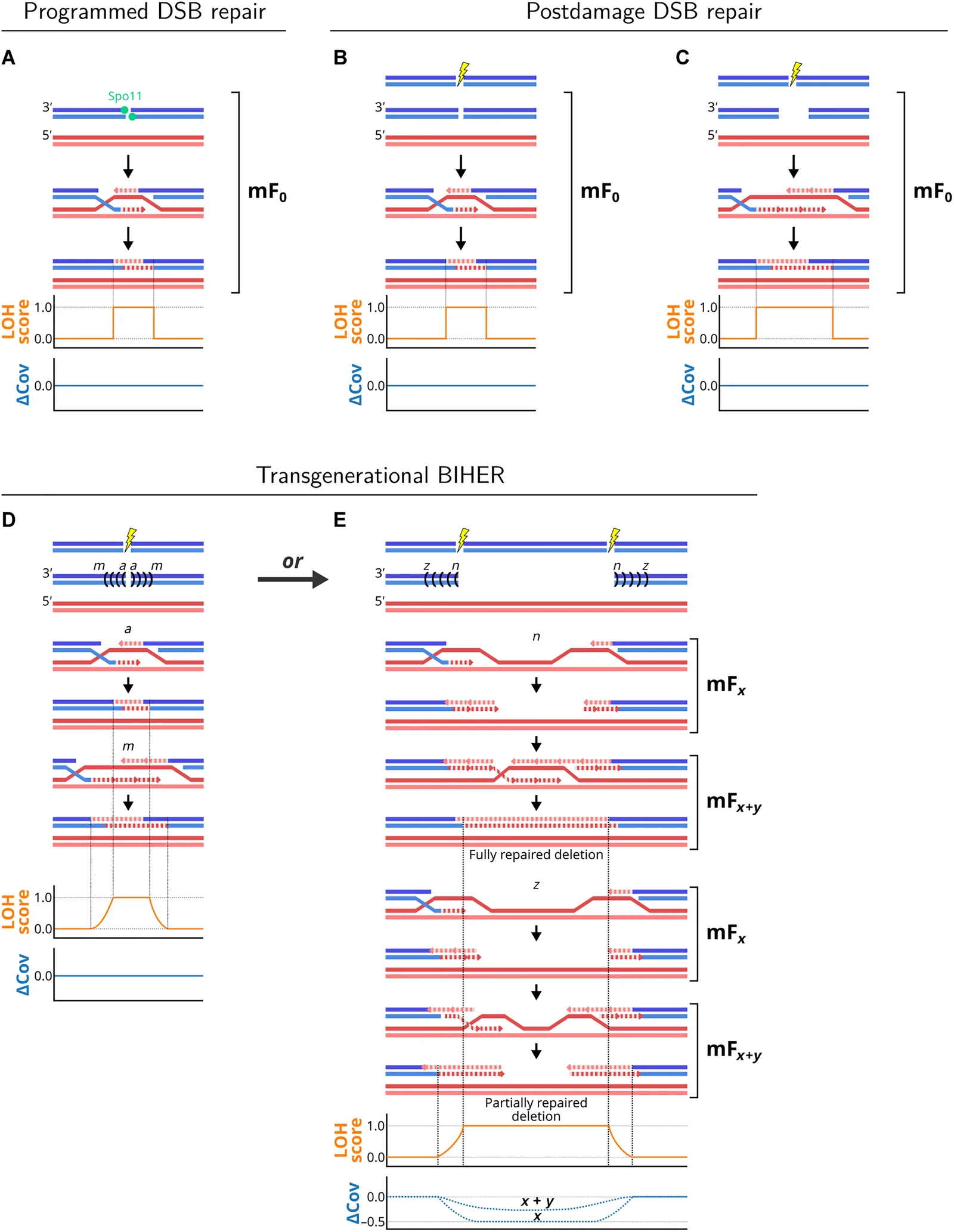
Integrated models for HR and DSB repair in *A. vaga* germ line. The figure illustrates DSB repair during meiosis I in the ancestor (mF_0_) and in offspring after *X* or *X *+ *Y* generations (mF*_X_*, ${\mathrm{mF}}_{X+Y}$). The broken chromosome is shown in blue and the homologous template in red; expected LOH score (orange) and Δ coverage (blue) profiles are shown below each scenario. (**A**) HR repair of Spo11-mediated programmed DSB leading to GC, with or without CO; the LOH tract corresponds to 5′ resection of the broken ends before strand invasion. (**B** and **C**) Accidental DSB (lightning symbol) repaired in mF_0_ (B) without or (C) with further extension of the gap by degradation (brackets), determining LOH tract length. (**D** and **E**) Chromosomes with (D) single or (E) double DSBs transmitted to offspring undergo variable degradation before or during BIHER (e.g., a→m or n→z). LOH score variability at tract boundaries reflects the interindividual differences in deletion length. In short deletions (D), the D-loop initiated at one end reaches the opposite end. These are resolved by a single or few BIHER rounds with no detectable coverage loss, whereas larger deletions (E) require multiple rounds to reduce the deletions completely, leading to a progressive coverage increase across generations.

The conservation of Spo11 in *A. vaga* (*31*) and other bdelloid rotifers (*29*) suggests that repair of programmed DSBs is, at least in part, responsible for the continued LOH accumulation observed in the absence of genotoxic stressors (Fig. 7A). Spo11-triggered HR could also be required to promote chromosome pairing at the time of meiosis, as in sexual organisms (Fig. 7A) (*2*, *32*). We propose that the primary role of homologous chromosome pairing during nonreductional meiosis in *A. vaga* is to enable meiotic recombination to repair accidental DSBs occurring in the germ line and restore genome integrity before passing it to the progeny (Fig. 7, B and C). If they are not repaired immediately, as appears to be the case in the germ line of *A. vaga* (*14*), or if they result from complex DNA damages [such as those caused by IR treatment (*33*, *34*)], some of these lesions may give rise to enlarged breaks due to DNA degradation before homology-directed repair can occur (Fig. 7C). This would explain longer LOH tracts than typically observed in GC events arising from the 5′ end resection of a simple DSB, which usually span less than 5 kb (*35*, *36*) [examples in figs. S7 and S22 (D and F)].

Our results show that broken chromosomes can persist and gradually be reconstructed over multiple generations. This finding points to a new concept of transgenerational DNA repair, which we term break-induced homologous extension repair (BIHER) (Fig. 7). It is inspired by break-induced replication (*37*, *38*), which primarily acts to restart stalled replication forks during vegetative growth and telomere maintenance in certain forms of cancer (*39*). Instead, we propose that BIHER operates specifically between homologous chromosomes paired during the modified meiosis I of *A. vaga*, coinciding with DNA synthesis signatures that were detected in maturing oocytes following DNA damage induction (*14*). BIHER initiates at one-ended DSB, resulting from genome fragmentation and subsequent removal of chromosome fragments (Fig. 7E), or from enlargement of a single DSB by degradation of the surrounding DNA (Fig. 7D). The process then proceeds by strand invasion in the template, followed by DNA synthesis, elongating the broken chromosome. Because elongation is likely of limited processivity, large deletions require multiple rounds of extension before reaching the opposite border of the deletion, with the complementary lagging strand synthesized asynchronously (Fig. 7E). This process occurs at both deletion ends, producing extended chromosome fragments that are inherited through mitosis until they reach meiosis I of a next generation for a new round of BIHER when homologous chromosomes re-pair. Over generations, BIHER-mediated elongation outpaces degradation, leading to a progressive reduction in deletion size and coverage recovery. Eventually, the extended chromosome fragments overlap and may fuse back by canonical HR (Fig. 7E).

Although short deletions could be repaired in a single step by HR (Fig. 7D), some observations in our dataset go against a sporadic, single-step repair acting on larger deletions rather than a progressive process. First, a strictly single-step repair mechanism occurring at different time points during the culture would be expected to produce identical coverage levels across all deletions regardless of size, as each deletion would have the same probability of being either repaired or not. This is not what we observe in Fig. 5B, where the deletions of varying sizes display a gradual, size-dependent recovery of coverage, suggesting a similar repair rate (fig. S20). Second, Δ coverage curves decrease gradually beyond the initial breakpoints, consistent with a stepwise repair process progressing toward the center of the deletion (Fig. 5A and fig. S16) which is particularly obvious after the additional 6 months of culturing.

By acting progressively over multiple generations, BIHER differs from the gap repair mechanism described in *Drosophila*, which uses the sister chromatid to repair chromosomal deletions by undergoing multiple cycles of sequence-dependent strand annealing during somatic development or in the germ line of single F0 individuals (*40*, *41*).

The stable inheritance of fragmented chromosomes through successive cell divisions is difficult to reconcile with classical mechanisms of monocentric chromosome segregation in eukaryotes. Typically, acentric chromosome fragments, lacking the centromere, are rapidly lost as they do not attach to the spindle and cannot separate properly in daughter cells (*42*). Although the exact structure of *A. vaga* centromeres remains to be determined, Hi-C chromosome conformation capture contact maps failed to identify discrete centromeric domains, as is generally the case in other organisms (*19*, *43*). On the basis of the stable inheritance of chromosomal fragments observed in our study, we propose that *A. vaga* chromosomes are holocentric, enabling fragmented chromosomes to segregate independently during cell division (*44*, *45*). Holocentric chromosomes have evolved independently in several plant and animal lineages, where they represent a possible adaptation to frequent chromosome breakage (*46*). It is therefore tempting to speculate that a similar transgenerational repair mechanism, similar to BIHER, could also work in these organisms as an additional contribution to their tolerance against genotoxic stress.

Regardless of the centromeric structure, unrepaired chromosome fragments expose new DNA ends that can be recognized as DNA damage. Their transmission requires partial or total inactivation of the DNA damage checkpoint response to allow cell divisions to proceed despite the presence of DNA breaks (*47*, *48*). This attenuation has been observed in various systems, including cancer cells (*49*), *Saccharomyces cerevisiae* checkpoint mutants (*50*), or clinical isolates of the pathogenic fungus *Candida glabrata* (*51*). However, in these different cases, suppression of the DNA damage response leads to increased genomic instability, with aberrant chromosome rearrangements such as translocations, inversions, and chromothripsis (*52*, *53*). No such genomic instability was detected in *A. vaga*, except for the persistence of chromosome fragments subjected to BIHER to faithfully reconstruct the ancestral karyotype of six pairs of homologous chromosomes.

To prevent end-to-end chromosome fusion by end-joining mechanisms, most eukaryotic cells use telomeric protein-DNA complexes composed of shelterin proteins bound to telomeric repeats synthesized by telomerase (*54*, *55*). However, new telomeres can also form at ectopic chromosome breakpoints, as observed during programmed DNA elimination in ciliates and nematodes (*45*, *56*, *57*) or following the induction of DSBs in yeast, in mice, and, more recently, in humans (*58*–*60*). In *A. vaga*, telomeric repeats were found to be added to truncated chromosome ends following the integration of transposable elements like Athena and related retrotransposons (*61*). DNA sequences corresponding to bdelloid telomeric repeats (fig. S25, A and B) and to Athena fragments (fig. S25, C and D) were detected at deletion breakpoints in a few ME samples (fig. S25, A and B), suggesting that chromosome fragments may be stabilized through neotelomere formation in *A. vaga*. Therefore, it is likely that similar structures could transiently protect unrepaired chromosome ends following DSB induction. Nanopore sequencing of the large chromosomal deletions of the irradiated clone P500 C18 E3 showed more hits matching to Athena sequences than the corresponding regions from a hydrated laboratory culture, while the density of interspersed telomeric repeats showed no meaningful differences (fig. S25, E and F). This pattern is consistent with a transient and dynamic behavior of neotelomeres during the BIHER process, being formed and removed at heterogeneous positions within the repairing chromosomes, leaving only scars in the bulk sequencing data. In contrast, frequent bottlenecks in the ME lines may have randomly fixed individuals with stabilized breakpoints, thus increasing the likelihood of detecting neotelomeric signatures in the corresponding samples (fig. S25, C and D). Our current work aims to study the dynamics of newly formed telomeres during BIHER by examining the fate of targeted DNA breaks with spatial and temporal resolution.

In conclusion, the combination of a nonreductional meiosis I, high genomic plasticity, transgenerational repair via BIHER, and efficient DNA repair proteins (*11*, *15*) offers key insights into the remarkable genomic resilience of *A. vaga* and likely bdelloid rotifers as a whole. More generally, this research highlights how chromosomal features and reproductive strategies may contribute to genome plasticity, fostering diversification and adaptability. We propose that similar mechanisms may operate in other extremo-tolerant species with holocentric chromosomes, such as tardigrades and nematodes. By maintaining reproductive capacity despite chromosomal breakage and enabling progressive, template-mediated restoration across generations, BIHER may represent an adaptation to recurrent DNA damage that enables extremophiles to thrive in harsh environments. This reliance on homology-based repair could also impose selection against chromosomal rearrangements by requiring preserved collinearity for efficient meiotic pairing, thereby contributing to the long-term karyotype stability observed in bdelloids (*62*).

## MATERIALS AND METHODS

### Ancestral strain and cultures

One *A. vaga* individual from the AD008 (cytochrome *c* oxidase subunit I, COI sequence accession number is KM043184) laboratory strain was isolated and cultured until a dense population was established. This population served as the ancestral line for both the ME and GR experiments. All cultures were maintained in SPA(r) water and were fed with sterile (0.45-μm pore-size filtering and further autoclaved for 30 min at 121°C) extract of lettuce juice. Subsequent lineages derived from both experiments were cryopreserved at −80°C and stored in 1.5-ml Eppendorf tubes in SPA water(r).

### ME experimental setup

The experimental design for ME is outlined in fig. S1. Initially, 120 *A. vaga* individuals were randomly selected and isolated from an ancestral culture. These individuals were divided into two batches, each placed across five 12-well plates, with one individual per well. Five plates were assigned to the H (hydrated) condition, remaining continuously hydrated, while the other five plates were allocated to the D (desiccated) condition, undergoing 4 cycles of 10 days of desiccation followed by rehydration. Before each desiccation cycle, the cultures were allowed to grow until they reached ∼1000 individuals to increase the survival rate postdesiccation (*13*).

Every fifth and sixth day (for both conditions when D lines were not in desiccation), plates were inspected for eggs, and one hatchling was randomly selected and isolated to begin the next generation. If the isolated hatchling failed to reproduce, the wells from the previous generations were used as backups, with up to five attempts made before declaring extinction. The number of failed attempts was recorded and considered in the final calculation of the number of generations. To prevent overgrowth of contaminants like bacteria or fungi, the culture medium was refreshed periodically throughout the experiment. Lineages were collected and cryo-stored at two key points: the midpoint, upon reaching isolation number 50 for desiccated and 70 for hydrated lines, and the endpoint, when isolation numbers 88 (desiccated) and 137 (hydrated) were reached. Because of occasional reproduction failures, the actual generation number differed from the isolation number, between 42 and 52, averaging 48 generations for desiccated lines, and between 35 and 69, averaging 64 for hydrated lines at the midpoint, and between 75 and 87, averaging 83 for desiccated lines, and between 117 and 134, averaging 128 generations for hydrated lines at the endpoint. Extinct lines were not counted in these averages. Desiccation was induced at isolation numbers 20, 42, 62, and 82. Table S1 summarizes the actual number of generations reached by each lineage at each isolation step.

### GR experimental setup

The experimental design of GR is summarized in fig. S2. In GR, the ancestral *A. vaga* culture was divided into several treatment groups: (30H) maintained in hydrated conditions for 30 days; (30D) subjected to desiccation for 30 days; (0G) control group, desiccated for 1 day and placed in the irradiation chamber without being irradiated; (100G) desiccated for 1 day and then irradiated with 100-Gy protons; (250G) desiccated 1 day and then irradiated with 250-Gy protons; and (500G) desiccated 1 day and then irradiated with 500-Gy protons. After each experimental treatment, *A. vaga* cultures were rehydrated, and single individual rotifers were immediately isolated in multiwell plates. The third, fourth, fifth, and sixth eggs laid by the isolated mothers were collected and isolated in individual petri dishes. The first two eggs were discarded, since they may have started embryonic development and were not at a unicellular oocyte stage during treatment. The isolated eggs were grown into dense cultures (∼15,000 adults per plate), which were then split into triplicates. The cultures were washed with SPA water and harvested, and the collected rotifer pellets were cryo-stored. The GR sample naming convention was as follows: C## refers to the clone number (i.e., the number of the isolated mother), and E## refers to the egg number (i.e., indicating the order in which the eggs were laid). Some clones share a mother (same C number), but they still constitute distinct replicates because the ovaries already contain a number of pro-oocytes (*14*, *63*). Therefore, the IR treatment is received independently by these individual cells.

### Fertility experiment

The fertility and viability of the cultures were assessed in the GR experiment. The experimental design for the fertility assay is summarized in fig. S11. A total of 120 females were isolated in 12-well culture plates [Greiner bio-one*(r)] and kept at 19°C in 2.0 ml of SPA water for 15 days, with regular feedings of 5 μl of sterile lettuce juice. The number of adult females and laid eggs per well was recorded on days 7 and 15. Wells with fewer than two adults or signs of fungal or bacterial contamination were discarded from the analysis. For wells with over 10 laid eggs, counts were recorded as 10+. Statistical analysis, including two-sided independent *t* tests corrected for unequal variance and Glass’ δ statistics (fig. S12), was conducted using SciPy (*64*).

### Desiccation protocol and irradiation protocol

Desiccation and irradiation of cultures were performed as described in Hespeels *et al.* (*13*). Individuals desiccated for 1 day were submitted to 1.7-MeV proton radiation (delivering 25 keV μm^−1^). For practical reasons, the proton IR treatment required individuals of *A. vaga* to be concentrated on a small desiccated agar patch.

### Culture collection of GR lines

Cultures of each experimental condition were collected in 1.5-ml Eppendorf tubes with SPA water in quadruplicate, with each tube containing approximately 15,000 individuals. Tubes were centrifuged, and the pellets were stored at −80°C. The frozen rotifer pellets were later used for genomic DNA extraction.

### Culture and collection of ME lines for sequencing

ME lines selected for sequencing were fetched from the freezer, and cryopreserved rotifers were thawed at 4°C. Because of the limited number of individuals collected at the midpoint and endpoint of the ME experiment, the thawed rotifers were cultured until the population reached ∼15,000 adults. The cultures were then incubated overnight in 15 ml of SPA water with 150 μl of PenStrep 10,000 U. Cultures were maintained in 75-cm^2^ cell flasks or in 100-mm petri dishes, each containing 15 ml of SPA water. For collection, the petri dishes or cell flasks were vortexed, transferred to 15-ml Falcon tubes, and centrifuged at 3220*g*. The supernatant was discarded, and pellets from tubes containing the same sample were pooled, refilled to 15 ml with filtered SPA water (0.45-μm pore size), and centrifuged again at 3220*g*. Supernatants were discarded, and pellets were washed again with 15 ml of filtered SPA water, centrifuged at 3220*g*, transferred to 1.5-ml Eppendorf tubes, adjusted to 500 μl, and centrifuged at 16,048*g*. The final pellets were cryo-stored for subsequent genomic DNA extraction.

### Genomic DNA extraction

Thawed samples stored at −80°C were brought to 4°C, and the culture pellets were used for DNA extraction with the QIAGEN Gentra Puregene Tissue Kit. Rotifer pellets, each in a 1.5-ml Eppendorf tube, were centrifuged at 16,048*g* for 5 min. The supernatant was discarded, and the pellet was kept on ice for 5 min. Then, 300 μl of lysis solution was added to each pellet and incubated for 15 min, 1.5 μl of proteinase K solution was added, and the pellets were resuspended and incubated overnight at 56°C. The following day, 1.5 μl of ribonuclease A was added, and the solution was gently mixed and incubated for 30 min at 37°C in a shaking dry bath, followed by 2 min on ice. Then, 100 μl of protein precipitation solution was added, and the tubes were vortexed for 20 s. The sample was centrifuged at 16,048*g* for 4 min at 4°C, and the supernatant was transferred into a DNA low-bind Eppendorf tube containing 300 μl of isopropanol. The solution was gently mixed by inversion and centrifuged at 16,048*g* for 2 min at 4°C. The supernatant was discarded, and the pellet was washed with 300 μl of cold ethanol 70% (stored at −20°C), mixed by inversion, and centrifuged again at 16,048*g* for 2 min at 4°C. After discarding the supernatant, the pellets were air-dried at room temperature for ∼10 min, resuspended in 60 μl of DNA hydration solution, and stored at 4°C until use.

### Illumina sequencing

Polymerase chain reaction–free 250-bp paired-end sequencing was performed at the Genomics Core (UZLeuven) on the Illumina HiSeq2000/2500 and NovaSeq platforms, aiming for a sequencing depth of at least 100X per sample.

### Nanopore sequencing

One GR 500-Gy (P500 C18 E3) sample was cultured for an additional 6 months and subsequently sequenced using an Oxford Nanopore PromethION at KeyGene (the Netherlands). Genomic DNA provided to KeyGene was treated with a Circulomics SRE kit to remove smaller fragments. For library construction, 2.5 μg of Ampure purified genomic DNA was used. PromethION 1D Genomic DNA by a ligation SQK-LSK109 library prep kit was used to construct a one-dimensional (1D) library from the sample, and ∼800 ng was loaded onto a FLO-PRO002 (R9.4.1 pore) version flow cell. The sample was sequenced on two flow cells of a PromethION P24 in two 72-hour runs, as the first flow cell had a relatively low pore count. Basecalling was performed in real time on the PromethION compute module using the high-accuracy model from package 20.06.18. A total of ∼123 Gb of Oxford Nanopore Technologies data was generated across the two runs. The sequencing reads that passed quality control (Q≥7) had N50s of 26,140, and 27,035, respectively.

### Quality control of whole-genome sequencing data

Genomic reads were analyzed using FastQC (*65*) and multiQC (*66*). Detailed information on the resulting .fastq files is available in table S1. Some samples exhibited adapter sequence contamination at the read extremities. Other reads had poor quality metrics at the ends. Both adapter sequences and low-quality bases were trimmed before alignment using fastp. In addition, some libraries contained contaminant sequences, detected by the presence of a second %GC peak. Contaminants were mainly bacteria (mostly Enterococcus and *Escherichia coli*) and fungi (mostly *Saccharomycodes ludwigii*) and were retained in the libraries as they would not align to the reference genome.

### Variant calling of Illumina data

The different steps of the variant calling pipeline were performed with a Python wrapper pipeline, runGATK available at https://github.com/AntoineHo/runGATK. First, the Illumina paired-end reads were preprocessed using fastp (*67*) with parameters –detect_adapter_for_pe –trim_poly_g. Then, reads were aligned to the reference genome assembly [AV20 (*19*)] using bwa mem (*68*) with the parameter -K 100000000 and a read group value corresponding to the library. The read group parameter value was set using this pattern: RG

tID:id

tSM:sample

tLB:id_sample

tPL:sequencer

Second, the Binary Alignment/Map (BAM) files were processed using sambamba (*69*) to filter alignments, keeping only those with a mapping quality higher or equal to 20. Then, filtered alignments were sorted and merged for alignments from different libraries of the same sample. Duplicated reads were marked in the merged alignment file. A final BAM file per sample was obtained and used for subsequent analyses.

Third, to obtain a single genomic Variant Call Format (GVCF) file per sample, Genome Analysis Toolkit version 4 (GATK4) HaplotypeCaller was used to call variants with the following parameters: –heterozygosity 0.02 –indel-heterozygosity 0.002 –max-reads-per-alignment-start 0 –output-mode EMIT_ALL_ACTIVE_SITES –pcr-indel-model NONE –emit-ref-confidence BP_RESOLUTION. Last, the GVCF files from all samples (within GR or ME experiments), as well as from the ancestor, were joint-genotyped using GATK4 GenotypeGVCFs with the following parameters: –heterozygosity 0.02 –indel-heterozygosity 0.002 –all-sites.

Two final VCF files were produced containing either only the short variants or all sites (including homozygous references and uncalled sites). For the ME experiment, the joint genotyping was performed a first time, including all time points, and a second time, excluding the midpoint.

### Genotype change detection

The variant file, containing all reference sites, was annotated to provide a comparative analysis between the ancestor and offspring genotypes using a custom Python script. The added information was (i) the number of samples sharing a common variant (added as a format field SH); (ii) the type of variant (added as a format field TP) such as uncalled, biallelic single-nucleotide polymorphism (SNP), biallelic indels, multiallelic SNP, etc.; (iii) and a tag indicating how the site changed compared to the ancestor sample (added as a format field GET) such as C for conserved genotype, L for loss of heterozygosity, N for new heterozygous site. A site was considered multiallelic if more than two alleles accounted for more than 10% of the reads aligning at that position.

### Variant calling of Nanopore data

Nanopore reads were aligned on the reference genome using ngmlr (*70*). The resulting alignments were sorted using samtools (*71*). The coverage was obtained using sambamba depth base (*69*), and variants were called using bcftools mpileup (*71*) and bcftools call. The final VCF file was analyzed with custom Python scripts.

bcftools mpileup was run with the following parameters: –config ont –annotate “FORMAT/DP,FORMAT/AD” –ignore-RG. bcftools call was run with the following parameters: –c -P 0.1 -v.

### AF definition

AF was defined as the ratio between the number of reads supporting the most common allele at a given genomic position to the total number of reads aligned at that position. AF was measured at all sites where heterozygosity was detected in the ancestral sample. AF should approximate 0.5 at heterozygous positions and reach 1.0 when only one allele is present. Sequencing depth at each position was determined using sambamba depth base command (*69*), and both average and median depths per sample were calculated (fig. S26). AF and coverage information of heterozygous sites were parsed from the VCF using pysam. In subsequent analyses, coverage was defined as the total number of reads aligned to a given genomic position divided by the average homozygous coverage in central chromosomal regions of the sample.

### Average heterozygosity calculation

The average heterozygosity was calculated by counting the number of heterozygous sites passing the following filters: 0.35 ≤ AF ≤ 0.65 and 25% ≤ coverage ≤1000% (% coverage being relative to the sample average). The filtered counts, per sample, were then divided by the genome size.

### LOH rate calculation

In addition, in the ME experiment only, the LOH rate was computed as $\frac{1-H}{g}$ where *g* represents the number of generations in the specific ME line under consideration. Average heterozygosity and LOH rates were compared between hydrated and desiccated ME lines at both the midpoint and endpoint of the experiment. Comparisons were also made between endpoint and midpoint values within each condition. Across all pairwise comparisons (e.g., hydrated versus desiccated and midpoint versus endpoint), significance was assessed using a Student’s *t* test without corrections if both samples passed a normality test (Shapiro-Wilk test) and had equal variance (Levene’s test). Student’s *t* tests with correction for unequal variance were performed instead if samples had unequal variances. In samples where normality assumptions failed, an MWU rank test was performed instead. Effect sizes were reported as Cohen’s *d* for Student’s *t* tests and as rank biserial correlation for MWU rank tests.

### LOH cluster definition

Fine-scale events of loss of heterozygosity were detected as clusters of consecutive heterozygous sites in the ancestor that became homozygous in the offspring. The LOH clusters were characterized by three metrics: their genomic position, size (counted in number of ancestrally heterozygous sites), and coverage. The Δ coverage is calculated as follows: $\Delta C=C_{\text{Off}}-C_{\text{anc}}$ where *C* is the average coverage of a LOH cluster (example provided in fig. S4A). The clusters’ positions and boundaries are defined at the variant site. The identified clusters were filtered as follows: (i) the LOH clusters must be unique to individual samples, meaning that clusters shared by more than one sample were excluded to reduce false positives. However, because some analyses included ME midpoint samples, the threshold was sometimes set to two. (ii) Clusters with fewer than five sites were removed. This threshold was chosen on the basis of the results of the independent LOH clustering (see independent LOH clustering). (iii) Clusters with outlier values of coverage were removed (<25% or >1000%, relative to the sample’s average).

For chromosomal-scale representation, clusters separated by less than 1 kb after filtering were merged. This was done for generating figures where many clusters were found in the same genomic region but interspersed with repetitive content. This repetitive content probably caused false positive detection of heterozygous sites, breaking the clusters into smaller chunks.

### LOH tract definition

Chromosome-level events were visualized as LOH tracts with AF and coverage averaged within large, nonoverlapping windows (1/250 of chromosome size). For each individual variant site, a LOH score (defined as $2\times |{\mathrm{AF}}_{\text{Off}}-{\mathrm{AF}}_{\text{anc}}|$) and Δ coverage (defined as the difference between the ancestor and offspring coverage) were computed (fig. S4B).

We excluded that LOH tracts could arise due to recombination between homoeologs/ohnologs because of the large sequence divergence [larger than 15% in conserved regions (*19*)]. This divergence allows mapping the reads on a specific homoeolog (while it does not allow haplotype-specific mapping between homologs with, on average, 1.7% of divergence). Therefore, a conversion event between homoeologs would not produce a LOH tract. Instead, it would be detected as a rearrangement with a duplication signature (local increase of coverage) on one chromosome and a deletion on its homoeologous counterpart (local loss of coverage) that underwent the conversion.

### Windows definition and representations

The values of AF, coverage, LOH score, and Δ coverage were averaged per window and used for chromosome-scale event detection and visualization. Events such as large duplications and deletions were also identified by observing changes in these values. The window sizes for each chromosome were 72,587 bp for chromosome 1, 65,099 bp for chromosome 2, 81,419 bp for chromosome 3, 60,899 bp for chromosome 4, 67,722 bp for chromosome 5, and 55,572 bp for chromosome 6.

Some figures show AF, LOH score, coverage, or Δ coverage variations in local regions. Individual site AF (or LOH score) is always shown without smoothing as semitransparent markers. When a solid line is also present, it corresponds to the rolling average (the window value is centered and each site is weighted uniformly). By default, the window size is 10 sites for AF and LOH scores and 100 bp for coverage, unless a different size is specified on the figure or in its legend.

### Expected variations of LOH score and coverage due to genetic events

Table S2 and fig. S4 summarize the expected patterns of AF, LOH score, coverage, and Δ coverage in various scenarios involving LOH, distinct deletion types, and duplications.

### Independent LOH clustering

Some detected clusters of LOH could actually result from independent losses, such as false negatives or variability in allele sequencing depth, rather than from genetic events such as recombination repair or deletions. We modeled independent losses based on the highest observed LOH frequency (being 0.12 in the desiccated ME sample D5C3). This approach helps determine whether LOH clusters of a given size are likely to arise from random independent losses. We modeled the expected LOH clusters using a binomial distribution (Eqs. 1 and 2). Then, we computed the probability of observing a cluster of any given size (the cluster size is the number of contiguous sites that lost heterozygosity). The results are described in the Supplementary Text and shown in fig. S3

$$p_{\text{LOH}}=N_{\text{LOH}}/N_{\text{Het}}$$ (1)

where $N_{\text{Het}}$ is the number of heterozygous sites in the ancestor sample. $N_{\text{LOH}}$ is the number of lost heterozygous sites in a given offspring sample. $p_{\text{LOH}}$ is the probability for a heterozygous site in the ancestor sample to be lost in a given offspring sample

$$P=\left(\begin{array}{c} n\\ N \end{array}\right)\cdot p^{N}{\left(1-p\right)}^{n-N}$$ (2)

where *P* is the probability distribution of cluster lengths given *p*. *p* is the probability of having a LOH event computed by the number of detected LOH divided by ancestral heterozygosity. *n* is the number of trials. *N* is the number of LOH within *n* trials.

### Simulation of transgenerational degradation and repair

The simulation assumes an initial single genome containing two homologous chromosomes, one broken with a large deletion and the other intact. We only follow 150 heterozygous sites, representing what could be observed with genomic data. The initial deletion ranges from site 50 to 0 (fig. S18). We follow the dynamics of degradation/repair at this deletion boundary around site 50. We iterate several generations (typically 10 to 15). At each generation, the number of chromosomes doubles, but the chromosomes are altered due to degradation and repair. Specifically, the broken chromosome end is degraded with a probability $p_{D}$ over a distance uniformly sampled between 1 and $d_{D}$ sites. The broken chromosome is also repaired with a probability $p_{R}$ over a distance uniformly sampled between 1 and $d_{R}$ sites. We keep track of all chromosomes generated through generations. For instance, at generation 10, we obtain 1024 chromosomes that have a different history of degradation and repair. At each step, we computed the allelic frequency and coverage that would be obtained by sequencing this population at these 150 sites.

This simulation can be used to represent different scenarios of degradation/repair and inform us on the expected pattern in each case. Six scenarios were considered. The parameter values used for each case are described below:

Scenario 1: *p*_D_ = 1 *d*_D_ = 15 *p*_R_ = 0 *d*_R_ = *n*/α

Scenario 2: *p*_D_ = 0 *d*_D_ = *n*/α *p*_R_ = 1 *d*_R_ = 15

Scenario 3: *p*_D_ = 1 *d*_D_ = 15 *p*_R_ = 1 *d*_R_ = 15

Scenario 4: *p*_D_ = 1 *d*_D_ = 15 *p*_R_ = 1 *d*_R_ = 15

Scenario 5: *p*_D_ = 0.25 *d*_D_ = 15 *p*_R_ = 1 *d*_R_ = 15

Scenario 6: *p*_D_ = 1 *d*_D_ = 15 *p*_R_ = 0.25 *d*_R_ = 150

Scenario 1 considers that degradation occurs at each generation, without repair. Scenario 2 considers that repair occurs at each generation, without degradation. Note that the repair is not full at each generation, since the repair tract length is uniformly sampled between 1 and $d_{R}=15$ sites (for a deletion size of 100 sites, it requires, therefore, ∼13 generations, on average, to get a full repair). Scenario 3 considers that repair and degradation occur at each generation and that degradation and repair tract lengths are sampled in the same distribution. Scenarios 4 and 5 introduce a bias toward repair, either by considering that the repair tract length is larger than the degradation tract length (scenario 4) or by assuming that degradation does not occur every generation (scenario 5). Last, in scenario 6, we consider a scenario where repair occurs sporadically (one time every four generations, on average), but when it occurs, it repairs the whole deletion at once. In this case, we do not sample the repair tract length in a uniform distribution but consider that its size is fixed at 150 when repair occurs so that the whole chromosome is repaired, even if it was partially degraded.

### Diffusion model of deletion repair

The deletions appear to be gradually repaired. The molecular detail of this process is not known but results in a smooth fill-up. Heuristically representing this filling process can be achieved using a diffusion-reaction process with only two parameters, $\theta_{1}$, a diffusion rate (that captures the “lateral” smoothing), and $\theta_{2}$, a reaction rate (that captures the vertical filling rate). The net shape of the coverage function at a time *t* depends on the balance of these two rates, given the initial condition (the deletion creates initially a step function in coverage at each of its boundaries). Such a model predicts filling rates that depend on the deletion size (small deletions are repaired quicker since there is less to repair). However, if the same molecular process is acting, irrespective of deletion size, then we could expect to see approximately the same underlying parameter values of $\theta_{1}$ and $\theta_{2}$ for all deletions. Such an observation would indicate a similar net rate of coverage recovery after factoring out the effect of deletion size. To investigate this, we modeled the coverage as a function f(p,t) of position *p* and time *t*. We describe the change in coverage using the reaction-diffusion differential equation (Eq. 3) with initial and boundary conditions (Eq. 4) representing a deletion

$$\begin{array}{c} \frac{\delta f\left(p,t\right)}{\delta t}=\theta_{1}\frac{\delta^{2}f\left(p,t\right)}{\delta p^{2}}+\;\theta_{2}\;f\left(p,t\right)\left[1-f\left(p,t\right)\right] \end{array}$$ (3)

$$\begin{array}{c} a)\;f\left(p,0\right)\;=\;1\;\text{for}\;p\;<p_{\text{min}}\;\text{or}\;p>p_{\max}\\ b)\;f\left(p,0\right)\;=\;1/2\;\text{for}\;p>p_{\text{min}}\;\text{and}\;p<p_{\max}\\ c)\;f\left(0,t\right)\;=\;f\left(1,t\right)\;=\;1 \end{array}$$ (4)

We fitted this model by least squares to coverage data obtained at two different time points (first Illumina sequencing and second Nanopore sequencing) for the different deletions. In Eq. 3, $p_{\text{min}}$ and $p_{\text{max}}$ are the initial boundary positions of the deletions along the chromosome. The coverage data were smoothed using a moving average with a window width of 50 positions before being fitted to the model. The coverage data were also slightly shifted up or down so that their average value was near one at both extremities, as assumed in Eq. 4c. This shift was calculated as one minus the average frequency observed in the windows defined from $\left[0,p_{\text{min}}/2\right]$ to $\left[1/2+p_{\text{max}}/2,1\right]$.

### Deletion and Nanopore reads simulations

samtools faidx was used to extract the reference sequence corresponding to a large deletion observed in sample P500 C18 E3 on chromosome 1. Nanopore long reads were aligned using minimap2 with splice-aware settings (-ax splice -G400k) allowing for large gapped alignments. To analyze the error profile of the real Nanopore dataset, nanosim (*72*) was used, and then two new sets of long reads were simulated on the basis of new reference sequences. The simulated reference sequences were generated by introducing 50 pairs of breakpoints around the actual deletion sites to account for observed variability in deletion size. Two sets of long reads were produced per deletion. The first set contained the deletions ligated in silico (set 1). In the other set, the deletions were left unligated (set 2). In addition, both sets also contained 50 undeleted reference sequences to simulate the presence of a nondeleted allele. The long reads of both sets were aligned with minimap2 using the same splice-aware settings. A custom Python script was used to plot the alignments obtained with the real dataset and both simulated sets (unligated versus ligated deletions). A summary of this methodology is presented in fig. S24.

### Telomeric sequence and Athena detection

The alignment files of ME lines presenting deletions with clearly delimited breakpoints were filtered with samtools view to extract only reads aligning around the breakpoints. Then, these subsets of reads were visualized using Integrative Genomics Viewer (IGV). When the regions contained short reads with soft clipping, the clipped sequences were extracted using pysam and scanned for the bdelloid telomeric motif (TGTGGG)*n*. If the telomeric motif was not retrieved, then the clipped sequences were assembled (when possible) and realigned on the reference using minimap2 to detect rearrangements. In addition, these sequences were blasted against the National Center for Biotechnology Information (NCBI) “nt” database. In one case, we found hard-clipped reads for which the supplementary alignment coordinates were retrieved using IGV.

The P500 C18 E3 Nanopore sequencing was compared to a hydrated culture Nanopore sequencing “BXQ F” accession: SRR13348928 (*19*). For both libraries, individual Nanopore reads that aligned (detailed in the section about variant calling of Nanopore reads) in the defined deleted regions were screened with pysam, clipped sequences were extracted, and the proportion of bdelloid telomeric motifs was measured. The clipped sequences were also aligned against the Athena reference sequence (accession EU643487.1) using minimap2 Python bindings (mappy). Hits were defined as sequences with partial matches of at least 100 bp with a minimum of 50% identity.

### Karyotypes

A clonal culture was initiated using a rotifer back-up from the GR sample P500 C18 E3 and maintained in a 75-cm^2^ cell flask. The stock P500 C18 E3 culture was then maintained over 6 weeks, during which additional generations occurred. The karyotyping described below was performed several times over this period. When the population reached ∼40,000 individuals, a clean laboratory strain culture was similarly maintained and used as a control. The individuals were then concentrated in a smaller volume (10-cm^2^ petri dish) and cultured for 2 days at 25°C in nutrient-rich water supplemented with demecolcine (10 μg/ml) to stop egg development during the first metaphase. After 2 days, eggs were collected in a 1.5-ml Eppendorf tube and centrifuged at 7379*g*. The supernatant was discarded, and 500 μl of SPA water (put at pH 10 with NaOH) was added. Then, 500 μl of a diluted bleach solution (14 ml of SPA water with 1 ml of bleach) was added. The tubes were vortexed, centrifuged again at 7379*g* for 2 min, and rinsed with 1 ml of SPA water, and the supernatant was removed. These washing steps were repeated three times. The egg pellet was resuspended in 1 ml of a hypotonic solution (0.05 M sucrose in distilled water) and transferred to a 15-ml Falcon tube. While the tube was vortexed at high speed, 4 ml of the hypotonic solution was slowly added drop by drop. The eggs were incubated overnight on an agitator. The tubes were then centrifuged for 10 min at 3220*g*, and the supernatant was removed. Next, 5 ml of a fixative solution (three parts methanol to one part acetic acid) was slowly added drop by drop, while the tubes were vortexed at high speed. The tubes were left on ice for 1 hour and then centrifuged at 3220*g* for 10 min. The supernatant was discarded, and 5 ml of the fixative solution was added drop by drop, while the tubes were vortexed at high speed. The tubes were left on ice for 20 min and centrifuged at 3220*g* for 10 min. The supernatant was discarded. Approximately 15 μl of the fixed eggs was dropped from about 15 cm onto a microscope slide. The drops were air-dried and, for some slides, gently squashed by pressing on the coverslip. The samples were then stained with DAPI [1 μg/ml in phosphate-buffered saline (PBS)] and washed twice with PBS. The slides were mounted with fluoromount-G, allowed to solidify for at least 1 hour at 37°C) and stored at 4°C until observation. Z-stack images of one-cell-stage eggs were then acquired using a Zeiss LSM Airyscan confocal microscope. Images were taken only when chromosomes were clearly visible and when at least 10 chromosomes were initially counted.

The Z-stack images were thresholded and converted to 3D models using a custom Python script. These 3D images were uploaded to a public website, where users could input chromosome counts. To maintain objectivity, the experimental condition was masked to the users, and some images were duplicated to check consistency in counting. Final counts were aggregated, and noisy images with high variability in counts were discarded from the analysis.

The 3D multichannel Tagged Image File Format z-stacks were imported using the tifffile library and preprocessed to enhance contrast and reduce background. Background was further reduced using white top-hat filtering when specified. Each individual 2D slice of the z-stack was segmented into candidate objects using a guided multilevel Otsu thresholding strategy, which optionally incorporated the segmentation mask of the previous slice to stabilize thresholding across the *z* dimension. The resulting thresholded images were used to create binary masks. Fragment boundaries were optionally refined using a watershed transform or random walker segmentation (empirically tested on each image to determine which algorithm resulted in the best segmentation). Labeled regions smaller than a defined area threshold (100 pixels) were discarded. Objects were tracked across adjacent z-slices by computing pairwise centroid distances. Objects spanning fewer than five slices were excluded from downstream analyses to reduce noise from spurious detections. For each retained object track, a 3D binary volume was reconstructed by stacking the segmented 2D regions across slices (an example of the segmentation result is presented in fig. S27). Shape features were extracted from each 3D object, including total volume (voxel count), surface area (via marching cubes), and surface-to-volume ratio. Fluorescence intensity–based features were also computed, including mean voxel intensity, total intensity, and intensity-to-volume ratio. Filtered counts correspond to the number of fragments after removing those below arbitrary thresholds for these measurements. The number of images differs in the human counts method and the segmentation method because the images that did not have more than three individual human counts reported were not used in the analysis.

## Supplementary Materials

### The PDF file includes:

Supplementary Text Figs. S1 to S27 Legends for tables S1 to S3 References
