# Supplementary material for: Transgenerational chromosome repair in the asexual bdelloid rotifer Adineta vaga
Source: Sci Adv. 2026 Sep 11;12(37):eaee0891. doi: 10.1126/sciadv.aee0891 (PMC13564844; doi:10.1126/sciadv.aee0891)
Supplement: Supplementary file 1 [file sciadv.aee0891_sm.pdf]

Science Advances
AAAS

Supplementary Materials for

Transgenerational chromosome repair in the asexual bdelloid rotifer *Adineta vaga*

Antoine Houtain *et al.*

Corresponding author: Antoine Houtain, antoine.houtain@gmail.com;
Bernard Hallet, bernard.hallet@uclouvain.be; Karine Van Doninck, karine.van.doninck@ulb.be

Sci. Adv. **12**, eaee0891 (2026)
DOI: 10.1126/sciadv.aee0891

The PDF file includes:

Supplementary Text
Figs. S1 to S27
Legends for tables S1 to S3
References

Other Supplementary Material for this manuscript includes the following:

Tables S1 to S3

Supplementary Text

Coverage and clone effect do not significantly impact LOH in the GR experiment

In the GR experiment, LOH could arise from other factors than treatment. Two potential confounding factors are coverage variability between samples and shared maternal origin of isolated individuals (i.e., different eggs were obtained from the same mother after treatment). To determine whether these confounding factors impacted LOH, we used linear mixed-effects model analysis with the log-transformed LOH as the dependent variable. Treatment groups included 0 Gy, 30D, 30H, 100 Gy, 250 Gy, and 500 Gy. The coverage was included as a covariate. Clonal identity (shared mother) was used as a random effect to account for the shared ancestry between some cultures. The data used and model results are presented in table S3.

The full model (Model 1) showed that the treatment with 500 Gy significantly increased LOH compared to the reference (0 Gy) ($\beta = 0.332$, $p = 0.001$). No significant effects were observed for other treatments: 100 Gy ($\beta = 0.072$, $p = 0.61$), 250 Gy ($\beta = 0.043$, $p = 0.742$), 30D ($\beta = 0.024$, $p = 0.816$), and 30H ($\beta = -0.018$, $p = 0.865$). Coverage did not significantly influence LOH ($\beta = 0$, $p = 0.753$), and the shared ancestry had a small effect (coefficient of 0.015). In a model excluding the treatment (Model 2), coverage remained non-significant ($\beta = 0$, $p = 0.826$), suggesting that the variability in coverage does not significantly account for changes in LOH.

To assess the robustness of the observed effects, we performed an OLS regression without the random effect of clone (Model 3). The significant effect of the 500 Gy treatment remained ($\beta = 0.2065$, $p = 0.012$), while the effects for 100 Gy ($p = 0.09$) and 250 Gy ($p = 0.096$) were marginal. The effect of coverage was significant in this model ($\beta = 0.0017$, $p = 0.016$), indicating a potential minor influence on LOH when not accounting for random effects.

Taken together, these results indicate that the GR cultures ($N = 4$) obtained from eggs of mothers that were exposed to high doses of radiation (500 Gy) have significantly more LOH compared to the control (0 Gy) and lower doses of IR that do not have a statistically significant effect (fig. S13). Moreover, the inclusion of the shared ancestry as a random effect does not significantly alter the estimated treatment effects and does not improve model fitting. Therefore, sharing the same mother does not substantially impact the probability of having more or less LOH, as expected given that

IR impacts cells that were already differentiated in the adult mothers. Although coverage appears statistically significant in a simple OLS model (Model 3, $p = 0$), this effect is small (coefficient of 0.0017) and positive (contrary to the expectation that lower coverage could be linked to more false positive LOH). Overall, this suggests that the impact of coverage on LOH detection is negligible.

Gradual variations in a genomic region are not due to technical bias and reference content

We observed several cases of gradual allele frequency (AF and LOH score) shifts that are sometimes associated with gradual coverage shifts (Δ coverage). These gradual shifts are retrieved at all doses: 100, 250, and 500 Gy and affect regions spanning between a few Kb up to several Mb (figs. S16, S17 S22D-F, and S21). One 500 Gy sample (P500 C18 E3) has more deletions than all the rest and was re-sequenced after an additional culture time using Nanopore.

Gradual shifting of AF was also observed in some ME samples, in which they are always concomitant with gradual shifts of coverage (fig. S23). In ME, these are prevalent at chromosome extremities where they probably correspond to deletions (fig. S23A,C). The coverage shifts usually occur over regions much larger than the Illumina insert size (> 2 Kbps), with a few exceptions where the shift is abrupt and likely corresponds to a breakpoint (fig. S23B that is also represented in figure S25A-C).

The gradual changes (spanning more than a kilobase) could be due to (1) technical bias (e.g., different mapping tools and parameters when comparing Illumina and Nanopore sequencing data in the P500 C18 E3); (2) local reference content (e.g., higher repetitive and transposable elements content, preventing unique mapping); and (3) 'sub-clonal' or 'inter-individual' variability due to a mix of genotypes found in the culture and represented with different proportions in the pool sequencing of several thousand individuals.

While technical biases (1) can arise when methodologies are different, they do not account for the observations of gradual shifts or breakpoints across samples when the methodology is identical. Similarly, if local reference content (2) produced gradual changes, then similar AF and coverage curves should be observed in the same regions across samples and timepoints. In two distinct ME lines (samples H2A3 and D5B3), the same chromosome region acquired a deletion, but the two lines have different coverage patterns; one is gradual (fig. S23A) and the other is an abrupt shift (fig. S23B). In another ME line (sample D4B4), the midpoint sequencing shows a gradual coverage

transition over 180 Kb, while the endpoint sequencing of the same line appears to have a stable breakpoint (fig. S23C). In both cases, the DNA extraction, library preparation, sequencing, and bioinformatics analyses were performed with identical protocols. Therefore, these results rule out a technical bias due to different methodology or an effect of the local reference content.

In conclusion, gradual shifts in both coverage and AF are best explained by inter-individual variability (3). By contrast, the abrupt shifts observed result from bottlenecking in ME that selects a single genotype from the population. This is consistent with the absence of clear breakpoints in GR, whereas a few examples were detected in ME.

Distribution of large LOH clusters' positions in ME and GR

To test whether the distribution of large LOH clusters (> 100 sites) differs between the ME lines and GR 500 Gy IR conditions, the positional distribution of events along the normalized axis (relative position along any chromosome) was compared using both cumulative density and binned frequency approaches.

The cumulative distribution of positions in both sets revealed significant differences in the spatial distribution of LOH clusters (Kolmogorov-Smirnov test, $KS = 0.254$, $p = 5.33e^{-5}$; fig. S15A). The ME lines displayed a steeper increase in the extremities, and fewer events occurred in the core regions (flatter curve between positions 0.3 and 0.6). In contrast, the GR 500 Gy IR distribution follows more closely the diagonal (steeper curve between positions 0.4 and 0.6).

The large LOH clusters (> 100 sites) were binned (among 18 intervals) by their leftmost positions (fig. S15B). The ME lines clusters appear enriched at the chromosome extremities, where the observed counts significantly exceed the expected values, consistent with a distribution more skewed towards the edges. Conversely, the GR 500 Gy IR condition shows a more uniform distribution of clusters across the bins, with observed counts aligning closely with the expected values. A binned chi-squared test confirms that both conditions are significantly different ($\chi^2 = 268.53$, $p = 3.36e^{-47}$).

Because the null hypothesis is that both distributions of ME and GR 500 Gy are the same, the expected distribution (under the null hypothesis) is not uniform. The expected values were obtained using the χ^2 contingency method, assuming that both distributions are identical in each bin:

$$E_{\text{bin}}(\text{ME}) = \frac{O_{\text{total}}(\text{ME}) \times O_{\text{bin}}(\text{GR} + \text{ME})}{O_{\text{total}}(\text{GR} + \text{ME})})$$

$$E_{\text{bin}}(\text{GR}) = \frac{O_{\text{total}}(\text{GR}) \times O_{\text{bin}}(\text{GR} + \text{ME})}{O_{\text{total}}(\text{GR} + \text{ME})})$$

Where E_{bin} is the expected number of LOH tracts in a bin, and O_{bin} is the observed number of events in a bin. Total indicates the total number of events.

The ME distribution is consistent with LOH clusters produced by spontaneous recombination in a non-reductional meiosis I. Conversely, the absence of bias towards extremities in the GR 500 Gy samples probably reflects HR repair of stochastic damage caused by the IR treatment, which should not affect specific regions.

Detection of telomeric motifs and Athena retrotransposon sequence at chromosome breakage sites

Different coverage shifts from 1.0 to 0.5 or from 0.5 to 0.0 were detected along chromosomes of several ME lines. While some of them are gradual (shifting over more than 1 kb), others present with abrupt changes spanning between 100 bp and 1 Kb. These correspond to chromosome breakpoints and were not found in the GR samples of IR-induced deletions.

These breakpoints likely arose following chromosome fragmentation. Such fragments could be stabilized in different ways. The newly formed DNA ends can be recognized as DNA damage and ligated by end-joining mechanisms. In these instances, end-joining can create rearrangements or hemizygous deletions, depending on the ends involved. Another possibility is the addition of telomeric motifs that will protect the newly formed fragments from the end-joining machinery. These fragments would then be transmitted as normal chromosomes. In bdelloids, it was shown that a family of retroelements, the Penelope-like elements (PLEs) are associated with telomerase activity and use the telomeric motifs to prime their reverse transcription (*61*).

To determine how the deletion breakpoints found in ME are stabilized, we inspected the short reads alignments at these breakpoints. In several instances, we found the bdelloid telomeric motifs, TGTGGGn (fig. S25A,B). On one occasion, we observed a rearrangement, with a portion of the H4A4 chromosome 6 presenting a breakpoint at which paired-end reads clipping indicated that they were aligning to a portion of chromosome 4 where we found a duplicated region. Finally, in

the case of the large deletion on Chromosome 6 of the ME line H2C3. A sequence blasting to the Athena PLE was found in soft-clipped reads at both breakpoints of the deletion (fig. S25C,D). Interestingly, the vast majority of reads were either paired and aligned or soft-clipped, but only 4 reads presented hard-clipped sequences that aligned to another chromosome (compared to 550 reads with soft-clipped sequences over a total of 6797 reads aligning around the deletion breakpoints). The presence of telomeric motifs and Athena PLEs at the few breakpoints in ME samples indicates that chromosome ends can be protected by stabilizing sequences. While the absence of hard-clipped sequences supports the hypothesis that chromosomes are fragmented rather than rearranged, it remains unclear whether rearrangements occur through end-joining and, if they do, at what frequency. Moreover, the short reads' extent will fail to capture rearrangement if there is a long sequence insertion at the breakpoint (longer than a read insert size), as could be the case in line H2C3, where we found a sequence aligning to Athena on both sides of a long deletion, but we cannot test whether these are ligated together or separate.

Detecting telomeric motifs in IR-induced deletions

To determine whether chromosome fragments were also stabilized after IR treatment in GR samples, the Nanopore reads resequencing of the sample P500 C18 E3 were scanned for Athena and the bdelloid telomeric motifs. In the large IR-induced deleted regions, some Nanopore long reads have clipped sequences that align to Athena. However, this represents a tiny fraction of sequences relative to the expected 1000X sequencing depth (at most 13 hits in a window of 5 Kb). To compare with a similar dataset, we used 125X of Nanopore sequencing (BXQ F obtained from a hydrated culture of the same lab strain of *A. vaga*, publicly available on NCBI (*19*)). Depending on the deletion, there were either no hits to Athena or fewer, but some are still retrieved (fig. S25E). Similarly, the density of telomeric motifs does not appear to be higher in P500 C18 E3 compared to BXQ F. While several windows reached statistical significance due to the large sample sizes (Mann-Whitney U $p < 0.001$), the effect sizes were uniformly small (rank-biserial $r < 0.25$) and the median differences were minor (typically < 0.05), indicating no meaningful difference between groups (fig. S25F). In sum, we could not detect convincing patterns that indicate the addition of telomeric motifs after IR treatment, but we could not rule out that it does not happen either.

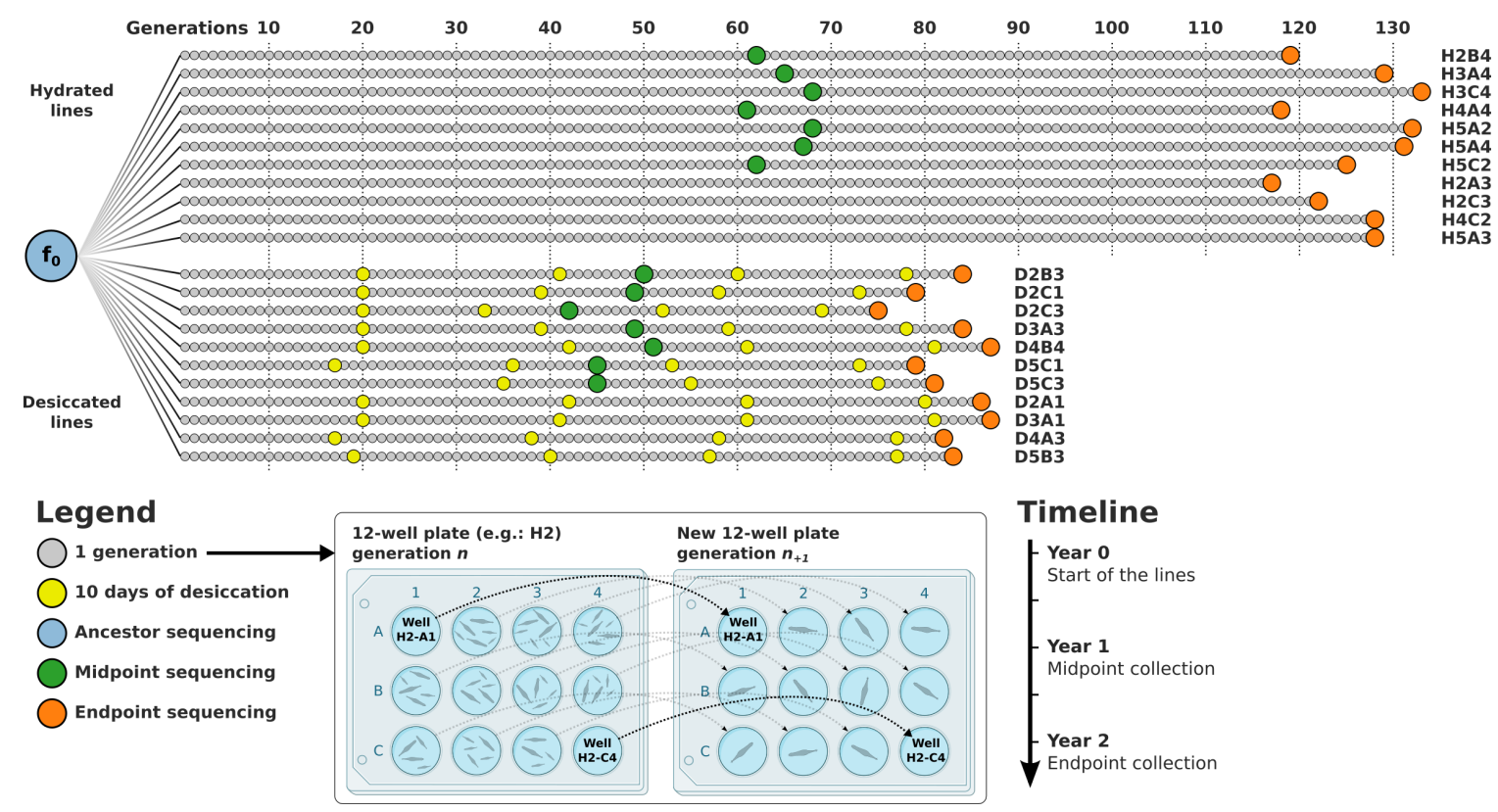

Figure S1: **Summary of the experimental design of the micro evolution (ME) experiment.** Starting from the same ancestral culture (f_0), 22 *A. vaga* lineages were experiencing bottlenecks at each generation (represented by a grey circle), following the design of a mutation accumulation experiment (*21, 73*). The 22 lines were sequenced using Illumina 2x250 bp PE after 88 (desiccated lines) and 137 isolations (hydrated lines). One isolation corresponds to a generation since the start. Because some isolated individuals failed to reproduce, the actual number of generations varies between lines. The last isolation of a line is referred to as the experiment's endpoint (represented by orange circles). In addition, 14 lines were sequenced after one year, corresponding to isolation 70 (hydrated lines) and 52 (desiccated lines). The sequences corresponding to these isolations are referred to as midpoints (represented by green circles). Hydrated lines were kept in permanent hydrated conditions. Desiccated lines underwent four desiccation events of 10 days (represented by yellow circles) every 20 isolations.

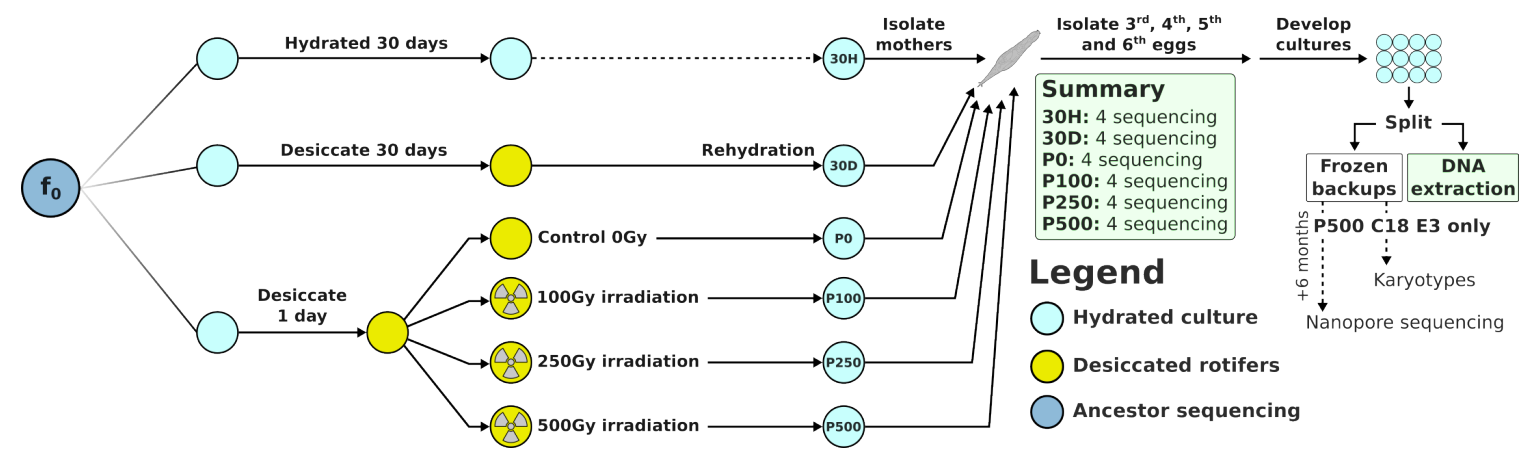

Figure S2: **Summary of the experimental design of the genome repair (GR) experiment.** All cultures originated from the same ancestral laboratory strain (f_0). Cultures were divided into experimental treatments: one month under hydrated conditions served as the control (30H), one month under desiccated conditions (30D), and exposure to proton irradiation at doses of 0 (control P0), 100 (P100), 250 (P250) and 500 (P500) Gy. Individual mothers were isolated from the treated cultures, and the third, fourth, fifth, and sixth eggs laid by those mothers were collected individually to initiate new clonal cultures (one-egg bottleneck). Four cultures from each treatment were sequenced in Illumina, producing 2x250 bp PE libraries. One of the four samples sequenced from the P500 series (P500 C18 E3) was used to generate karyotypes of single-celled eggs and was re-sequenced using Nanopore technology after an additional 6 months of culture.

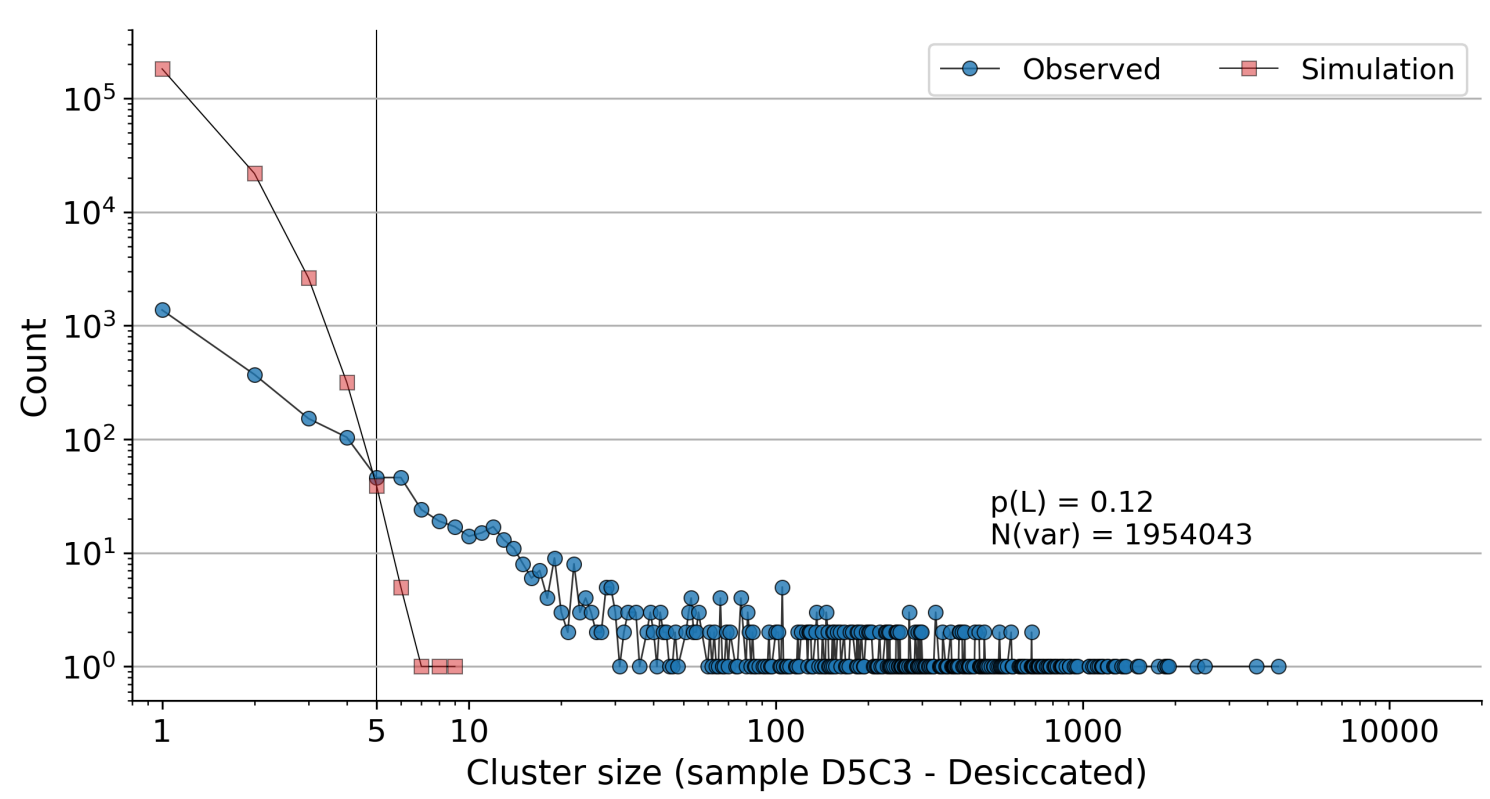

Figure S3: **LOH cluster size frequency count in ME sample D5C3.** LOH cluster size (measured by the number of sites lost) compared between observed data and simulated data. The simulated data was produced using a binomial distribution with $p_{LOH} = N_{Hom}/N_{Het}$; N_{Hom} and N_{Het} are the number of detected heterozygous sites that were, respectively, lost and conserved compared to the ancestor. The sample with the highest N_{Hom}/N_{Het} was chosen (ME sample D5C3) to perform this analysis.

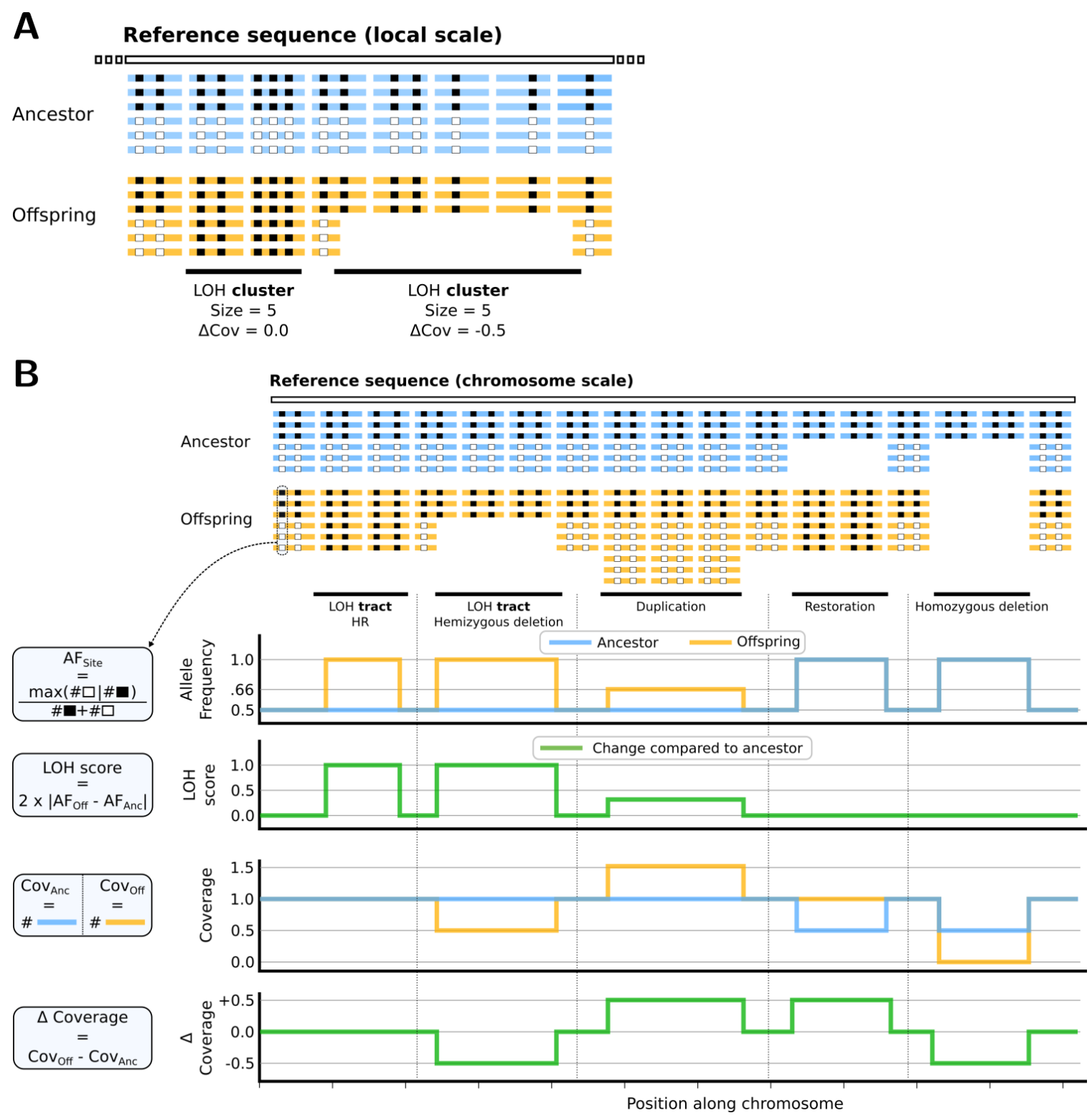

Figure S4: **Detection of LOH clusters and detection and scoring of LOH tracts and CNVs based on alignment and calling data.** (**A**) Expected signatures of LOH clusters resulting from homologous recombination (HR) or deletions. (**B**) Detection of LOH tracts from HR, hemizygous and homozygous deletions, as well as duplications and restorations based on expected patterns of allele frequency (AF), LOH score, coverage, and Δ coverage.

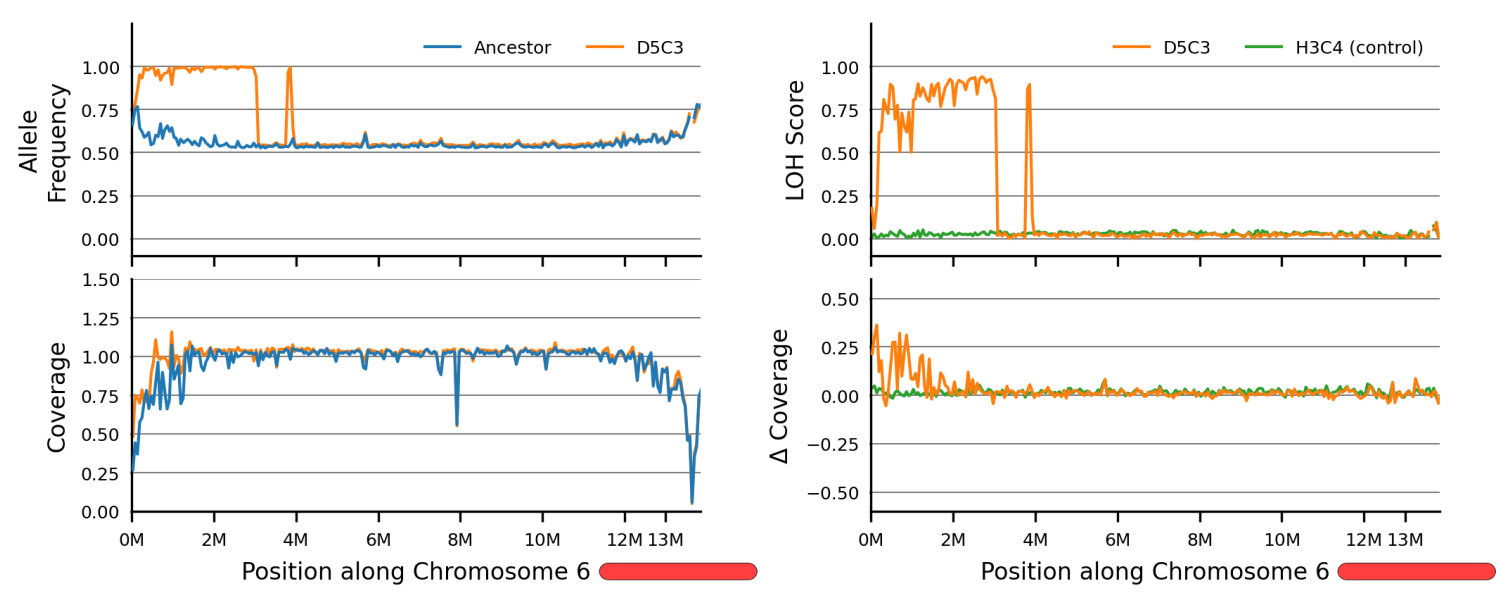

Figure S5: **Illustrative comparison of allele frequency (AF), coverage, LOH score and Δ coverage.** (**Left**) Allele frequency (AF, upper panel) and coverage (lower panel) along chromosome 6 in the ancestor compared to ME sample D5C3. (**Right**) LOH score (upper panel) and Δ coverage (lower panel) along chromosome 6 in ME samples D5C3 and H3C4 (H3C4 is used as a negative control without LOH on this chromosome). The red highlights on the cartoon chromosomes indicate where the depicted regions are located along the corresponding chromosome.

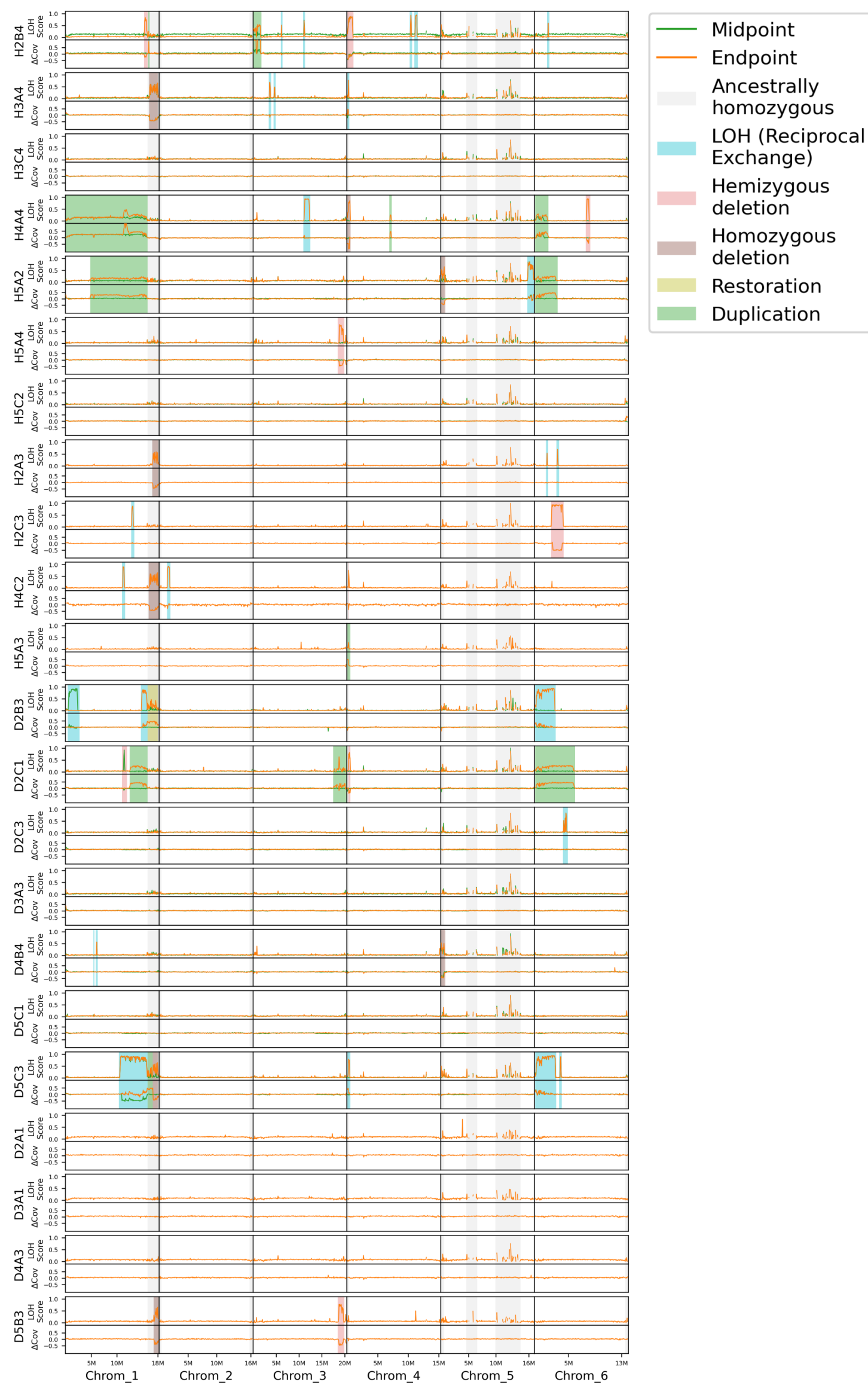

Figure S6: **Overview of LOH score and Δ coverage across all chromosomes and ME samples.** LOH score and Δ coverage across all chromosomes in ME samples with large events (> 50 Kb) highlighted in color. Events include HR and reciprocal exchange (blue), hemizygous deletions (light red), homozygous deletions (dark red), restorations (yellow), and duplications (green). Ancestrally homozygous regions are also represented (grey). Values are averaged over 250 non-overlapping windows along each chromosome, with window sizes ranging from 55.6 Kb (Chromosome 6) to 81.4 Kb (Chromosome 3). The window size is defined as 1/251 of the chromosome length. Lines starting with H correspond to permanently hydrated lines, whereas lines starting with D correspond to lines that underwent four events of 10 days of desiccation.

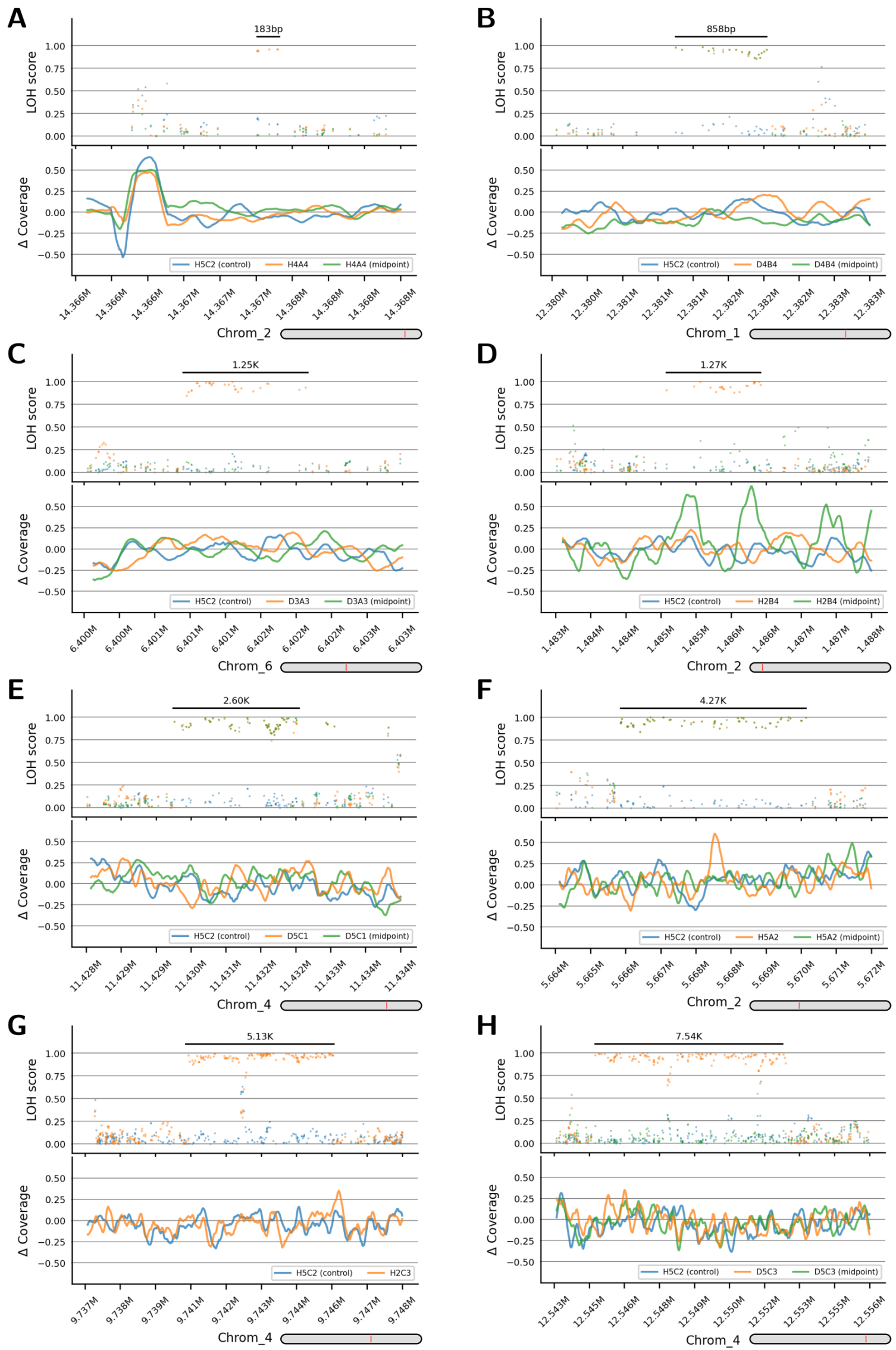

Figure S7: **Examples of short (< 8 Kb) LOH tracts detected in the ME experiment.** (**A-H**) LOH score (upper panel) and Δ coverage (lower panel) for 8 examples of short LOH tracts (< 8 Kb) across different lines, conditions, and chromosomes detected in ME samples. The red highlight on the cartoon chromosome (bottom right of each subfigure) indicates where the depicted regions are located along the corresponding chromosome.

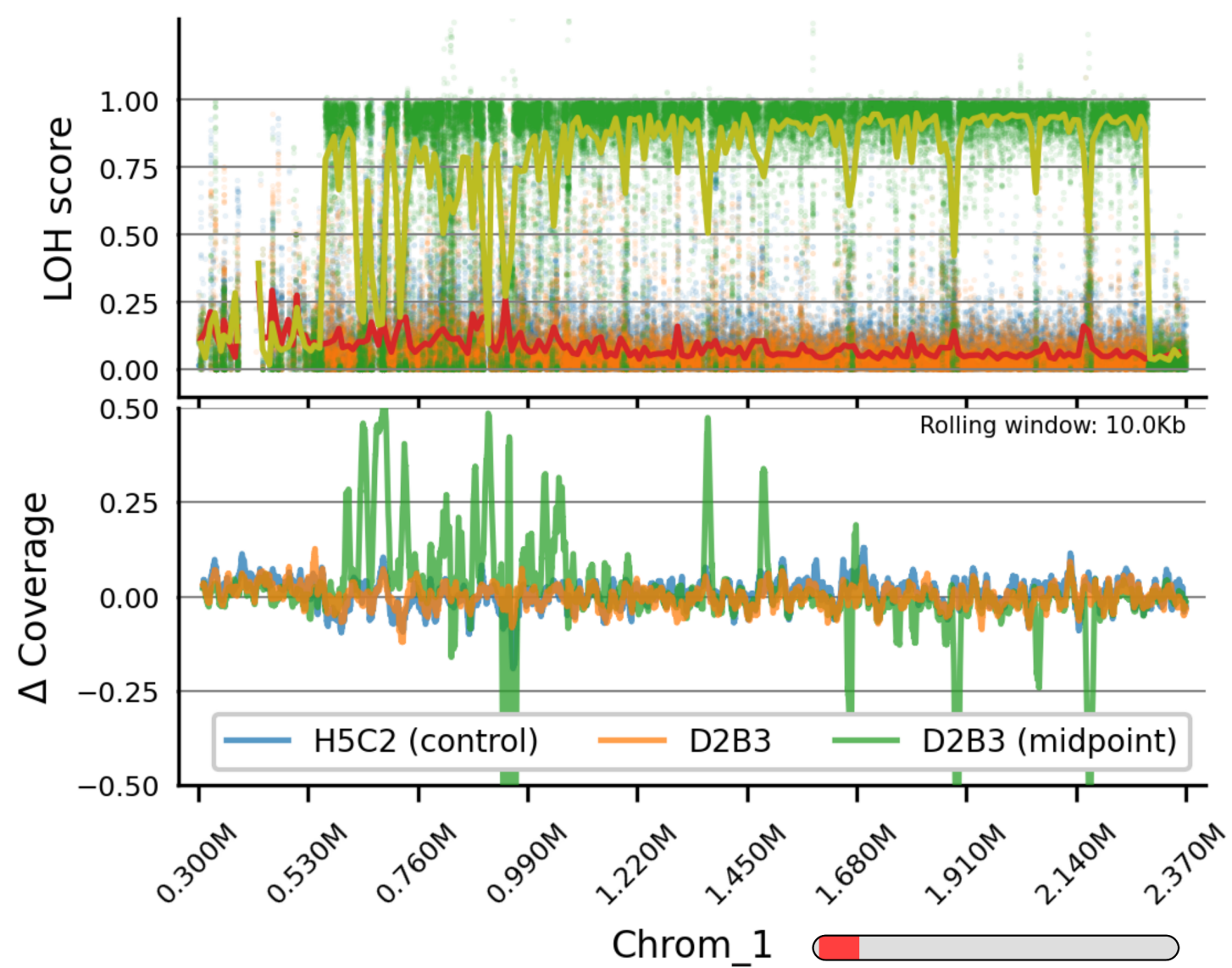

Figure S8: **LOH score and Δ coverage along a portion of chromosome 1 of ME sample D2B3.** LOH score (upper panel) and Δ coverage (lower panel) across chromosome 1, region 0.3 Mb to 2.37 Mb. Midpoint sequencing of ME sample D2B3 (green) compared its endpoint (orange). A negative control without LOH is shown in blue (from line H5C2). Lines correspond to smoothed averages, and dots represent SNPs. The red highlight on the cartoon chromosome indicates where the depicted regions are located along the corresponding chromosome.

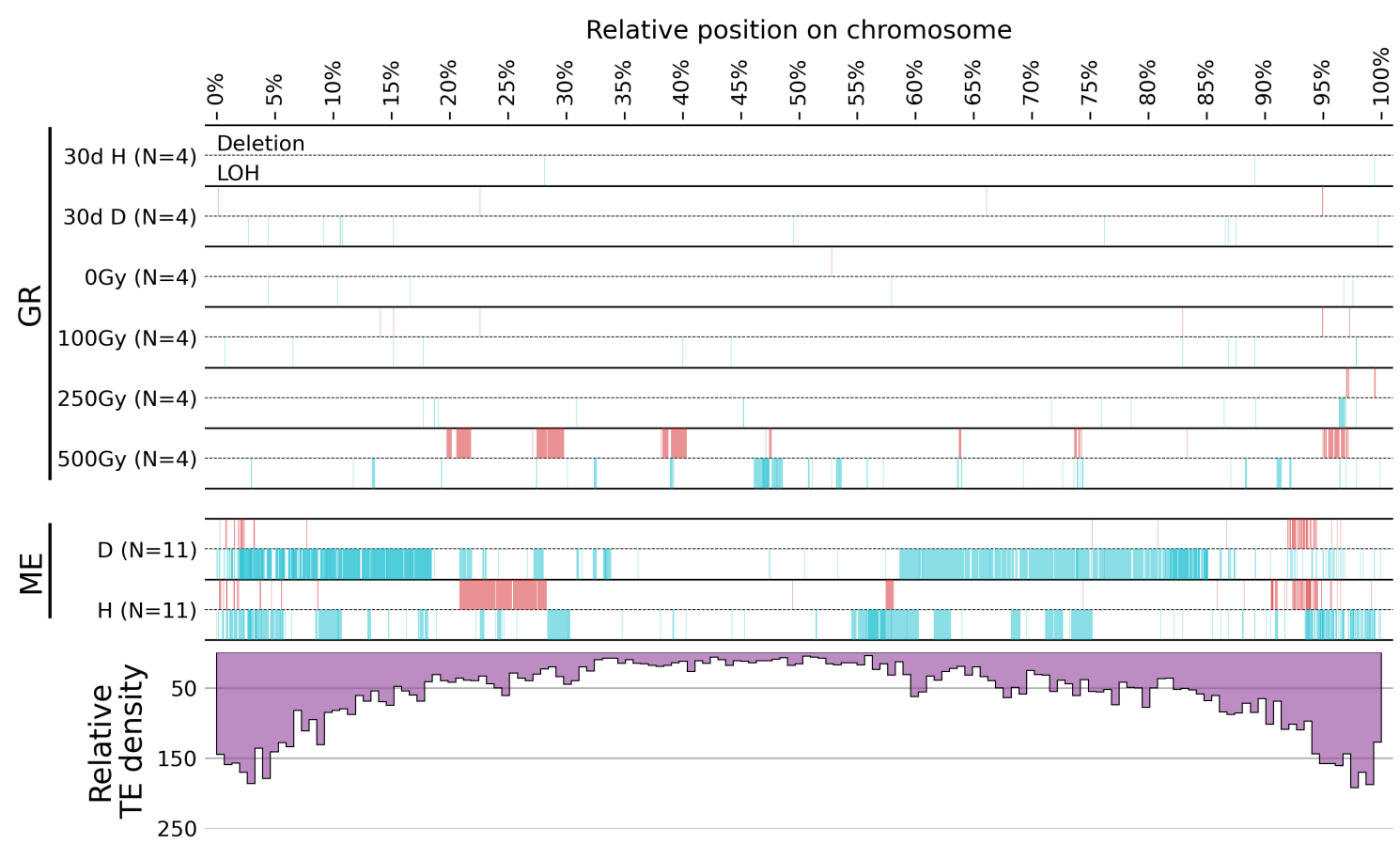

Figure S9: **Relative positions of LOH clusters per condition compared to the transposable element density.** Relative positions of LOH clusters categorized as either LOH (blue, no significant coverage drop compared to the ancestor) or deletions (red, categorized as deletion if Δ coverage is lower than -20). The position and size of each event are represented proportionally to its chromosome size. Duplications were not detected as LOH clusters and are therefore not represented here.

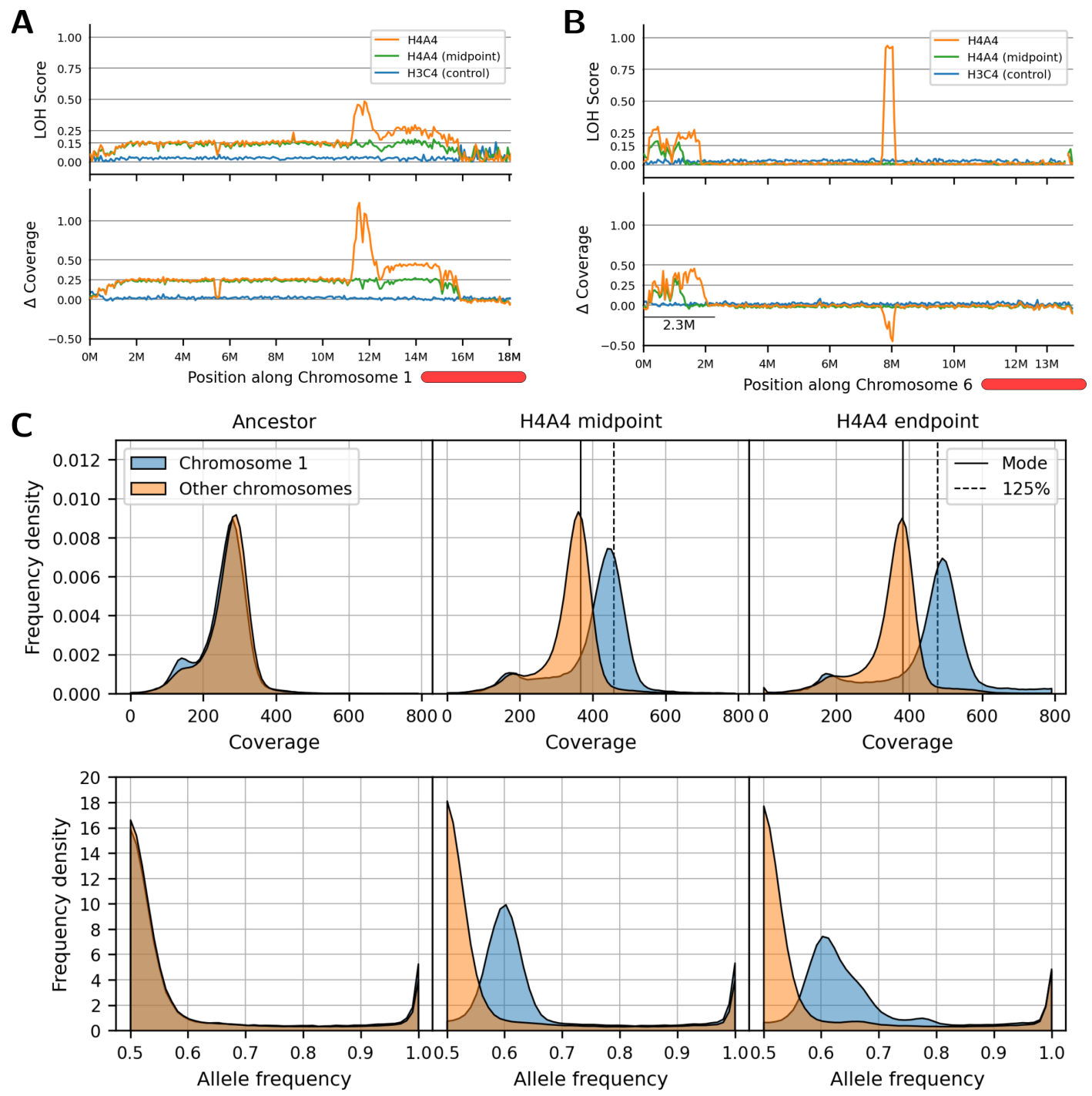

Figure S10: **Genomic evidence of a whole genome duplication and aneuploidy in ME line H4A4.** (**A-B**) LOH score (upper panel) and Δ coverage (lower panel) in sample H4A4 sequenced at midpoint (green; 61 generations) and endpoint (orange; 137 generations). Sample H3C4 is represented as a negative control without duplications. The red highlights on the cartoon chromosome (bottom right of the subfigure) indicate where the depicted regions are located along the corresponding chromosome. The duplications are limited to chromosome 1 and one extremity of chromosome 6. (**A**) Chromosome 1. The right extremity of chromosome 1 appears unduplicated because there is a size difference between the homologous chromosomes for this pair. The duplication affects only the shorter homolog. Additional copies have accumulated between midpoint endpoint in the region ranging from 11 Mb to 16 Mb. (**B**) Chromosome 6. The duplication is limited to the first 2.3 Mb on the left extremity. (**C**) Comparison of allele frequency distributions of heterozygous sites (top row) between chromosome 1 (blue) and the other chromosomes (orange) in the ancestor (left) and H4A4 midpoint (middle) and endpoint (right). Comparison of coverage distributions (bottom row) between chromosome 1 (blue) and the rest of the genome (orange). (**A-C**) The coverage and AF shifts observed on chromosome 1 are compatible with a WGD followed by an additional duplication of chromosome 1. AF of $3/5 \sim 0.6$ (LOH score of $2 \times |0.6 - 0.5| \sim 0.2$) and coverage of $5/4 = 1.25$ (Δ coverage of ~ 0.25).

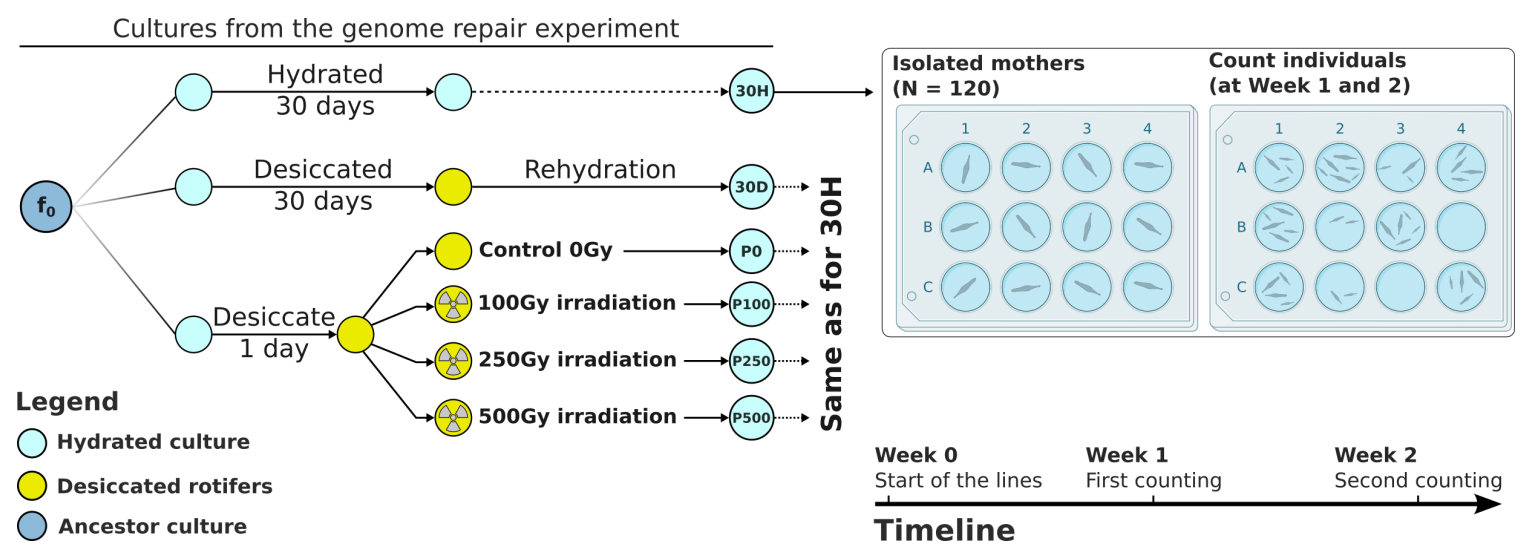

Figure S11: **Experimental design of the fertility assay.** Summary of the design of the fertility experiment. 120 individual mothers were isolated from post-treatment GR cultures immediately after rehydration. For each isolated mother, the number of active individuals in a well was counted after 1 week and then recounted after 2 weeks.

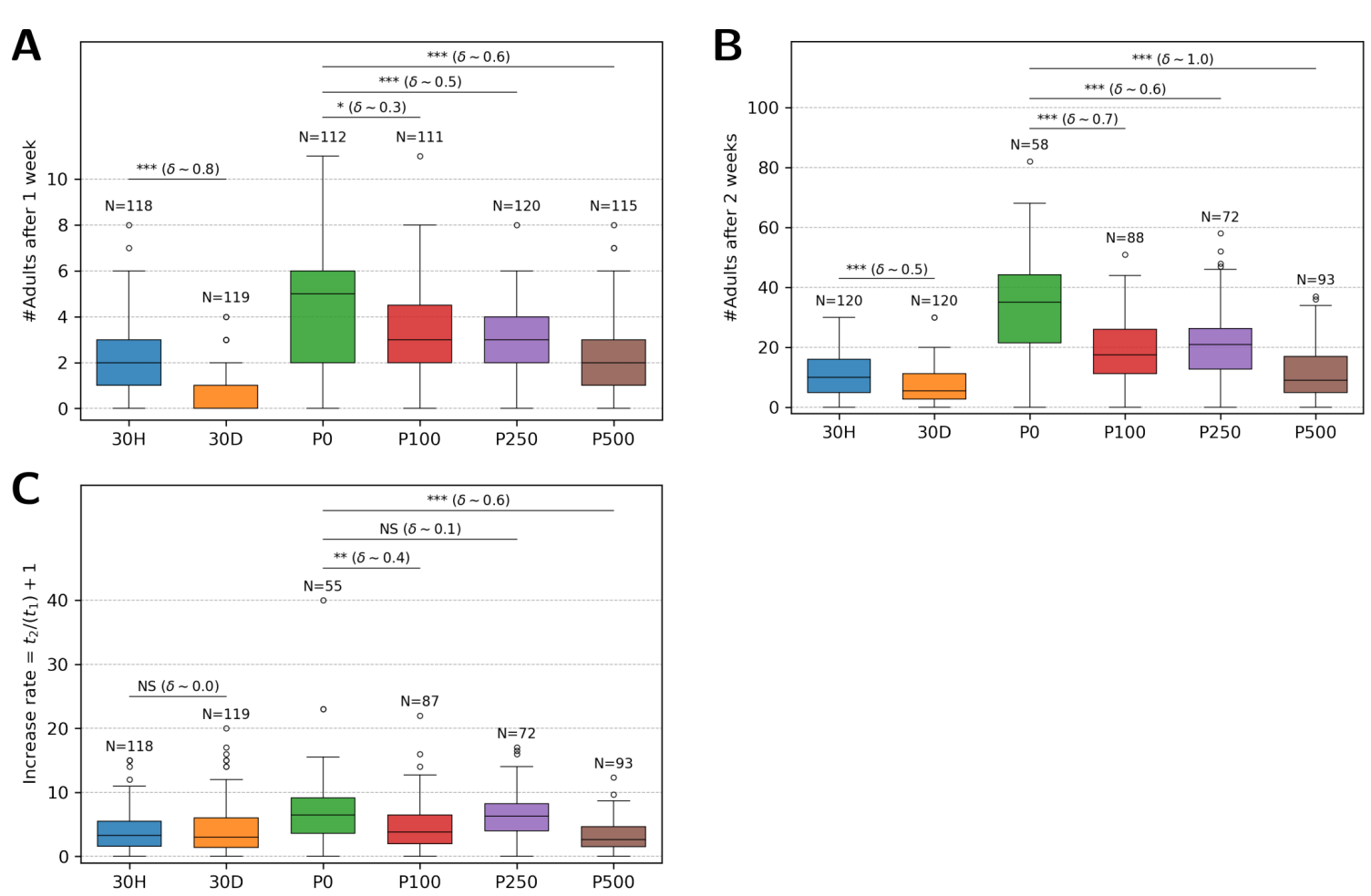

Figure S12: **Effect of different conditions on fertility of *A. vaga*.** (**A**) Number of adults counted in wells after week 1 (t_1). (**B**) Number of adults counted in wells after week 2 (t_2). (**C**) Growth rate of adult numbers between week 1 and week 2. (**A-C**) Statistical analysis corresponds to independent t-tests corrected for unequal variance. N: Sample size, δ: Glass' delta effect size statistics. NS: Non-significant; * $= p \leq 0.05$; ** $= p \leq 0.01$; *** $= p \leq 0.001$. 30H: 30 days in hydrated culture; 30D: 30 days in desiccation before rehydration; P0, P100, P250, and P500: The number indicates the proton irradiation dose before individual isolation.

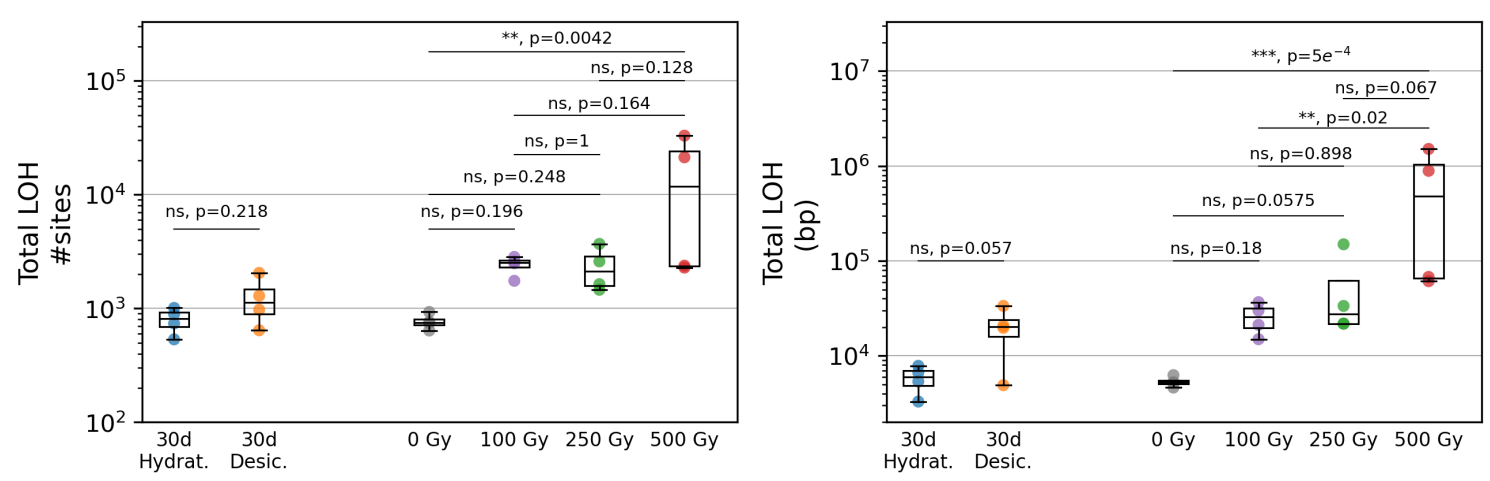

Figure S13: **Total LOH per sample and condition in the GR experiment.** (Left) Boxplots of total LOH reported as the number of ancestral heterozygous sites lost. (Right) Boxplots of total LOH reported as the minimum basepair span between the affected heterozygous sites that were lost (lower-bound estimate of the actual LOH tract size). Statistical tests were performed using Tukey's honestly significant difference (HSD) for pairwise comparisons (N = 4 per treatment); details are provided in table S3.

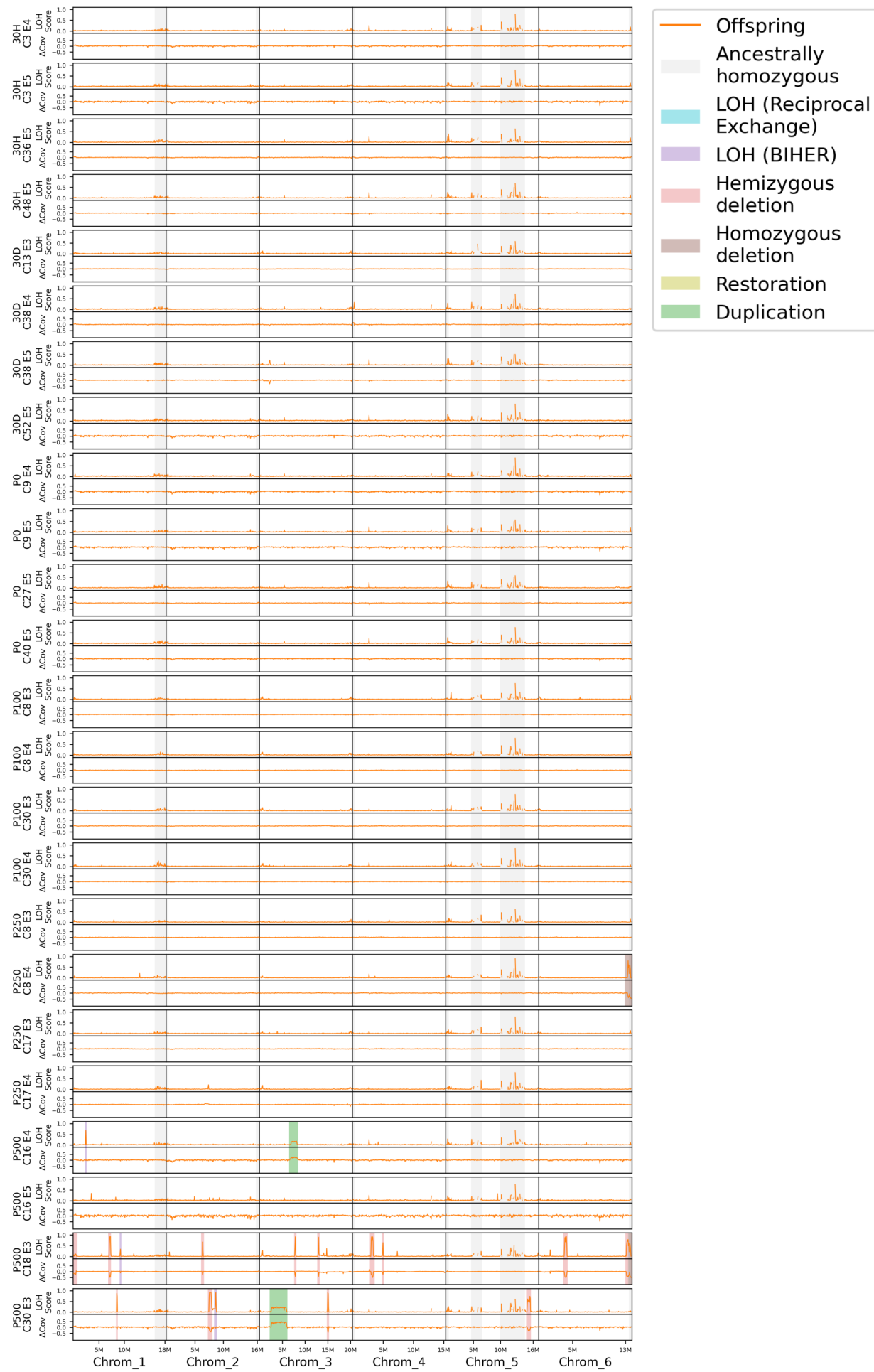

Figure S14: **Overview of LOH score and Δ coverage across all chromosomes and GR samples.** LOH score and Δ coverage are displayed across all chromosomes for the GR samples. Large events (> 50 Kb) are highlighted in color. Events include HR and reciprocal exchange (blue), hemizygous deletions (light red), homozygous deletions (dark red), restorations (yellow), and duplications (green). Ancestrally homozygous regions are also represented (grey). Values are averaged over 250 non-overlapping windows along each chromosome, with window sizes ranging from 55.6 Kb (Chromosome 6) to 81.4 Kb (Chromosome 3). The window size is defined as 1/251 of the chromosome length. Sample naming key: C = isolated mother number; E = isolated egg number. Conditions are 30 days in hydrated culture (30H), 30 days in desiccation before hydration (30D), one day in desiccation followed by 0 Gy IR (P0, control), and one day in desiccation followed by 100 (P100), 250 (P250), and 500 (P500) Gy of proton IR, respectively.

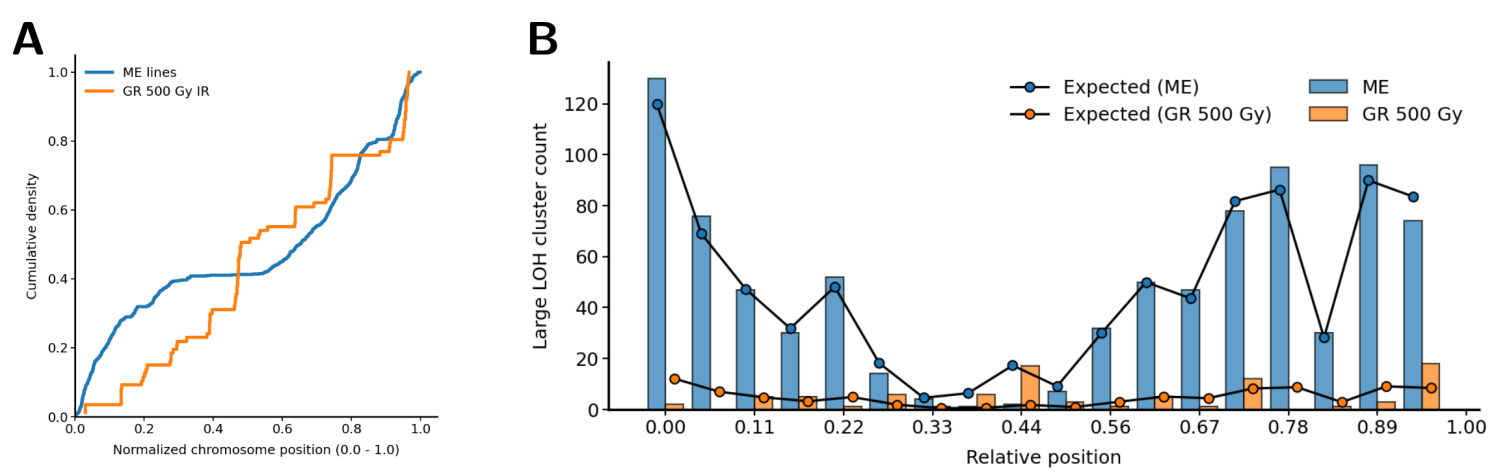

Figure S15: **Comparison of LOH cluster positions between ME and GR 500 Gy IR samples.** (**A-B**) LOH clusters are defined here as spanning more than 100 sites. (**A**) Cumulative distribution functions of cluster positions along the normalized chromosome length for ME samples (blue) and GR 500 Gy IR samples (orange). Kolmogorov-Smirnov test; $KS = 0.254$, $p = 5.33e^{-5}$. (**B**) Binned (18 windows) distribution of clusters along the normalized chromosome length. Observed counts are shown as bars, and expected values are indicated as lines for ME (blue) and GR 500 Gy (orange). Binned chi-squared test; $\chi^2 = 268.53$, $p = 3.36e^{-47}$.

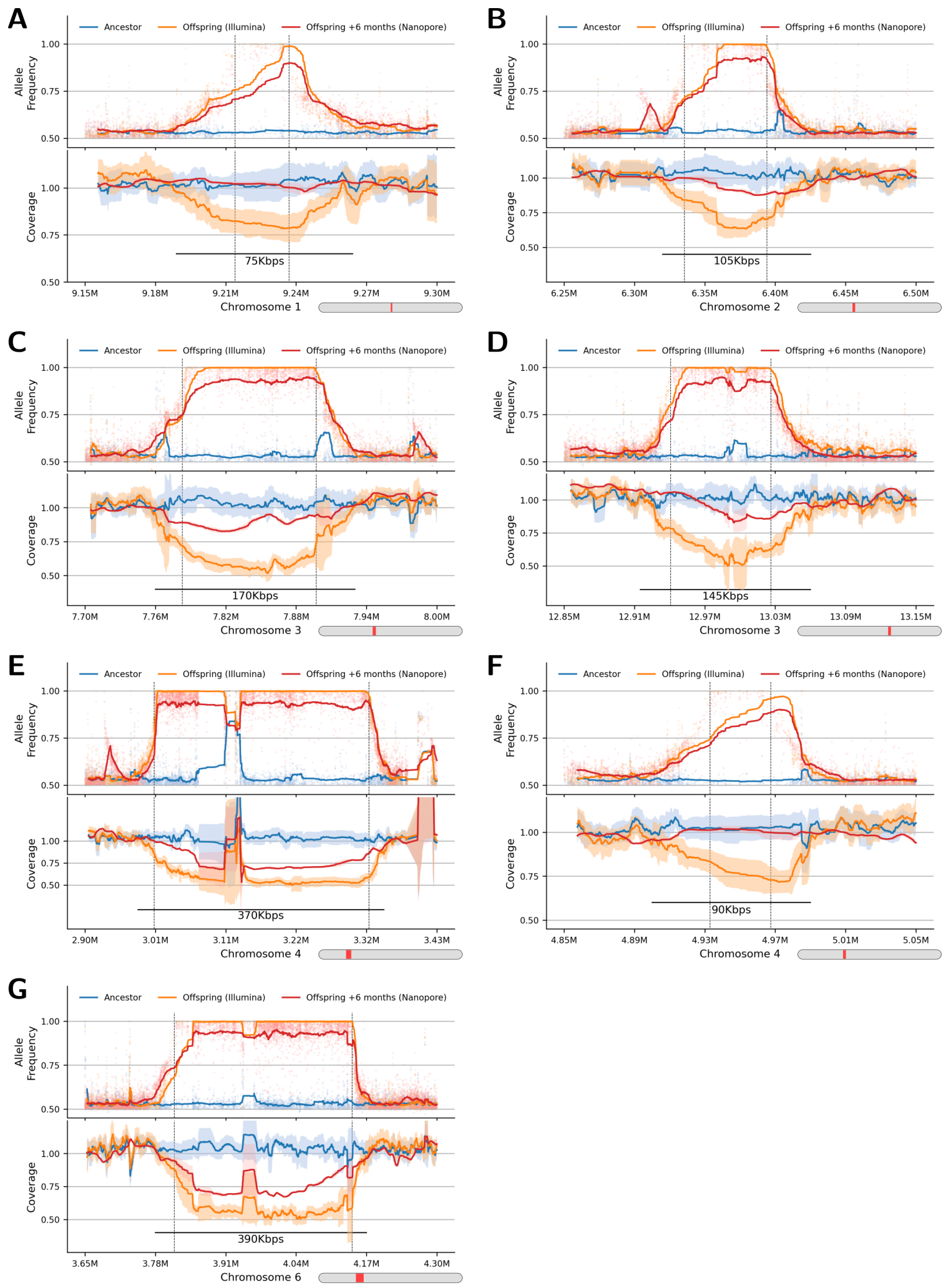

Figure S16: **AF and coverage at different timepoints in deleted regions of P500 C18 E3.** (**A-G**) Allele frequency (AF, upper panel) and coverage (lower panel) along the chromosome in regions containing deletions in the GR 500 Gy sample P500 C18 E3. Both sequencing timepoints are compared to the ancestral Illumina sequencing (blue). First timepoint (1 month post treatment; orange) and second timepoint (additional 6 months of culture; red). In the upper panel, the dots represent individual heterozygous sites, and the lines represent a sliding average. In the lower panel, the filled area corresponds to ± 1 standard deviation of coverage in the sliding window. The vertical dashed lines indicate the region of LOH (AF ∼ 1.0). The horizontal lines show the region of coverage drop. The regions where coverage and AF are shifting gradually span between 10 and 35 Kb. The red highlight on the cartoon chromosome (bottom right of each subfigure) indicates where the depicted regions are located along the corresponding chromosome.

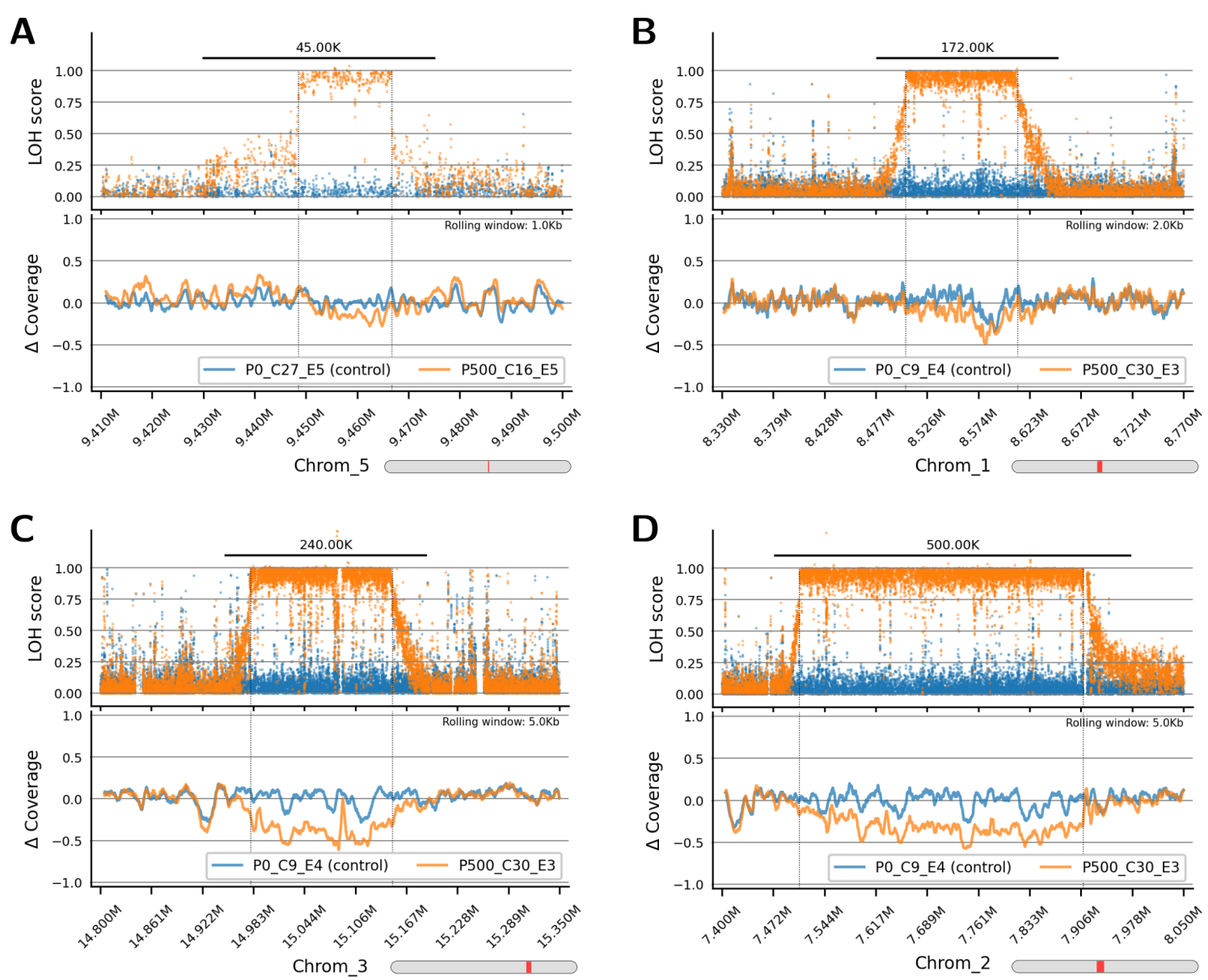

Figure S17: **Examples of variability in allele frequency (AF) and coverage in GR 500 Gy IR samples.** (**A-D**) LOH score (upper panel) and Δ coverage (lower panel, sliding window average). The red highlight on the cartoon chromosome (bottom right of each subfigure) indicates where the depicted regions are located along the corresponding chromosome. Dotted vertical lines indicate the LOH tract (LOH score ∼ 1.0). Full horizontal lines indicate the region span where LOH score and Δ coverage are shifting compared to the controls. (**A**) 500 Gy IR sample P500 C16 E5. (**B-D**) three distinct regions from the same 500 Gy IR sample (P500 C30 E3).

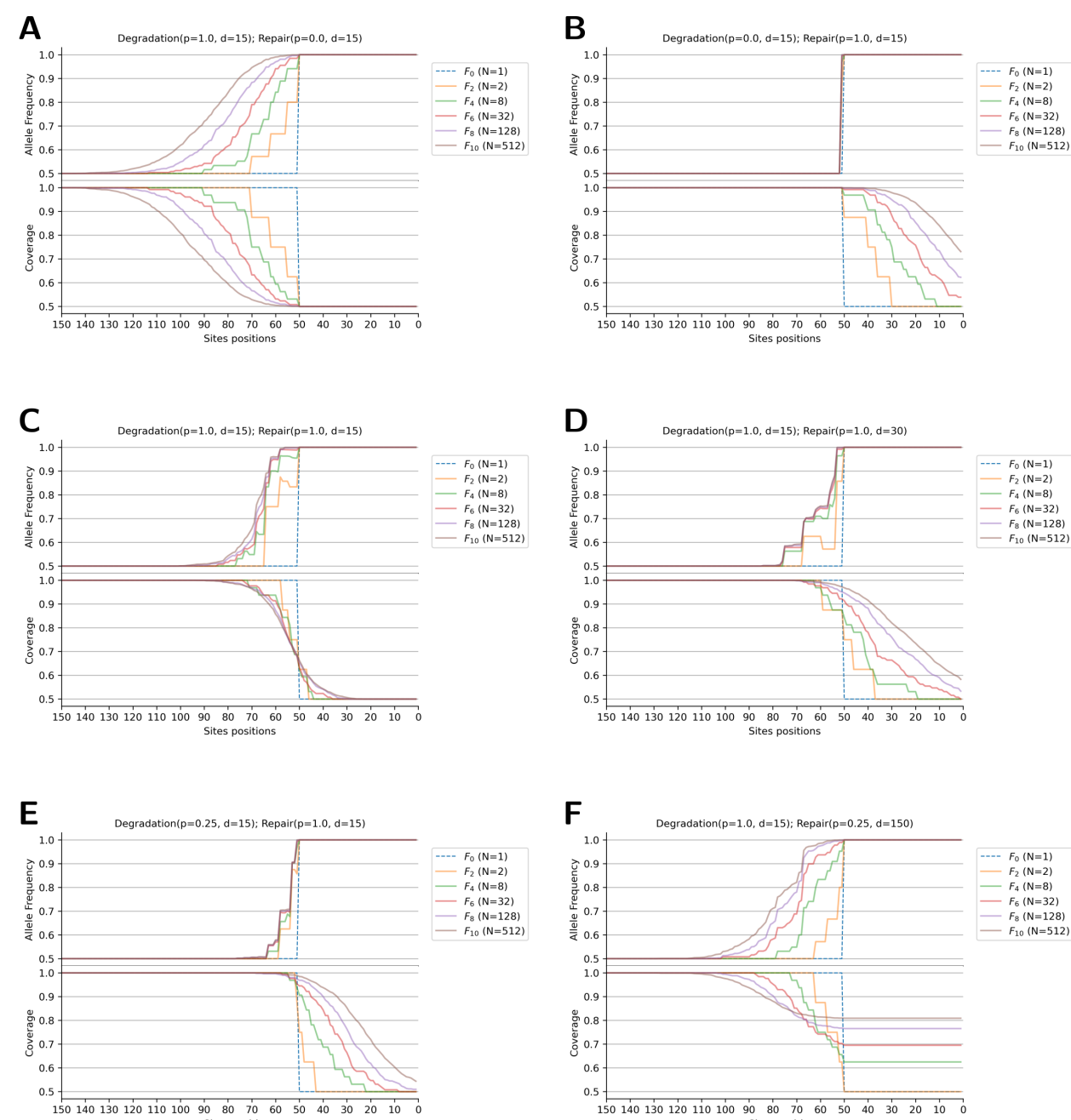

Figure S18: **Expected patterns of allele frequency (AF) and coverage under six simulated scenarios representing alternative hypotheses of repair and degradation at deletion breakpoints.** (**A**) Hypothesis of partial degradation only. (**B**) Hypothesis of partial repair only. (**C**) Hypothesis of partial degradation and partial repair with similar tract lengths. (**D**) Hypothesis of partial degradation and partial repair with longer repair tracts than degradation tracts. (**E**) Hypothesis of sporadic degradation (not occurring at every generation) but systematic partial repair. (**F**) Hypothesis of partial degradation with sporadic (not occurring at every generation) but complete and single-step repair (full recovery of the deletion at once).

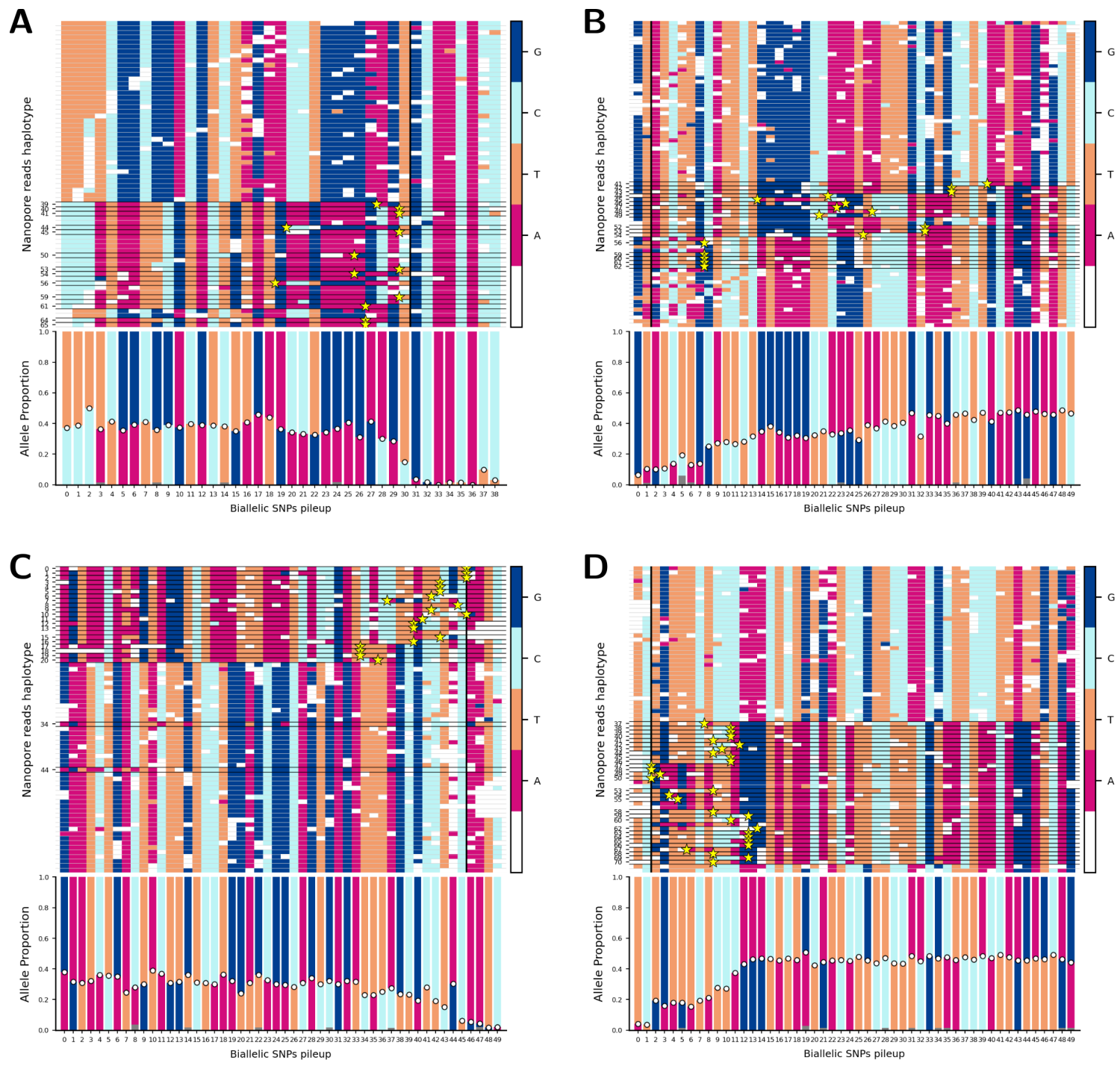

Figure S19: **Detected haplotype switches in single Nanopore reads at LOH boundaries in P500 C18 E3.** (**A-D**) The upper panel displays an alignment matrix. In the matrix, each row corresponds to a Nanopore long read alignment. Each column corresponds to a heterozygous site (only SNPs are represented) detected in a defined region (near the LOH boundary). A vertical black bar indicates where all represented reads in the matrix become homozygous (the LOH boundary). The lower panel indicates the proportion of each allele represented in the pileup for each variant (columns) position. Colors indicate which base is represented (white are undetermined/uncalled bases). Yellow stars indicate positions of haplotype switches. A subset of 30 to 50 SNPs was sampled in the considered regions. Only a subset of long reads spanning the total length of the region are shown. (**A**) Left boundary of a deletion found on chromosome 1 between 7.060 and 7.076 Mb. (**B**) Right boundary ranging from 7.3285 to 7.342 Mb. (**A-B**) The deletion corresponds to the one shown in Fig. 5A. (**C**) Left boundary of a deletion found on chromosome 4 between 4.91 and 4.94 Mb. (**D**) Right boundary ranging from 4.967 Mb to 4.985 Mb. (**C-D**) The deletion corresponds to the one shown in fig. S16D.

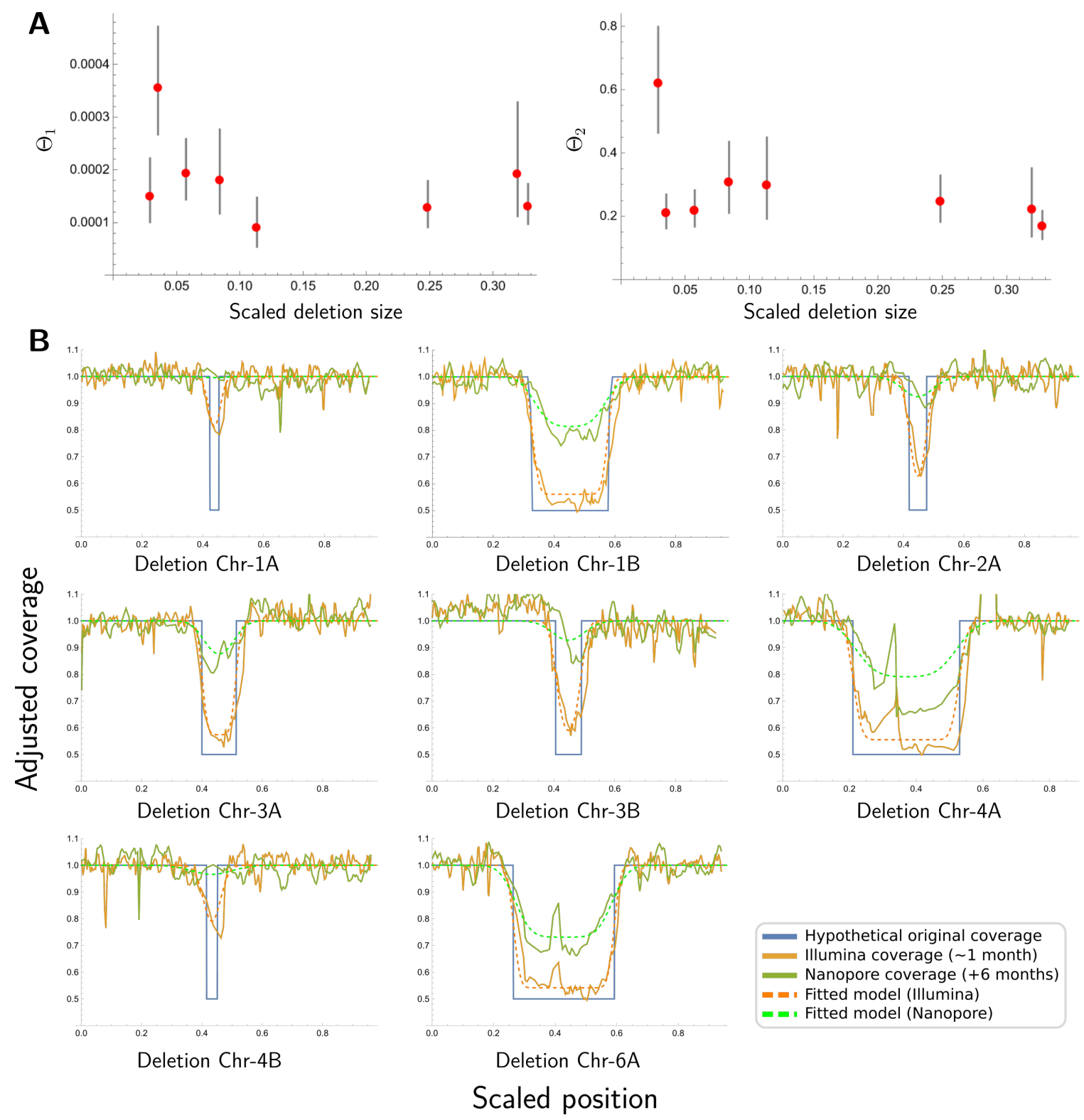

Figure S20: **Parameter estimation and function fit to observed coverage.** (**A**) Fitted values for parameters θ_1 and θ_2 are shown sorted by deletion size. These values were obtained by fitting the diffusion model to the coverage data obtained from the GR 500 Gy sample at two time points. First, after one month of culture (Illumina, orange) and second, after an additional six months of culture (Nanopore, green). (**B**) Predicted coverage (dashed lines) along the chromosome based on the fitted diffusion model.Predictions are shown for all deletions and compared to observed data (solid lines) at both timepoints. The theoretical original deletion (full blue line) corresponds to complete LOH (AF = 1.0). The deletion Chr-1B is the same as shown in Fig. 5A.

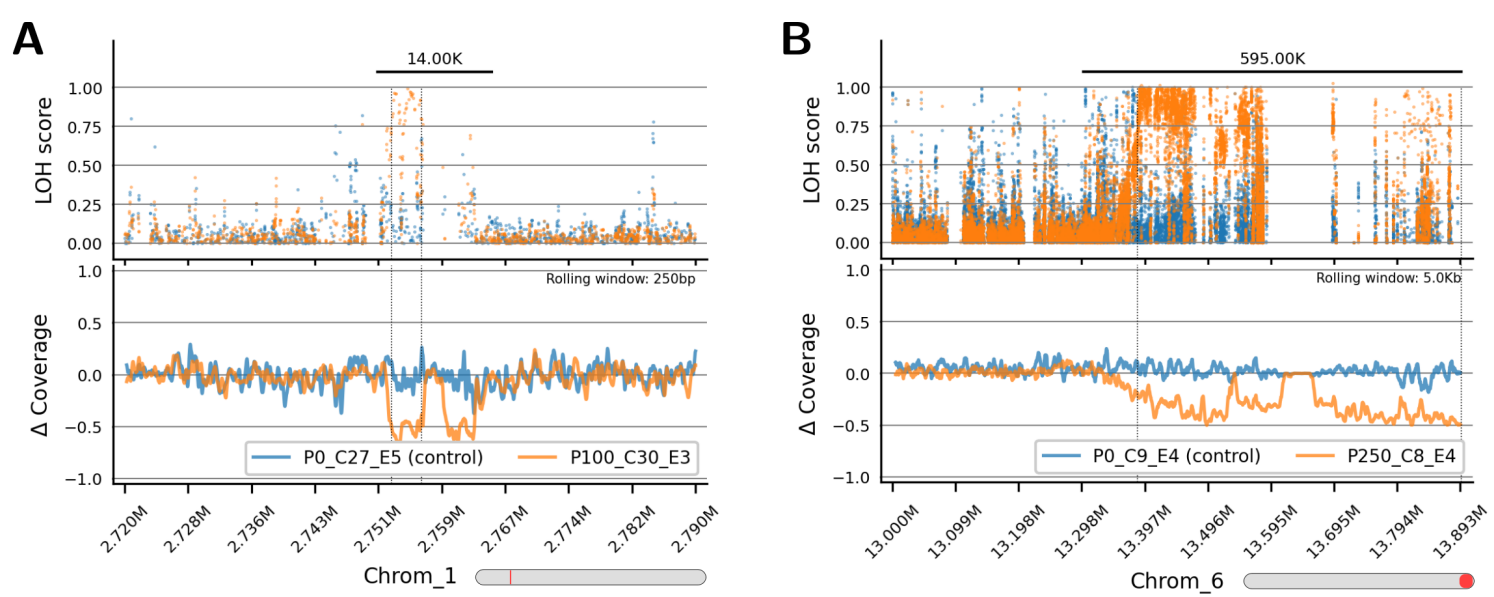

Figure S21: **Examples of variability in allele frequency (AF) and coverage in GR IR samples (100 and 250 Gy).** (**A-B**) LOH score (upper panel) and Δ coverage (lower panel, sliding window average). The red highlight on the cartoon chromosome (bottom right of each subfigure) indicates where the depicted regions are located along the corresponding chromosome. Dotted vertical lines indicate the LOH tract (LOH score ∼ 1.0). Full horizontal lines indicate the region span where LOH score and Δ coverage are shifting compared to the controls. (**A**) 100 Gy IR sample P100 C30 E3. (**B**) 250 Gy IR sample P250 C8 E4.

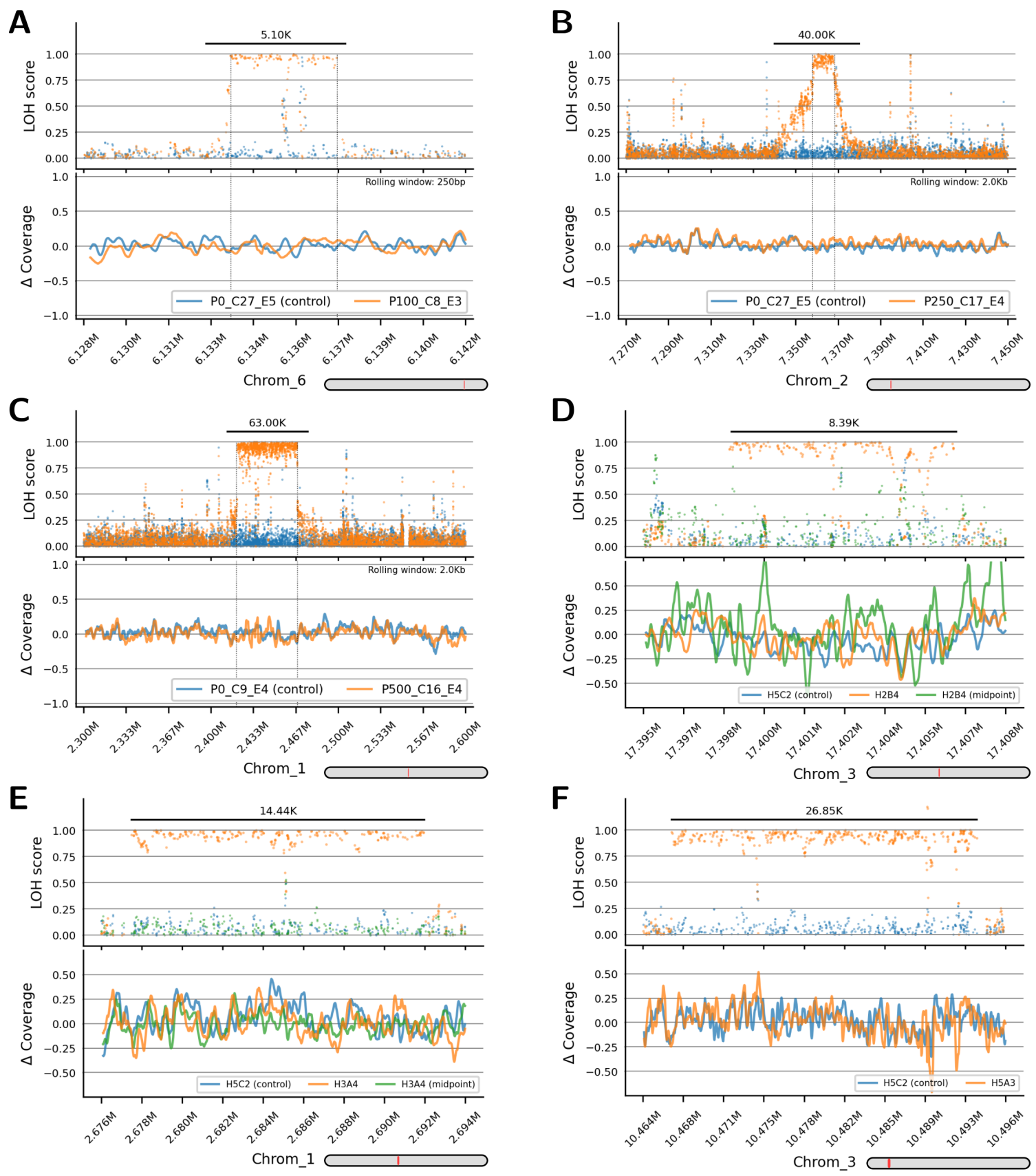

Figure S22: **Examples of long gene conversion (> 5 Kb) LOH tracts in ME and GR samples.** Examples of LOH tracts in ME and GR samples without associated coverage variations. (**A-F**) LOH score (upper panel) and Δ coverage (lower panel). For ME samples, endpoints (orange), midpoints (green), and a control without LOH (blue) are represented. For GR samples, the treatment (orange) and a control sample without LOH (blue) are represented. The red highlight on the cartoon chromosome (bottom right of each subfigure) indicates where the depicted regions are located along the corresponding chromosome. (**A-C**) Dotted vertical lines indicate the LOH tract (LOH score ~ 1.0). Full horizontal lines indicate the region span where LOH score and Δ coverage are shifting compared to the controls. (**D-F**) Full horizontal lines indicate the region span of the LOH tract (LOH score ~ 1.0). (**A**) Region of chromosome 6 of 100 Gy IR sample P100 C8 E3. (**B**) Region of chromosome 2 of 250 Gy IR sample P250 C17 E4. (**C**) Region of chromosome 1 of 500 Gy IR sample P500 C16 E4. (**D**) Region of chromosome 3 of ME line H2B4. (**E**) Region of chromosome 1 of ME line H3A4. (**F**) Region of chromosome 3 of ME line H5A3.

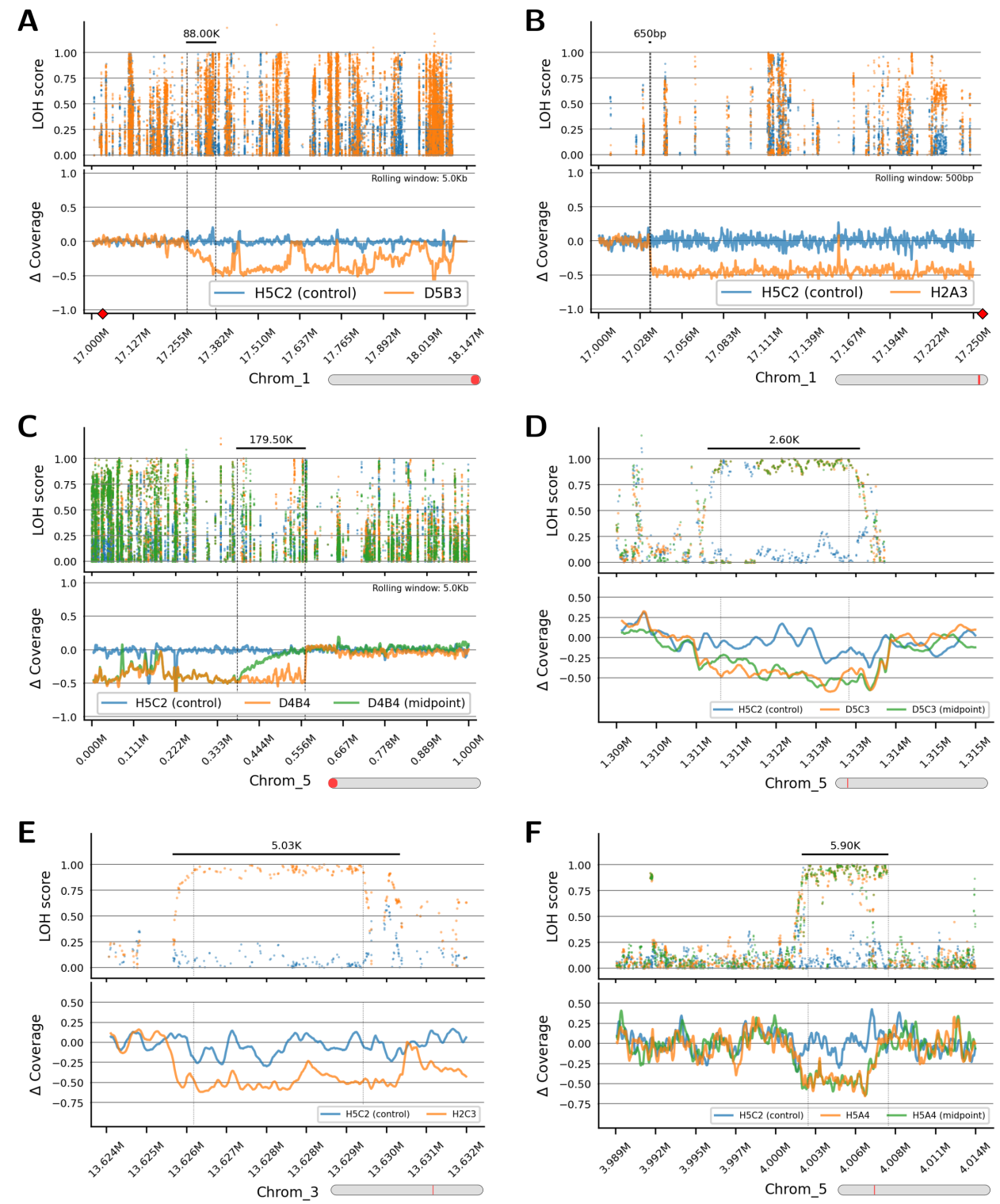

Figure S23: **Examples of Δ coverage and LOH score transitions in the ME experiment.** (**A-F**) LOH score (upper panel) and Δ coverage (lower panel). Endpoints (orange) are compared to midpoints (green), when available, and to a control sample (blue) that does not share variability in this region. The red highlight on the cartoon chromosome (bottom right of each subfigure) indicates where the depicted regions are located along the corresponding chromosome. (**A-C**) Vertical dashed lines indicate the size of the Δ coverage transition from 0 to -0.5. (**D-F**) Dotted vertical lines indicate the LOH tract (LOH score ~ 1.0). Full horizontal lines indicate the region span where LOH score and Δ coverage are shifting compared to the controls. (**A**) Gradual transition of coverage at a hemizygous deletion boundary found in ME sample D5B3. (**B**) Abrupt transition of coverage found at a hemizygous deletion in ME sample H2A3. The same region is represented in **A** and **B** and the red diamonds on the *x*-axis indicate the position where coverage starts to shift in the other example. (**C**) Comparison of midpoint and endpoint sequencing of the ME sample D4B4 where a gradual transition of coverage is found at midpoint, while an abrupt transition is found at endpoint. (**D-F**) Examples of hemizygous deletions with LOH score and Δ coverage gradual transitions at their boundaries. (**D**) Chromosome 5 of sample D5C3. (**E**) Chromosome 4 of sample D2C1. (**F**) Chromosome 5 of sample H5A4 at midpoint and endpoint.

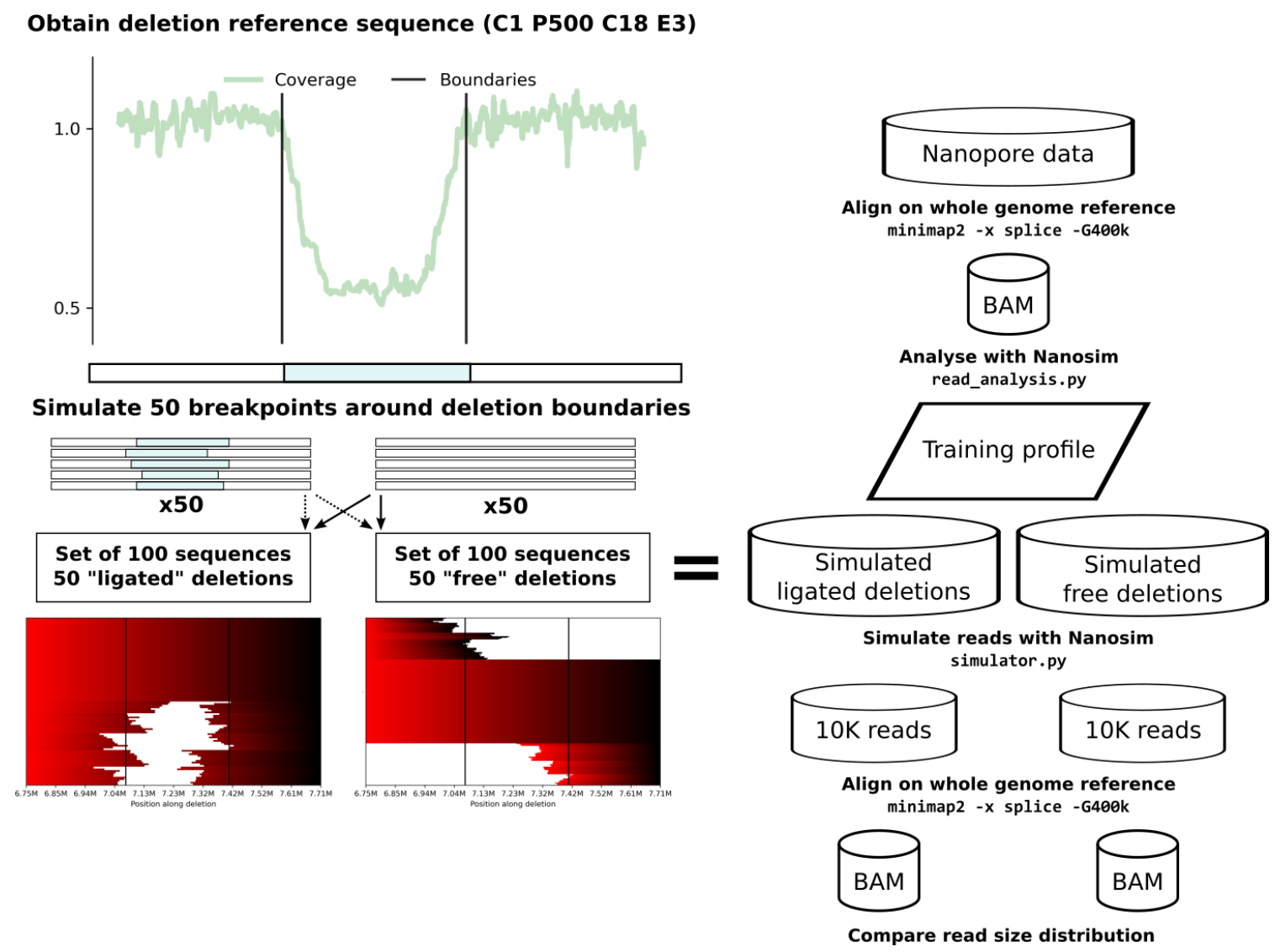

Figure S24: **Simulation design for Nanopore reads based on the representative deletion in Fig. 5A.** A reference deletion was chosen from chromosome 1 in GR sample P500 C18 E3, which contained few repetitive sequences (based on coverage variability graphs) and for which coverage in the Nanopore data was not yet restored. From this reference sequence, we generated two sets of 100 sequences each. Each set contained 50 unaltered sequences (no deletion). In the first case (ligated scenario), 50 ligation intermediates were generated *in silico*. The resulting set contained 100 independent sequences in total. In the second case (free scenario), the same breakpoints were used but remained fragmented. The resulting set contained 150 independent sequences in total. The breakpoint positions were determined using normal distributions centered around the actual LOH tract of the observed deletion, mimicking the process of resection and repair. The read profile from the real Nanopore dataset was obtained using `nanosim` and used to simulate two libraries of 10,000 Nanopore reads, one from each set. These simulated reads and the real Nanopore reads were aligned independently to the reference using `minimap2` with parameters allowing for large gapped alignments.

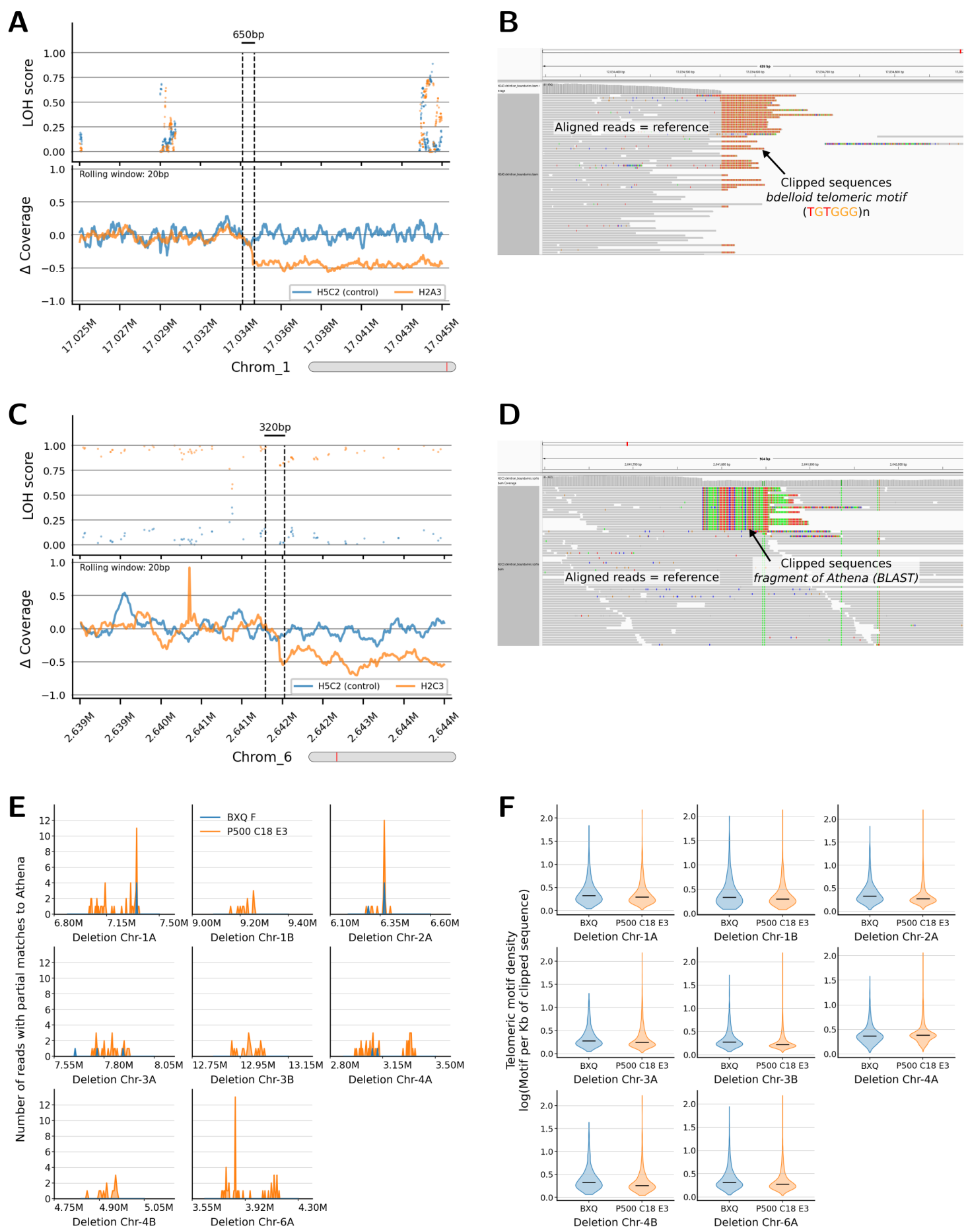

Figure S25: **Detection of telomeric motifs and fragments of the Athena retrotransposon sequence in ME and GR.** (**A**) LOH score and Δ coverage at the breakpoint of a homozygous deletion in ME sample H2A3. (**B**) IGV visualization of Illumina reads aligned at the breakpoint shown in panel **A**. Colored bases indicate the soft-clipped regions. The repeated bases (red-orange alternating) correspond to the bdelloid telomeric motif (TGTGGG)n. (**C**) LOH score and Δ coverage at the left breakpoint of a hemizygous deletion in ME sample H2C3. (**D**) IGV visualization of Illumina reads aligned at the breakpoint shown in panel **C**. Colored bases indicate the soft-clipped regions. The clipped bases BLAST against the Athena retrotransposon. (**A**,**C**) The red highlight on the cartoon chromosome (bottom right) indicates where the depicted regions are located along the corresponding chromosome. (**E**) Athena hits found in clipped bases of the Nanopore reads aligned in the large deletions of P500 C18 E3 compared to a hydrated culture Nanopore sequencing BXQ F, accession: SRR13348928 (*19*). (**F**) Density of the bdelloid telomeric motif found in clipped bases of the Nanopore reads aligned to the large deletions in P500 C18 E3 (compared to BXQ F).

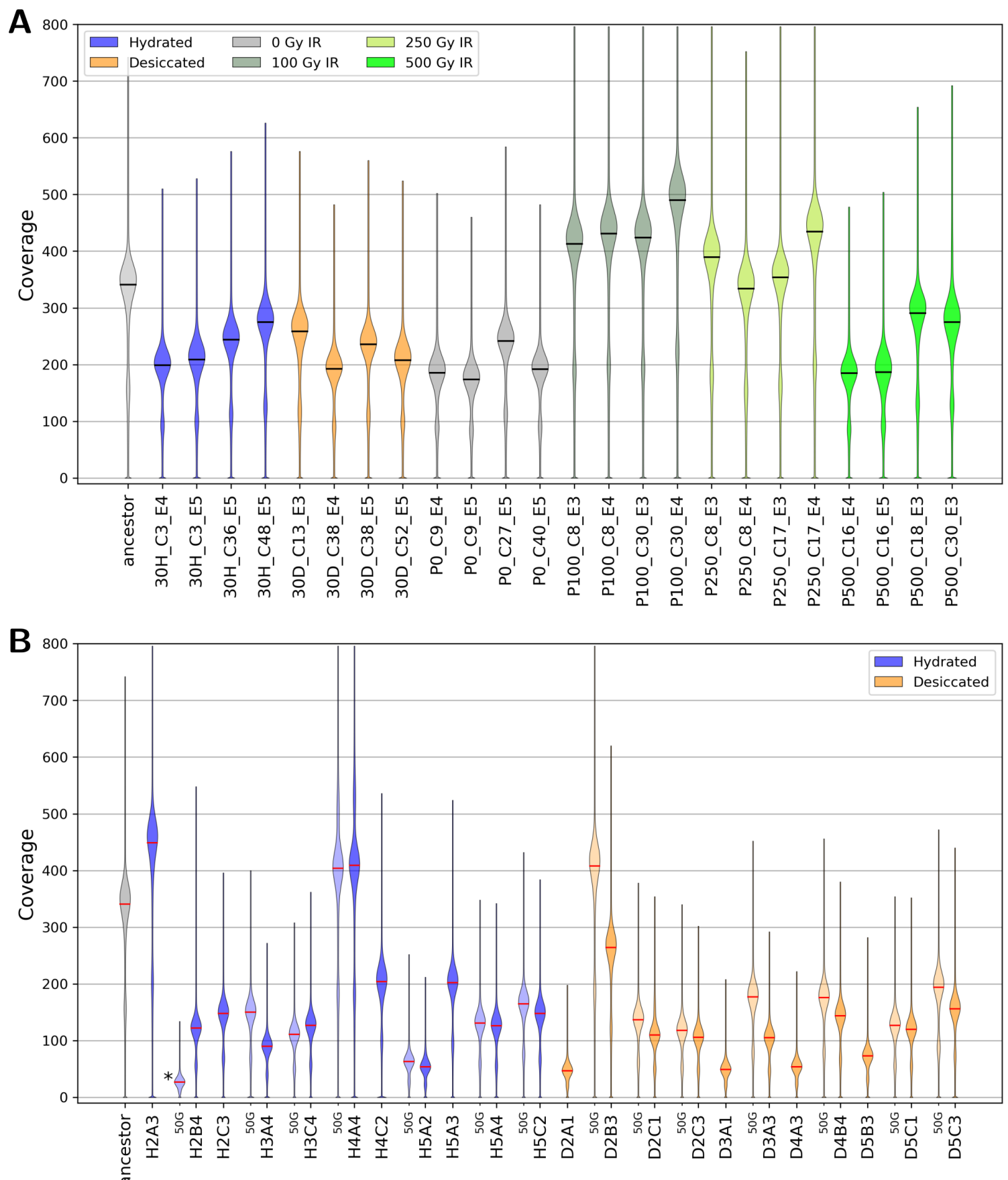

Figure S26: **Coverage distributions per sample (with concatenated libraries when multiple sequencing rounds).** (**A-B**) Coverage depth distribution per sample categorized per condition in color. (**A**) GR samples. Hydrated (blue), desiccated (orange), 0 Gy IR (gray), 100 Gy IR (light green), 250 Gy IR (green), and 500 Gy IR (dark green). (**B**) ME samples. Midpoints (50G) are shown in lighter shades and endpoints in darker shades. An asterisk (*) indicates the lowest coverage sample in ME (H2B4 midpoint) resulting from a technical problem in sequencing.

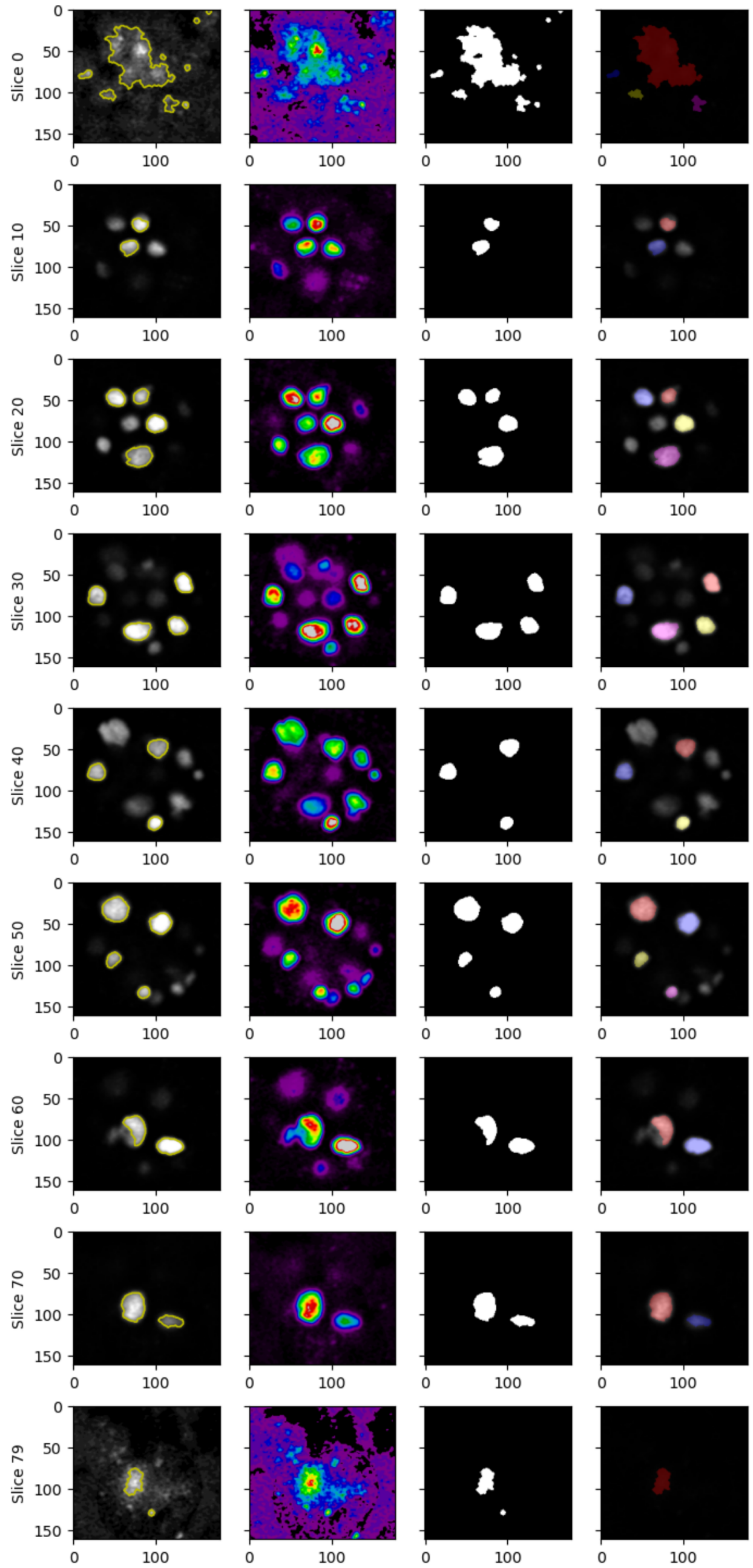

Figure S27: **Automatic segmentation of karyotype z-stacks.** Example of slices with auto-detection (segmentation) of chromosomes in a z-stack corresponding to a 1-cell egg of P500 C18 E3. Columns, from left to right, correspond to the original slice with contour detection, fluorescence intensity, segmentation layers, and detection of distinct fragments tracked between layers. For representation, the figure shows every tenth slice, but all of them were analyzed.

Titles and captions of the supplementary tables

Table S1: **Sample metadata and sequencing information**

Caption: Overview of all samples used in this study, including experimental context, sequencing metadata, and quality metrics. Corresponding Sequence Read Archive (SRA) accession numbers are provided for data availability. This file also contains sheets with ME lines per generation as well as a summary of large genomic events detected between the ancestor and the sequenced set.

Table S2: **Expected signatures of genomic events**

Caption: Summary of expected allele frequency (AF), coverage, LOH score, and Δ coverage patterns for different classes of genomic events. Events include LOH, hemizygous deletion, homozygous deletion, restoration, and duplication, with values shown for ancestral and offspring states. For each event type, the table specifies genotype transitions, expected AF values, normalized coverage, LOH scores, and Δ coverage signals. These theoretical signatures provide a reference framework for interpreting observed genomic patterns and for classifying events. The table was used to guide event annotation and to distinguish between mechanisms such as deletion, duplication, and repair.

Table S3: **Summary statistics and statistical models**

Caption: Summary statistics and statistical analyses of heterozygosity, LOH, coverage dynamics, cytogenetics, and fertility across samples and conditions. Different sheets summarize different datasets and analyses including: per-sample estimates of total heterozygosity and LOH levels; Comparisons of heterozygosity and LOH rates across conditions or lineages; Dataset for linear mixed-effects models, including predictors and response variables; Results of linear mixed-effects models testing effects of condition, time, or treatment; Results of ordinary least squares regression analyses for selected relationships; Dose–response analysis of LOH as a function of irradiation intensity in GR experiments; Comparison of LOH patterns between conditions (e.g., H vs D) in GR samples; Human chromosome fragment counts from microscopy, including statistical comparisons; Automated (computer vision) detection of chromosome fragments and associated metrics; Fertility measurements and associated statistical analyses across samples or treatments. Together, these sheets provide the quantitative and statistical framework supporting the study's conclusions on

REFERENCES

1. L. Nguyen, J. W. M. Martens, A. Van Hoeck, E. Cuppen, Pan-cancer landscape of homologous recombination deficiency. *Nat. Commun.* **11**, 5584 (2020).

2. D. Zickler, N. Kleckner, Recombination, pairing, and synapsis of homologs during meiosis. *Cold Spring Harb. Perspect. Biol.* **7**, a016626 (2015).

3. S. P. Otto, The evolutionary enigma of sex. *Am. Nat.* **174**, S1–S14 (2009).

4. S. Glemin, N. Galtier, "Genome evolution in outcrossing versus selfing versus asexual species," in *Evolutionary Genomics* (Springer, 2012), pp. 311–335.

5. B. Alberts, *Molecular Biology of the Cell* (WW Norton & Company, 2017).

6. C. Ricci, Bdelloid rotifers:'sleeping beauties' and 'evolutionary scandals', but not only. *Hydrobiologia* **796**, 277–285 (2017).

7. D. B. M. Welch, M. Meselson, Evidence for the evolution of bdelloid rotifers without sexual reproduction or genetic exchange. *Science* **288**, 1211–1215 (2000).

8. E. Gladyshev, M. Meselson, Extreme resistance of bdelloid rotifers to ionizing radiation. *Proc. Natl. Acad. Sci. U.S.A.* **105**, 5139–5144 (2008).

9. A. Krisko, M. Leroy, M. Radman, M. Meselson, Extreme anti-oxidant protection against ionizing radiation in bdelloid rotifers. *Proc. Natl. Acad. Sci. U.S.A.* **109**, 2354–2357 (2012).

10. B. Hespeels, S. Penninckx, V. Cornet, L. Bruneau, C. Bopp, V. Baumlé, B. Redivo, A. C. Heuskin, R. Moeller, A. Fujimori, S. Lucas, K. van Doninck, Iron ladies—How desiccated asexual rotifer *Adineta vaga* deal with x-rays and heavy ions? *Front. Microbiol.* **11**, 1792 (2020).

11. V. C. Moris, L. Bruneau, J. Berthe, A. C. Heuskin, S. Penninckx, S. Ritter, U. Weber, M. Durante, E. G. J. Danchin, B. Hespeels, K. V. Doninck, Ionizing radiation responses appear incidental to desiccation responses in the bdelloid rotifer Adineta vaga. *BMC Biol.* **22**, 11 (2024).

12. C. Boschetti, C. Ricci, C. Sotgia, U. Fascio, The development of a bdelloid egg: A contribution after 100 years. *Hydrobiologia* **546**, 323–331 (2005).

13. B. Hespeels, M. Knapen, D. Hanot-Mambres, A. C. Heuskin, F. Pineux, S. Lucas, R. Koszul, K. van Doninck, Gateway to genetic exchange? DNA double-strand breaks in the bdelloid rotifer Adineta vaga submitted to desiccation. *J. Evol. Biol.* **27**, 1334–1345 (2014).

14. M. Terwagne, E. Nicolas, B. Hespeels, L. Herter, J. Virgo, C. Demazy, A. C. Heuskin, B. Hallet, K. van Doninck, DNA repair during nonreductional meiosis in the asexual rotifer Adineta vaga. *Sci. Adv.* **8**, eadc8829 (2022).

15. E. Nicolas, P. Simion, M. Guérineau, M. Terwagne, M. Colinet, J. Virgo, M. Lingurski, A. Boutsen, M. Dieu, B. Hallet, K. van Doninck, Horizontal acquisition of a DNA ligase improves DNA damage tolerance in eukaryotes. *Nat. Commun.* **14**, 7638 (2023).

16. N. Debortoli, X. Li, I. Eyres, D. Fontaneto, B. Hespeels, C. Q. Tang, J. F. Flot, K. van Doninck, Genetic exchange among bdelloid rotifers is more likely due to horizontal gene transfer than to meiotic sex. *Curr. Biol.* **26**, 723–732 (2016).

17. O. A. Vakhrusheva, E. A. Mnatsakanova, Y. R. Galimov, T. V. Neretina, E. S. Gerasimov, S. A. Naumenko, S. G. Ozerova, A. O. Zalevsky, I. A. Yushenova, F. Rodriguez, I. R. Arkhipova, A. A. Penin, M. D. Logacheva, G. A. Bazykin, A. S. Kondrashov, Genomic signatures of recombination in a natural population of the bdelloid rotifer Adineta vaga. *Nat. Commun.* **11**, 6421 (2020).

18. V. N. Laine, T. B. Sackton, M. Meselson, Genomic signature of sexual reproduction in the bdelloid rotifer Macrotrachella quadricornifera. *Genetics* **220**, iyab221 (2022).

19. P. Simion, J. Narayan, A. Houtain, A. Derzelle, L. Baudry, E. Nicolas, R. Arora, M. Cariou, C. Cruaud, F. R. Gaudray, C. Gilbert, N. Guiglielmoni, B. Hespeels, D. K. L. Kozlowski, K. Labadie, A. Limasset, M. Llirós, M. Marbouty, M. Terwagne, J. Virgo, R. Cordaux, E. G. J. Danchin, B. Hallet, R. Koszul, T. Lenormand, J. F. Flot, K. van Doninck, Chromosome-level

genome assembly reveals homologous chromosomes and recombination in asexual rotifer Adineta vaga. *Sci. Adv.* **7**, eabg4216 (2021).

20. C. G. Wilson, T. Pieszko, R. W. Nowell, T. G. Barraclough, Recombination in bdelloid rotifer genomes: Asexuality, transfer and stress. *Trends Genet.* **40**, 422–436 (2024).

21. V. Katju, U. Bergthorsson, Old trade, new tricks: Insights into the spontaneous mutation process from the partnering of classical mutation accumulation experiments with high-throughput genomic approaches. *Genome Biol. Evol.* **11**, 136–165 (2019).

22. S. Silaiyiman, J. Liu, J. Wu, L. Ouyang, Z. Cao, C. Shen, A systematic review of the advances and new insights into copy number variations in plant genomes. *Plants* **14**, 1399 (2025).

23. R. Ceccaldi, B. Rondinelli, A. D. D'Andrea, Repair pathway choices and consequences at the double-strand break. *Trends Cell Biol.* **26**, 52–64 (2016).

24. H. H. Chang, N. R. Pannunzio, N. Adachi, M. R. Lieber, Non-homologous DNA end joining and alternative pathways to double-strand break repair. *Nat. Rev. Mol. Cell Biol.* **18**, 495–506 (2017).

25. J. Engelstädter, Asexual but not clonal: Evolutionary processes in automictic populations. *Genetics* **206**, 993–1009 (2017).

26. C. Smukowski Heil, Loss of heterozygosity and its importance in evolution. *J. Mol. Evol.* **91**, 369–377 (2023).

27. M. J. McDonald, D. P. Rice, M. M. Desai, Sex speeds adaptation by altering the dynamics of molecular evolution. *Nature* **531**, 233–236 (2016).

28. K. Janko, O. Bartoš, J. Kočí, J. Roslein, E. J. Drdová, J. Kotusz, J. Eisner, M. Mokrejš, E. Štefková-Kašparová, Genome fractionation and loss of heterozygosity in hybrids and polyploids: Mechanisms, consequences for selection, and link to gene function. *Mol. Biol. Evol.* **38**, 5255–5274 (2021).

29. R. W. Nowell, P. Almeida, C. G. Wilson, T. P. Smith, D. Fontaneto, A. Crisp, G. Micklem, A. Tunnacliffe, C. Boschetti, T. G. Barraclough, Comparative genomics of bdelloid rotifers: Insights from desiccating and nondesiccating species. *PLoS Biol.* **16**, e2004830 (2018).

30. K. S. Jaron, J. Bast, R. W. Nowell, T. R. Ranallo-Benavidez, M. Robinson-Rechavi, T. Schwander, Genomic features of parthenogenetic animals. *J. Hered.* **112**, 19–33 (2021).

31. J.-F. Flot, B. Hespeels, X. Li, B. Noel, I. Arkhipova, E. G. J. Danchin, A. Hejnol, B. Henrissat, R. Koszul, J. M. Aury, V. Barbe, R. M. Barthélémy, J. Bast, G. A. Bazykin, O. Chabrol, A. Couloux, M. da Rocha, C. da Silva, E. Gladyshev, P. Gouret, O. Hallatschek, B. Hecox-Lea, K. Labadie, B. Lejeune, O. Piskurek, J. Poulain, F. Rodriguez, J. F. Ryan, O. A. Vakhrusheva, E. Wajnberg, B. Wirth, I. Yushenova, M. Kellis, A. S. Kondrashov, D. B. Mark Welch, P. Pontarotti, J. Weissenbach, P. Wincker, O. Jaillon, K. van Doninck, Genomic evidence for ameiotic evolution in the bdelloid rotifer Adineta vaga. *Nature* **500**, 453–457 (2013)

32. C. Oger, C. Claeys Bouuaert, In vitro reconstitution of SPO11-mediated DNA cleavage sheds new light on the initiation of meiotic recombination. *DNA Cell Biol.* **44**, 401–406 (2025).

33. I. V. Mavragani, Z. Nikitaki, M. Souli, A. Aziz, S. Nowsheen, K. Aziz, E. Rogakou, A. Georgakilas, Complex DNA damage: A route to radiation-induced genomic instability and carcinogenesis. *Cancers* **9**, 91 (2017).

34. S. Timm, Y. Lorat, B. Jakob, G. Taucher-Scholz, C. E. Rübe, Clustered DNA damage concentrated in particle trajectories causes persistent large-scale rearrangements in chromatin architecture. *Radiother. Oncol.* **129**, 600–610 (2018)

35. H. Liu, J. Huang, X. Sun, J. Li, Y. Hu, L. Yu, G. Liti, D. Tian, L. D. Hurst, S. Yang, Tetrad analysis in plants and fungi finds large differences in gene conversion rates but no GC bias. *Nat. Ecol. Evol.* **2**, 164–173 (2018)

36. P. Cejka, L. S. Symington, DNA end resection: Mechanism and control. *Annu. Rev. Genet.* **55**, 285–307 (2021).

37. X. Wu, A. Malkova, Break-induced replication mechanisms in yeast and mammals. *Curr. Opin. Genet. Dev.* **71**, 163–170 (2021).

38. R. Scully, R. Elango, A. Panday, N. A. Willis, Recombination and restart at blocked replication forks. *Curr. Opin. Genet. Dev.* **71**, 154–162 (2021).

39. L. Liu, A. Malkova, Break-induced replication: Unraveling each step. *Trends Genet.* **38**, 752–765 (2022).

40. M. D. Adams, M. McVey, J. J. Sekelsky, *Drosophila* BLM in double-strand break repair by synthesis-dependent strand annealing. *Science* **299**, 265–267 (2003).

41. M. McVey, M. Adams, E. Staeva-Vieira, J. J. Sekelsky, Evidence for multiple cycles of strand invasion during repair of double-strand gaps in Drosophila. *Genetics* **167**, 699–705 (2004).

42. F. Zedek, P. Bureš, Holocentric chromosomes: From tolerance to fragmentation to colonization of the land. *Ann. Bot.* **121**, 9–16 (2018).

43. H. Muller, J. Gil, I. A. Drinnenberg, The impact of centromeres on spatial genome architecture. *Trends Genet.* **35**, 565–578 (2019).

44. A. P. Senaratne, N. Cortes-Silva, I. A. Drinnenberg, Evolution of holocentric chromosomes: Drivers, diversity, and deterrents. *Semin. Cell Dev. Biol.* **127**, 90–99 (2022).

45. P. M. Carlton, R. E. Davis, S. Ahmed, Nematode chromosomes. *Genetics* **221**, iyac014 (2022).

46. M. Mandrioli, G. C. Manicardi, Holocentric chromosomes. *PLOS Genet.* **16**, e1008918 (2020).

47. K. Finn, N. F. Lowndes, M. Grenon, Eukaryotic DNA damage checkpoint activation in response to double-strand breaks. *Cell. Mol. Life Sci.* **69**, 1447–1473 (2012).

48. F. Sun, P. Sutovsky, A. L. Patterson, A. Z. Balboula, *Mechanisms of DNA Damage Response in Mammalian Oocytes* (Springer International Publishing, 2024), pp. 47–68, doi:https://doi.org/10.1007/978-3-031-55163-5_3.

49. M. B. Kastan, DNA damage responses: Mechanisms and roles in human disease: 2007 GHA Clowes Memorial Award Lecture. *Mol. Cancer Res.* **6**, 517–524 (2008).

50. P. L. Ghospurkar, T. M. Wilson, A. L. Severson, S. J. Klein, S. K. Khaku, A. P. Walther, S. J. Haring, The DNA damage response and checkpoint adaptation in Saccharomyces cerevisiae: Distinct roles for the replication protein A2 (Rfa2) N-terminus. *Genetics* **199**, 711–727 (2015).

51. E. Shor, D. S. Perlin, DNA damage response of major fungal pathogen Candida glabrata offers clues to explain its genetic diversity. *Curr. Genet.* **67**, 439–445 (2021).

52. M. J. Jones, P. V. Jallepalli, Chromothripsis: Chromosomes in crisis. *Dev. Cell* **23**, 908–917 (2012).

53. A. Mazzagatti, J. L. Engel, P. Ly, Boveri and beyond: Chromothripsis and genomic instability from mitotic errors. *Mol. Cell* **84**, 55–69 (2024).

54. T. De Lange, How telomeres solve the end-protection problem. *Science* **326**, 948–952 (2009).

55. A. Decottignies, Alternative end-joining mechanisms: A historical perspective. *Front. Genet.* **4**, 48 (2013).

56. M. Betermier, S. Duharcourt, "Programmed rearrangement in ciliates: Paramecium," in *Mobile DNA III* (2015), pp. 369–388, doi:https://doi.org/10.1128/microbiolspec.mdna3-0035-2014.

57. I. Rzeszutek, X. X. Maurer-Alcalá, M. Nowacki, Programmed genome rearrangements in ciliates. *Cell. Mol. Life Sci.* **77**, 4615–4629 (2020).

58. K. M. Kramer, J. E. Haber, New telomeres in yeast are initiated with a highly selected subset of TG1-3 repeats. *Genes Dev.* **7**, 2345–2356 (1993).

59. C. N. Sprung, G. E. Reynolds, M. Jasin, J. P. Murnane, Chromosome healing in mouse embryonic stem cells. *Proc. Natl. Acad. Sci. U.S.A.* **96**, 6781–6786 (1999).

60. C. G. Kinzig, G. Zakusilo, K. K. Takai, L. R. Myler, T. de Lange, ATR blocks telomerase from converting DNA breaks into telomeres. *Science* **383**, 763–770 (2024).

61. I. R. Arkhipova, I. A. Yushenova, F. Rodriguez, Giant reverse transcriptase-encoding transposable elements at telomeres. *Mol. Biol. Evol.* **34**, 2245–2257 (2017).

62. A. Vasilikopoulos, P. Simion, A. Houtain, N. Guiglielmoni, H. Herlyn, J.-F. Flot, K. Van Doninck, Comparative genomics of bdelloid rotifers: Insights from analyses of highly contiguous genome assemblies, Poster presented at The International Rotifer Symposium–ROTIFERA XVI (2022), Zagreb, Croatia.

63. M. Pagani, C. Ricci, C. A. Redi, "Oogenesis in Macrotrachela quadricornifera (Rotifera, Bdelloidea) I. Germarium eutely, karyotype and DNA content," in *Rotifer Symposium VI: Proceedings of the Sixth International Rotifer Symposium, held in Banyoles, Spain, June 3–8, 1991* (Springer, 1993), pp. 225–230, doi:https://doi.org/10.1007/BF00025843.

64. P. Virtanen, R. Gommers, T. E. Oliphant, M. Haberland, T. Reddy, D. Cournapeau, E. Burovski, P. Peterson, W. Weckesser, J. Bright, S. J. van der Walt, M. Brett, J. Wilson, K. J. Millman, N. Mayorov, A. R. J. Nelson, E. Jones, R. Kern, E. Larson, C. J. Carey, İ. Polat, Y. Feng, E. W. Moore, J. VanderPlas, D. Laxalde, J. Perktold, R. Cimrman, I. Henriksen, E. A. Quintero, C. R. Harris, A. M. Archibald, A. H. Ribeiro, F. Pedregosa, P. van Mulbregt, SciPy 1.0 Contributors, SciPy 1.0: Fundamental Algorithms for Scientific Computing in Python. *Nat. Methods* **17**, 261–272 (2020).

65. S. Andrews, FastQC: A quality control tool for high throughput sequence data (2010), http://www.bioinformatics.babraham.ac.uk/projects/fastqc/.

66. P. Ewels, M. Magnusson, S. Lundin, M. Käller, MultiQC: Summarize analysis results for multiple tools and samples in a single report. *Bioinformatics* **32**, 3047–3048 (2016).

67. S. Chen, Y. Zhou, Y. Chen, J. Gu, fastp: An ultra-fast all-in-one FASTQ preprocessor. *Bioinformatics* **34**, i884–i890 (2018).

68. H. Li, Aligning sequence reads, clone sequences and assembly contigs with BWA-MEM. arXiv:1303.3997 [q-bio.GN] (2013).

69. A. Tarasov, A. J. Vilella, E. Cuppen, I. J. Nijman, P. Prins, Sambamba: Fast processing of NGS alignment formats. *Bioinformatics* **31**, 2032–2034 (2015).

70. F. J. Sedlazeck, P. Rescheneder, M. Smolka, H. Fang, M. Nattestad, A. von Haeseler, M. C. Schatz, Accurate detection of complex structural variations using single-molecule sequencing. *Nat. Methods* **15**, 461–468 (2018).

71. P. Danecek, J. K. Bonfield, J. Liddle, J. Marshall, V. Ohan, M. O. Pollard, A. Whitwham, T. Keane, S. A. McCarthy, R. M. Davies, H. Li, Twelve years of SAMtools and BCFtools. *Gigascience* **10**, giab008 (2021).

72. C. Yang, J. Chu, R. L. Warren, I. Birol, NanoSim: Nanopore sequence read simulator based on statistical characterization. *GigaScience* **6**, gix010 (2017).

73. J. E. Barrick, R. E. Lenski, Genome dynamics during experimental evolution. *Nat. Rev. Genet.* **14**, 827–839 (2013).
